# Limited Evidence for Parallel Evolution Among Desert-Adapted *Peromyscus* Deer Mice

**DOI:** 10.1093/jhered/esab009

**Published:** 2021-03-04

**Authors:** Jocelyn P Colella, Anna Tigano, Olga Dudchenko, Arina D Omer, Ruqayya Khan, Ivan D Bochkov, Erez L Aiden, Matthew D MacManes

**Affiliations:** 1 Department of Molecular, Cellular, and Biomedical Sciences, University of New Hampshire, Durham, NH; 2 Hubbard Genome Center, University of New Hampshire, Durham, NH; 3 Center for Genome Architecture, Department of Molecular and Human Genetics, Baylor College of Medicine, Houston, TX; 4 Center for Theoretical and Biological Physics, Rice University, Houston, TX; 5 Department of Computer Science, Department of Computational and Applied Mathematics, Rice University, Houston, TX; 6 Shanghai Institute for Advanced Immunochemical Studies, ShanghaiTech University, Shanghai 201210, China; 7 School of Agriculture and Environment, University of Western Australia, Perth, WA, Australia; 8 Biodiversity Institute, University of Kansas, Lawrence, KS

**Keywords:** dehydration, desert, parallel evolution, *Peromyscus*, thermoregulation

## Abstract

Warming climate and increasing desertification urge the identification of genes involved in heat and dehydration tolerance to better inform and target biodiversity conservation efforts. Comparisons among extant desert-adapted species can highlight parallel or convergent patterns of genome evolution through the identification of shared signatures of selection. We generate a chromosome-level genome assembly for the canyon mouse (*Peromyscus crinitus*) and test for a signature of parallel evolution by comparing signatures of selective sweeps across population-level genomic resequencing data from another congeneric desert specialist (*Peromyscus eremicus*) and a widely distributed habitat generalist (*Peromyscus maniculatus*), that may be locally adapted to arid conditions. We identify few shared candidate loci involved in desert adaptation and do not find support for a shared pattern of parallel evolution. Instead, we hypothesize divergent molecular mechanisms of desert adaptation among deer mice, potentially tied to species-specific historical demography, which may limit or enhance adaptation. We identify a number of candidate loci experiencing selective sweeps in the *P. crinitus* genome that are implicated in osmoregulation (*Trypsin, Prostasin*) and metabolic tuning (*Kallikrein, eIF2-alpha kinase GCN2, APPL1/2*), which may be important for accommodating hot and dry environmental conditions.

Increasing global temperatures and altered patterns of precipitation threaten biodiversity worldwide (Moritz et al. 2008; Cahill et al. 2013; Urban 2015). Phenotypic plasticity enables an immediate response to changing conditions but adaptation through evolutionary change will be critical for the long-term survival of most species (Hoffman and Sgrò 2011; Cahill et al. 2013). Range shifts upward in elevation and latitude have been documented in a number of terrestrial species and interpreted as a response to warming (Chen et al. 2011; Tingley and Beissinger 2013; Freeman et al. 2018); however, responses vary even among closely related species and populations (Hoffman and Willi 2008; Moritz et al. 2008). Geographic range shifts are often governed by the physiological limits of species, which are in part controlled by genetics and have been shaped by neutral and selective evolutionary forces across many generations.

Population genomics methods enable genome-wide scans for selection to identify genes and molecular pathways that may be involved in local adaptation (Bassham et al. 2018; Garcia-Elfring et al. 2019). For species adapted to similar environments, parallel or convergent evolution can be inferred if a greater number of genes or phenotypes share signatures of selection than would be expected under a purely stochastic model of evolution (e.g., drift). The same gene or suite of genes consistently tied to a specific adaptive phenotype across distantly related taxa is consistent with a signal of convergent evolution. In contrast, for taxa that share a recent common ancestor, signatures of selection at the same loci may reflect parallel selection on either new mutations or shared ancestral variation or similar demographic histories, resulting in the same phenotypic effect. Evidence of convergent or parallel evolution can highlight common loci involved in shared adaptive phenotypes (Rundle et al. 2000; McDonald et al. 2009), while a lack of concerted evolution may identify idiosyncratic evolutionary strategies to achieve the same phenotypic result.

As a model taxon inhabiting varied environments throughout North America (Dewey and Dawson 2001; Bedford and Hoekstra 2015), deer mice (genus *Peromyscus*) are a frequent and productive subject of adaptation studies (e.g., physiological, Storz 2007; behavioral, Hu and Hoekstra 2017; genetic, Cheviron et al. 2012; Storz and Cheviron 2016; Tigano et al. 2020). The physiological similarity of deer mice to house mice (*Mus musculus*), a well-studied biomedical model, further broadens the implications of evolutionary and ecological investigations of *Peromyscus* by linking results to biomedical sciences. The genus *Peromyscus* (*N* = 67 species; mammaldiversity.org) is hypothesized to be the product of rapid ecological radiation across North America (origin ~8 Mya, radiation ~5.71 Mya; Platt et al. 2015), evident in their varied ecological niches and rich species diversity (Glazier 1980; Riddle et al. 2000; Bradley et al. 2007; Platt et al. 2015; Lindsey 2020). *Peromyscus* display tremendous thermoregulatory plasticity and can be found in extreme thermal environments, ranging from cold, high elevations (Pierce and Vogt 1993; Cheviron et al. 2012, 2014; Kaseloo et al. 2014; Garcia-Elfring et al. 2019) to arid, hot deserts (Riddle et al. 2000; MacManes 2017; Tigano et al. 2020). Thermoregulation and dehydration tolerance are complex physiological traits, suggesting that several potential evolutionary routes could lead to the same phenotypic outcome. Within this framework, comparisons among divergent *Peromyscus* species adapted to similar environments may highlight shared adaptive polymorphisms or disparate evolutionary paths to the same phenotype (Cheviron et al. 2012; Hu and Hoekstra 2017; Ivy and Scott 2017; Storz et al. 2019). In cold environments, endotherms rely on aerobic thermogenesis to maintain a constant internal body temperature. Changes in both gene expression and the functional properties of proteins in deer mice (*Peromyscus maniculatus*) adapted to high-altitudes suggest that changes in multiple hierarchical molecular pathways may be common in the evolution of complex physiological traits, such as thermoregulation (Wichman and Lynch 1991; Storz 2007; Cheviron et al. 2012; Storz and Cheviron 2016; Garcia-Elfring et al. 2019). Nonetheless, research focused on thermoregulatory adaptations in high-elevation species may be confounded by concurrent selection on other traits conferring fitness benefits, such as high hemoglobin oxygen-binding affinity (Storz and Kelly 2008; Storz et al. 2010; Natarajan et al. 2015), which is important in low partial pressure (PaO_2_), high-elevation environments. In hot environments, endotherms are challenged with balancing heat dissipation, energy expenditure, and water retention (Anderson and Jetz 2005), resulting in a different suite of behavioral, physiological, and molecular adaptations that enable survival (Schwimmer and Haim 2009; Degen 2012; Kordonowy et al. 2016), but may be confounded by acute or chronic dehydration. Understanding the biochemical mechanisms that enable survival under extreme environmental stress can provide important insights into the nature of physiological adaptation.

Rapid environmental and ecological differentiation among *Peromyscus* species positions these small rodents as models for generating hypotheses surrounding species responses to accelerated warming (Cahill et al. 2013) and the potential for repeated adaptation to similar environments among closely related species. Numerous *Peromyscus* species are adapted to life in hot deserts, with each species and population subject to distinct histories of demographic variation and gene flow. These idiosyncratic histories have a direct impact on evolution, as effective population sizes (*N*_e_) are inextricably linked to the efficacy of selection and maintenance of genetic diversity in wild populations (Charlesworth 2009). Contemporary or historical gene flow may further help or hinder adaptive evolution through adaptive introgression or homogenization, respectively (Coyne and Orr 2004; Morjan and Reiseberg 2004; Tigano and Friesen 2016; Jones et al. 2018). Native to the American West, the canyon mouse (*Peromyscus crinitus*, Figure 1) is a xerocole, highly specialized to life in hot deserts. In the lab, *P. crinitus* can survive in the absence of exogenous water, with urine concentration levels similar to that of desert-adapted kangaroo rats (*Dipodomys merriami*; Abbott 1971; MacMillen 1972; MacMillen and Christopher 1975; MacMillien 1983), but without equivalently specialized renal anatomy (Issaian et al. 2012). Canyon mice also exhibit a lower-than-expected body temperature relative to their size and can enter environmentally induced torpor in response to drought, food limitation, or extreme ambient temperatures (McNab and Morrison 1963; Morhardt and Hudson 1966; McNab 1968; Johnson and Armstrong 1987), which facilitates survival in highly-variable and extreme desert environments. These phenotypes persist for multiple generations in the lab indicating that they have a genetic basis (McNab and Morrison 1968). Cactus mice (*Peromyscus eremicus*) are frequently sympatric with *P. crinitus* and share many of the same adaptations described above for *P. crinitus* (Veal and Caire 1979; Kordonowy et al. 2016). Thus, we expect these 2 species to exhibit similar patterns of molecular adaptation. These 2 desert specialists belong to a monophyletic clade of deer mice, which also includes *Peromyscus merriami, Peromyscus californicus*, *Peromyscus eva*, and *Peromyscus fraterculus*, estimated to have diverged around 5–6 Mya (Platt et al. 2015). Other members of this clade exhibit similar adaptations to desert environments, including urine concentration, reduced water requirements, and environmentally induced torpor (McNab and Morrison 1963; Veal and Caire 1979) suggesting that desert adaptation may represent the ancestral state of this clade. In contrast, the habitat generalist *P. maniculatus* (North American deer mouse) is phylogenetically basal to the 2 desert specialists examined here and has a geographically widespread distribution across North America. *Peromyscus maniculatus* inhabits a wide range of thermal environments, including hot southwestern deserts and cool, high elevations, but desert specialists are not its closest relatives and the species is not generally considered a xerocole. Locally adapted desert populations of *P. maniculatus* (subspecies *P. m. sonoriensis*) in the American Southwest, however, may exhibit patterns of selection similar to that of desert specialists, either through the parallel selective retention of functional ancestral polymorphisms or convergent selection on new mutations. Whole-genome assemblies are publicly available for both *P. eremicus* (PRJEB33593, ERZ119825; Tigano et al. 2020) and *P. maniculatus* (GCA_003704035.1), which positions these species as ideal comparatives against *P. crinitus* to identify genes and regulatory regions associated with desert adaptation, including those unique to desert specialists *P. eremicus* and *P. crinitus.*

**Figure 1.**
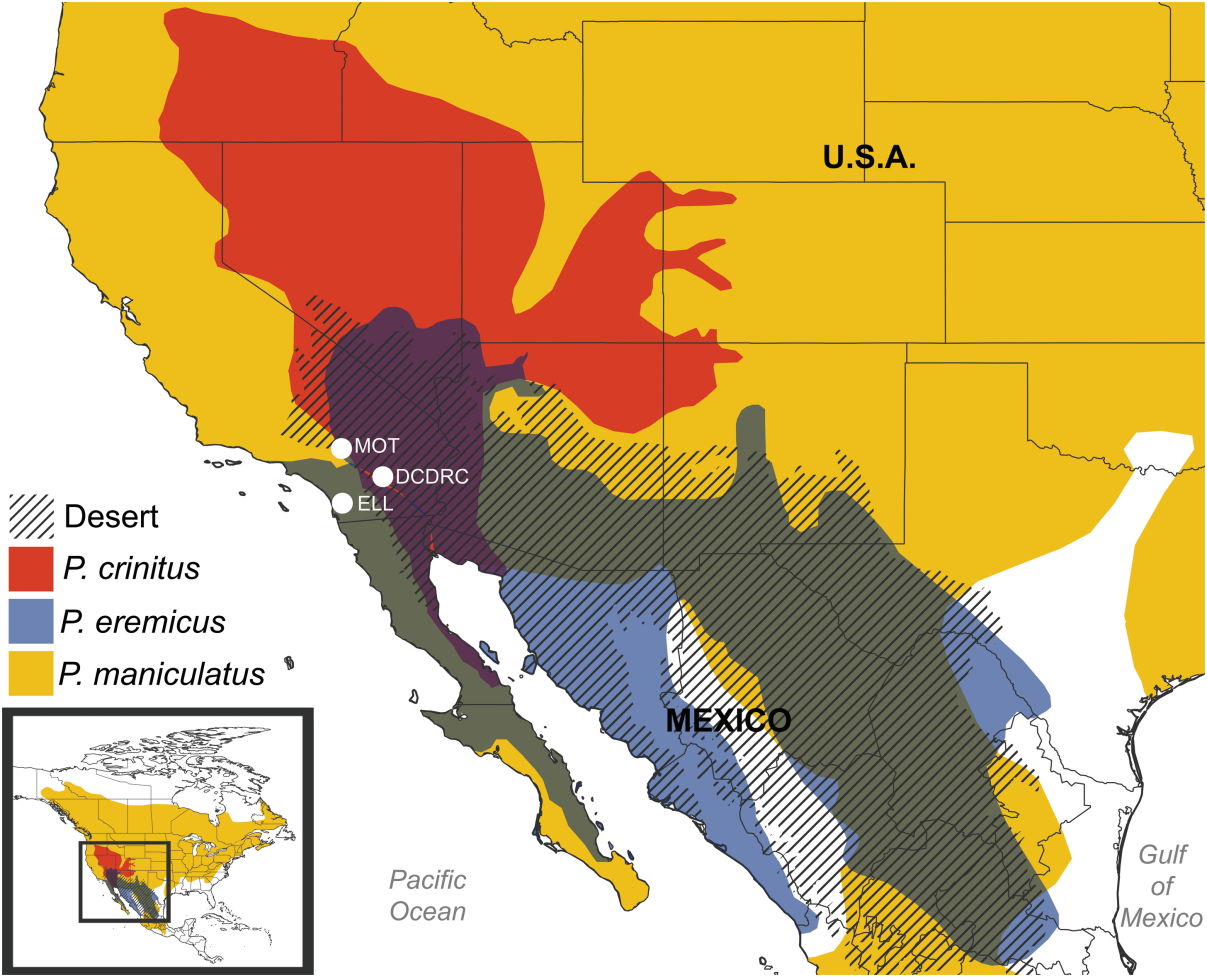
Geographic ranges of the 3 *Peromyscus* species examined in this study. Major North American deserts are denoted by diagonal hashing. *Peromyscus crinitus* range is in red/medium grey, *Peromyscus eremicus* in blue/dark grey, and *Peromyscus maniculatus* in yellow/light grey. Areas of sympatry are denoted by additive color overlap. Collection localities are labeled with white dots and include the Motte Rimrock Reserve (MOT), Elliot Chaparral Reserve (ELL), and Philip L. Boyd Deep Canyon Desert Research Center (DCDRC). See online version for full colors.

Here, we investigate genomic signatures of selection among desert-adapted *Peromyscus*. We contrast signatures of selective sweeps across 3 related *Peromyscus* species, 2 desert specialists (*P. crinitus* and *P. eremicus*), and 1 habitat generalist collected in an arid environment (*P. maniculatus*). We hypothesize that similar genes or functional groups will be under selection in related desert specialist species (*P. eremicus* and *P. crinitus*), due to their shared recent common ancestor and mutual association with hot, arid environments. In contrast, we hypothesize that *P. maniculatus* will show idiosyncratic evolutionary responses, with arid adaptation in this clade having evolved independently in response to local conditions. If similar genes and pathways respond to selection in all 3 species, it would suggest local adaptation of *P. maniculatus* to desert conditions, and potentially, parallel or convergent evolution among divergent *Peromyscus* clades. Given the evolutionary distance of *P. maniculatus* to the 2 desert-adapted species, a shared signature of selection across all 3 species may also indicate that adaptive responses to desert conditions are predictable and can occur repeatedly and potentially on short evolutionary timescales. Finally, we place selective sweep analyses into an evolutionary framework to interpret the varied evolutionary trajectories available to small mammals to respond to changing environmental conditions and to account for demographic and gene flow events.

## Materials and Methods

### De Novo Genome Sequencing and Assembly

Wild mice were handled and sampled in accordance with the University of New Hampshire and University of California Berkeley’s Institutional Animal Care and Use Committee (130902 and R224-0310, respectively) and California Department of Fish and Wildlife (SC-008135) and the American Society of Mammalogists best practices (Sikes and Animal Care and Use Committee of the American Society of Mammalogists 2016).

For the assembly of the *P. crinitus* genome, DNA was extracted from a liver subsample from an individual collected in 2009 from the Philip L. Boyd Deep Canyon Desert Research Center (DCDRC) in Palm Desert, CA. To generate a high-quality, chromosome-length genome assembly for this individual, we extracted high-molecular-weight genomic DNA using a Qiagen Genomic-tip kit (Qiagen, Inc., Hilden, Germany). A 10X Genomics linked-reads library was prepared according to the manufacturer’s protocol and sequenced to a depth of 70× on a HiSeq 4000 (Novogene, Sacramento, CA). 10X Genomics reads were de novo assembled into contigs using *Supernova* 2.1.1 (Weisenfeld et al. 2017). To arrange scaffolds into chromosomes, a Hi-C library for *P. crinitus* was constructed and sequenced on an Illumina NovaSeq 6000 from primary fibroblasts from the T.C. Hsu Cryo-Zoo at the University of Texas MD Anderson Cancer Center. The Hi-C data were aligned to the supernova assembly using *Juicer* (Durand et al. 2016). Hi-C genome assembly was performed using the *3D-DNA* pipeline (Dudchenko et al. 2017) and the output was reviewed using *Juicebox Assembly Tools* (Dudchenko et al. 2018). The Hi-C data are available on www.dnazoo.org/assemblies/Peromyscus_crinitus, where they can be visualized using *Juicebox.js*, a cloud-based visualization system for Hi-C data (Robinson et al. 2018).

Benchmarking Universal Single-Copy Orthologs (*BUSCO* v3, using the Mammalia odb9 database; Simão et al. 2015) and *OrthoFinder2* (Emms and Kelly 2015) were used to assess genome quality and completeness. Genome sizes were estimated for each species using *abyss-fac* (Simpson et al. 2009) and the *assemblathon_stats.pl* script available at https://github.com/ucdavis-bioinformatics/assemblathon2-analysis/. *RepeatMasker* v.4.0 (Smit et al. 2013) was used to identify repetitive elements. The genome was annotated using the software package *MAKER* (3.01.02; Campbell et al. 2014). Control files, protein, and transcript data used for this process are available at https://github.com/macmanes-lab/pecr_genome/tree/master/annotation. We used *Mashmap (-f one-to-one --pi 90 -s 300000*; Jain et al. 2017, 2018) to assess syntenic conservation between *P. crinitus* and *P. maniculatus* genomes, and alignments were plotted with the script *generateDotPlot.pl*. *Peromyscus crinitus* chromosomes were renamed and sorted using *seqtk* (github.com/lh3/seqtk) following the *P. maniculatus* chromosome naming scheme.

For comparative genomics analyses, we generated low-coverage whole-genome resequencing data for 9 *P. crinitus* and 5 *P. maniculatus* individuals collected from arid sites in southern California (Figure 1; Supplementary Table S1). *Peromyscus crinitus* samples were collected from the University of California (UC) DCDRC and *P. maniculatus* were collected further East from the UC Motte Rimrock (MOT) and Elliot Chaparral Reserves (ELL; Figure 1). We also used publicly available low-coverage whole-genome resequencing data from 26 *P. eremicus* individuals, also collected from DCDRC and MOT, that were prepared and sequenced in parallel (Tigano et al. 2020). All samples were collected in 2009, with the exception of 8 *P. eremicus* samples that were collected in 2018. Animals were collected live in Sherman traps and a 25 mg ear-clip was taken from each individual and stored at −80 °C in 95% ethanol. Animals were sampled from arid areas with average monthly temperatures between 9 °C and 40 °C and mean annual rainfall of 15–18 cm. The Biotechnology Resource Center at Cornell University (Ithaca, NY) prepared genomic libraries using the Illumina Nextera Library Preparation kit (e.g., skim-seq). Libraries were sequenced at Novogene (Sacramento, CA) using 150 bp paired-end reads from one lane on the Illumina NovaSeq S4 platform. *fastp* v. 1 (Chen et al. 2018) was used to assess read quality and trim adapters. Sequences from all samples and all species were mapped to the *P. crinitus* reference genome with *BWA* (Li and Durbin 2010) to enable comparative analyses, duplicates were removed with *samblaster v. 0.1.24* (Faust and Hall 2014), and alignments were indexed and sorted using *samtools v. 1.10* (Li et al. 2009).

### Population Genomics

We used the software package *ANGSD v. 0.93* (Korneliussen et al. 2014) to call variants from low-coverage population genomic data from the 3 species (26 *P. eremicus*, 9 *P. crinitus*, and 5 *P. maniculatus*) with high confidence. First, an initial list of high-quality SNPs was identified by analyzing all samples from the 3 species together using the settings: -*SNP_pval 1e-6 -minMapQ 20 -minQ 20 -setMinDepth 20 -minInd 20 -minMaf 0.01*. Then, allele frequencies for each of those high-quality SNPs were calculated independently for each species, with the following filtering steps: a minimum of half (*-minInd*) *P. crinitus* and *P. eremicus* samples and all *P. maniculatus* samples had to meet independent quantity (*-minMapQ*) and quality (*-minQ*) thresholds for each variable site.

Differentiation among species was examined using a multidimensional scaling (MDS) analysis in *ANGSD*. MDS plots were generated in *R* v.3.6.1 (R Core Team 2017) based on the covariance matrix. Cook’s *D* was used to identify outliers (Cook and Weisberg 1984; Williams 1987). As an additional measure of differentiation, we estimated weighted and unweighted global *F*_ST_ values for each species pair using *realSFS* in *ANGSD*. *NGSadmix v. 33* (Skotte et al. 2013) was used to fit genomic data into K populations to parse species-level differences and provide a preliminary screen for genomic admixture under a maximum-likelihood model. Individuals with <90% assignment to a particular species were considered putatively admixed. To examine the impact of coverage on the detection of admixture, we also evaluated coverage distributions among admixed and non-admixed individuals. Nonetheless, expanded sample sizes with greater sequencing depth will be necessary to detail patterns of population structure and introgression. We tested *K* = 1 through *K* = (*N* − 1), where *N* is the number of total individuals examined. *NGSadmix* was run for all species combined and again for each species independently.

We used Pairwise sequential Markovian Coalescent (*PSMC v. 0.6.5-r67*; Li and Durbin 2011) to examine historical demographic changes through time for each species. *PSMC* analyses are not suitable for low-coverage genomes, therefore we used the higher-coverage reads used to generate the high-quality, chromosome-length assemblies for each species (*P. crinitus*, assembly methods detailed above; *P. eremicus*, SAMEA5799953, Tigano et al. 2020; *P. maniculatus*: GCA_003704035.1, Harvard University). High quality reads (*q* > 20; *Skewer*, Jiang et al. 2014) were mapped to their respective de novo assembled reference to identify heterozygous sites. Reference assemblies were then indexed in *BWA*. *Samblaster* removed PCR duplicates and *picard* (http://broadinstitute.github.io/picard/) added a read group to the resulting bam file and generated a sequence dictionary (*CreateSequenceDictionary*) from the reference assembly. For each species, *samtools* was used to sort and index alignments, and variants were called using *mpileup* in *bcftools v1.10.2* (*call*, Li et al. 2009). Consensus sequences were called in *VCFtools v 0.1.16* (*vcf2fq*, Danecek et al. 2011). *PSMC* distributions of effective population size (*N*_e_) were estimated with 100 bootstrap replicates and results were visualized with *gnuplot v. 5.2* (Williams and Kelley 2010), using perl scripts available at https://github.com/lh3/psmc. Output was scaled by a generation time of 6 months (0.5 year, Millar 1989; Pergams and Lacy 2008) and a general mammalian mutation rate of 2.2 × 10^−9^ substitutions/site/year (Kumar and Subramanian 2002).

### Selection and Convergence

We used *Sweepfinder2* (Nielsen et al. 2005; DeGiorgio et al. 2016; Huber et al. 2016) to detect recent selective sweeps as it is compatible with low-coverage whole-genome data. This method performs a composite likelihood ratio (CLR) test to detect deviations from the neutral site frequency spectrum (SFS) that may indicate recent positive selection. *Sweepfinder2* was run on both variant and invariant sites (Huber et al. 2016) for each species, excluding sex chromosomes. Sex chromosomes were excluded for 3 reasons: 1) sex chromosome evolution is both rapid and complex relative to autosomes, 2) we had different sample sizes of each sex across species, and 3) desert adaptations, the focus of this study, are unlikely to be sex-specific. We repeated *Sweepfinder2* analyses on *P. eremicus*, initially analyzed by Tigano et al. (2020), using an improved annotation scheme based on *Peromyscus-*specific data rather than *M. musculus*. Allele frequencies were estimated in *ANGSD* independently for each species and converted to allele counts, and the SFS was estimated in *Sweepfinder2* from autosomes only. Identification of sweeps were based on the pre-computed SFS and the CLR was calculated every 10 000 sites. Per Tigano et al. (2020), a 10 kbp window size was selected as a trade-off between computational time and resolution. CLR values above the 99.9^th^ percentile of the empirical distribution for each species were considered to be evolving under a model of natural selection, hereafter referred to as significant sweep sites. Smaller sample sizes produce fewer bins in the SFS and a lower number of rare alleles may impact both the overall SFS and local estimate surrounding sweep sites; therefore, we explored the impact of sample sizes on *Sweepfinder2* results by downsampling the number of genomes analyzed for each species to 5 individuals (the total number of low-coverage genomes available for *P. maniculatus*) and compared sweep results between downsampled and all-sample datasets for the 3 smallest chromosomes: 21, 22, and 23.

For each species, mean Tajima’s *D* was calculated across the entire genome in nonoverlapping windows of 10 and 1 kbp in *ANGSD*. Nucleotide diversity (π) was also calculated in 10 and 1 kbp windows and corrected based on the number of sites genotyped (variant and invariant) per window. Tajima’s *D* and π are expected to be significantly reduced in regions surrounding selective sweeps (Smith and Haigh 1974; Kim and Stephan 2002), therefore we also used a Mann–Whitney test (*P* < 0.05, after a Bonferroni correction for multiple tests) to measure significant deviations from the global mean in 1 and 10 kbp flanking regions surrounding significant sweep sites. We also examined *D* and π for flanking regions surrounding 27 candidate genes identified in a previous transcriptomic investigation of *P. eremicus* and potentially involved in dehydration tolerance (MacManes 2017; Supplementary Table S2). Candidate loci include aquaporins (*N* = 12), sodium-calcium exchangers (*SLC8a1*), and *Cyp4* genes belonging to the Cytochrome P450 gene family (*N* = 14). We used custom python scripts to functionally annotate (I) the closest gene to each significant sweep site, (II) the nearest upstream and downstream gene, regardless of strand (sense/antisense), and (III) the nearest upstream and downstream gene on each strand. Dataset I follows the general assumption that proximity between a significant sweep site and a protein-coding gene suggests interaction. Dataset II represents an extension of that model by encompassing the most proximal gene in each direction. Because *Sweepfinder2* is based on unphased data mapped to a consensus sequence and our data is unphased, we do not have information indicating on which strand a significant sweep site occurs. Therefore, dataset III encompasses strand-uncertainty by including the 2 nearest genes to a significant sweep site on both strands. It should be noted that the genes identified in smaller datasets (I, II) are nested within the larger datasets (II, III) and by definition, the larger datasets include more noise, which may dilute a signature of parallel evolution, but may better capture the true signal of selection. Hence, it is important to critically examine numerous hierarchical gene subsets. Without a linkage map, these analyses remain exploratory and can be better refined with estimates of linkage disequilibrium, linkage block sizes, and gene density in future investigations. We tested genes from each dataset for functional and pathway enrichment in Gene Ontology (GO) categories using *Panther v. 15.0* (Mi et al. 2017) and extracted GO terms for each enriched functional group. We used *M. musculus* as a reference and a Bonferroni correction for multiple tests (*P* < 0.05) to correct for false discoveries. Enriched GO terms were summarized and visualized in *REVIGO* (Reduce and Visualize Gene Ontology, Supek et al. 2011) implemented at http://revigo.irb.hr/. As a test for similar evolutionary responses to desert environments, overlap in the gene names and enriched GO terms associated with significant selective sweeps was assessed for each dataset. Overlap was visualized in the R package *VennDiagram* (Chen and Boutros 2011). To test for convergence, we used a Fisher’s Exact test (*P* < 0.05) in the *GeneOverlap* package (Shen 2016) in R to assess whether gene or enriched GO term overlap between species was greater than expected based on the total number of genes/GO terms in the genome. To determine if signatures of selection were driven by differences in sequencing depth, we calculated local coverage in 10 kbp windows surrounding significant sweep sites and averaged local coverage estimates across all sweeps on a single chromosome, using a modification of https://github.com/AdamStuckert/Ranitomeya_imitator_genome/blob/master/GenomeAssembly/DuplicateOrthologWorkbook.md. Local coverage surrounding significant sweep sites for each chromosome were compared to the chromosomal average calculated for each species (*samtools coverage --min-MQ 20, --region chr*).

To compare patterns of gene family expansion and contraction potentially involved in adaptation within the genus *Peromyscus*, we analyzed 14 additional genomes, including 10 *Peromyscus* species and 4 near outgroup rodent species: *Microtus ochrogaster, Neotoma lepida, Sigmodon hispidus*, and *M. musculus* (Supplementary Table S3). To prevent bias driven by variable assembly qualities, samples with <70% complete mammalian BUSCOs were excluded from downstream analyses, resulting in the final analysis of 10 species (Supplementary Table S3). Groups of orthologous sequences (orthogroups) were identified in *Orthofinder2*. Invariant orthogroups and groups that varied by more than 25 genes across taxa (custom python script: ortho2cafe.py) were excluded. Our rooted species tree, estimated in *Orthofinder2*, was used to calculate a single birth-death parameter (lambda) and estimate changes in gene family size using *CAFE v.4.2.1* (Han et al. 2013). Results were summarized using the python script *cafetutorial_report_analysis.py* available from the Hahn Lab at https://github.com/hahnlab/CAFE.

## Results

### Chromosome-Length Genome Assembly: *P. crinitus*

Linked reads combined with Hi-C scaffolding produced a high-quality, chromosome-length genome assembly for *P. crinitus*. Our assembly has a contig N50 of 137 026 bp and scaffold N50 of 97 468 232 bp, with 24 chromosome-length scaffolds. The anchored sequences in the 3 *Peromyscus* genome assemblies were as follows: *P. crinitus* genome ~2.27 Gb, *P. eremicus* ~2.5 Gb, and *P. maniculatus* ~2.39 Gb (Table 1). Our assembly has high contiguity and completeness and low redundancy, as demonstrated by the presence of 89.3% complete BUSCOs, 0.9% of duplicates, and 9.0% missing, excluding unplaced scaffolds. As anticipated based on karyotypic analyses (Smalec et al. 2019), we found no significant variation in chromosome number or major interchromosomal rearrangements between *P. crinitus* and *P. maniculatus* (Supplementary Figure S4). We annotated 17 265 total protein-coding genes in the *P. crinitus* genome. Similar to other *Peromyscus* species, LINE1 (long interspersed nuclear elements) and LTR (long terminal repeats) elements comprised 22.7% of the repeats in the *P. crinitus* genome, with SINEs (short interspersed nuclear elements) representing an additional 9.6% (Supplementary Table S5). Although similar to other *Peromyscus* species, *P. crinitus* has the greatest total repeat content (>37%; see Tigano et al. 2020, Supplementary Table 2).

**Table 1.** Assembly stats, genome size, and global Tajima’s *D* and pi (1 kbp windows) for each *Peromyscus* species

Species	*N*	Scaffold N50	Contig N50	Size (Gb)^a^	Size (Gb)^b^	Taj. *D*	π
*Peromyscus crinitus*	9	94 816 992	204 461	2.27	2.28	−0.69	0.005
*Peromyscus eremicus*	26	119 957 392	76 024	2.45	2.54	−1.27	0.007
*Peromyscus maniculatus*	5	115 033 041	42 400	2.33	2.44	−1.62	0.012

^a^abyss-fac estimate.

^b^assemblathon estimate.

### Population Genomics

MDS analysis parsed the 3 species into 3 well-separated clusters and identified no outliers or evidence of admixture (Supplementary Figure S6). *NGSadmix* identified all 3 species as a single group (*K* = 1) with the highest likelihood, but a 3-population model neatly parsed the 3 species as expected (Supplementary Figure S7, Supplementary Table S8). *NGSadmix* analysis, which is more sensitive to low sample sizes than MDS analyses, showed putative admixture in *P. crinitus* with at least 3 individuals displaying 11–27% ancestry from *P. eremicus* and additional material from *P. maniculatus* (4–16%). Variable samples sizes may impact assignment certainty and expanded sequencing of additional *Peromyscus* species and populations will be required to identify potential sources of introgressed material. Four *P. eremicus* individuals had <90% assignment probability to the *P. eremicus* species cluster, with a maximum of 15% assignment to a different species cluster. Identification of admixture in both species was not biased by differences in coverage, as low (2×), medium (8×), and high coverage (17×) samples were found to be admixed at a <90% assignment threshold (Supplementary Figure S9). No *P. maniculatus* individuals were identified as admixed.

**Figure 2.**
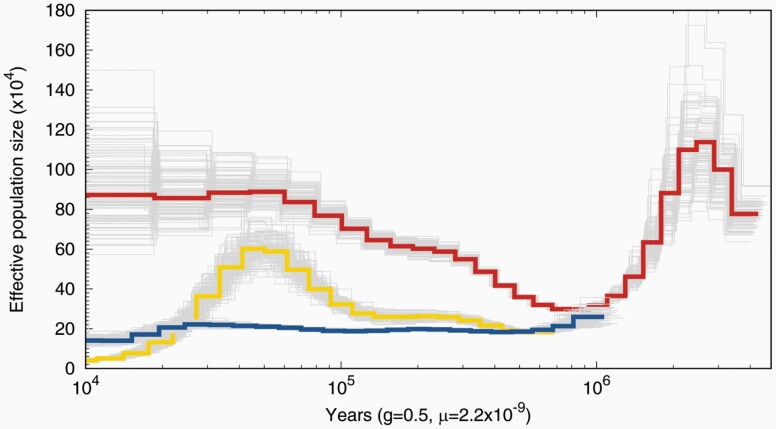
Distributions of effective population size (*N*_e_) through time for *Peromyscus crinitus* (red/top), *Peromyscus eremicus* (blue/bottom), and *Peromyscus maniculatus* (yellow/middle) based on a generation time of 6 months (0.5 years) and a general mammalian mutation rate of 2.2 × 10^−9^ substitutions/site/year. Note that the *P. maniculatus* genome was sequenced from a captive individual and therefore does not reflect natural populations’ trends of this species. CLR scores for *P. crinitus* based on *Sweepfinder2* results. Values above the horizontal red line surpass the 99.9^th^ percentile. The top 5 or fewer unique genes are labeled for each chromosome. See online version for full colors.

*PSMC* estimates of historical demography (>10 kya, Nadachowska-Brzyska et al. 2016) show greater variance and a higher overall *N*_e_ for *P. crinitus* relative to *P. eremicus* (Figure 2). Demographic estimates for *P. maniculatus* are included as an additional comparison but should be interpreted with caution as they are based on sequence data from a captive-bred individual and may not accurately reflect the demography of wild populations.

### Selection and Convergence

*Sweepfinder2* results were generally consistent across downsampled (*N* = 5) and all-sample datasets (9 *P. crinitus*, 26 *P. eremicus*). For example, there were no significant sweep sites identified on chromosome 21 for *P. crinitus* using either dataset, and although more sweeps were significant on chromosome 23 for the downsampled dataset (16 vs. 8), 15 of the 16 sweeps were proximal to a single protein-coding gene (*S15A3*) that was also identified as experiencing a significant sweep when using all available data. All genes proximal to significant sweep sites in the downsampled *P. crinitus* dataset were also identified when all samples were analyzed. Six additional genes were identified as experiencing selective sweeps when the complete dataset was evaluated. Results for *P. eremicus* were slightly less consistent: identical numbers of sweep sites were detected on chromosomes 22 and 23 with additional significant sweep sites identified on chromosome 21 when all samples were examined. The majority of sweep sites on chromosome 21 in *P. eremicus* were proximal to *G3P*, which represented a significant sweep in both downsampled and all-sample datasets; however, there were 8 additional protein-coding genes proximal to significant sweep sites that were detected using the downsampled dataset, but not detected when all samples were included in the analysis (e.g., *Peripherin-2, BICRAL, Mrps18b*). We hypothesize that population structure may increase inconsistency in these results, as the 26 *P. eremicus* samples represent 2 populations (MOT and DCDRC) and 3 distinct collection events (MOT 2018, DCDRC 2009, and DCDRC 2018; Supplementary Table S1), whose representation in the reduced dataset may vary due to different sample sizes and random selection of downsampled individuals. While the study design does not allow us to distinguish between type 1 and type 2 statistical errors, we hypothesize that inconsistencies are related to differences in statistical power, with greater power in the full datasets relative to the subsampled datasets.

**Figure 3.**
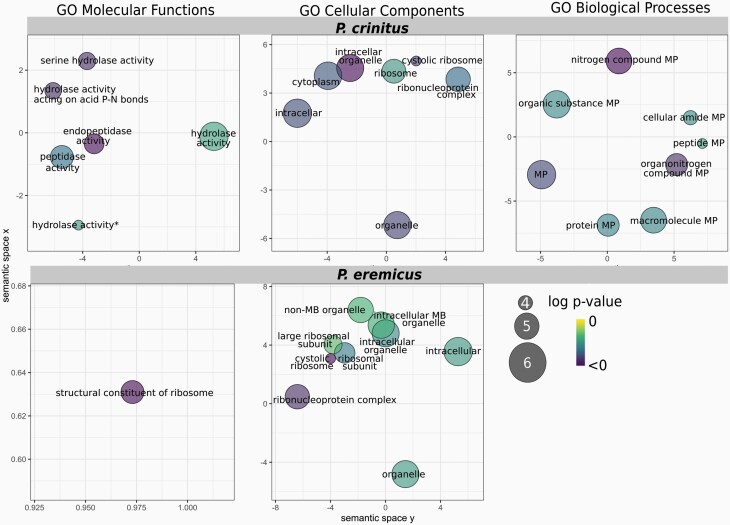
*REVIGO* plots of enriched functional groups for *Peromyscus crinitus* (top row) and *Peromyscus eremicus* (bottom row) based on functional annotation of the 2 nearest protein-coding genes to each site (dataset II) identified as the subject of a selective sweep. Darker colors indicate greater significance. MP, metabolic process, MB, membrane-bound. See online version for full colors.

Within *P. crinitus* we identified a total of 209 significant sweep sites (Supplementary Table S10), with 104 sites localized on chromosomes 9 and 16 experiencing major selective sweeps (Figure 3). We found 239 total significant sweep sites for *P. eremicus* (Supplementary Table S11, Supplementary Figure S12). Despite the large size of chromosome 1 and strong signature of selective sweeps in *P. eremicus*, we found no significant sweep sites on this chromosome for *P. crinitus*. Finally, we identified a total of 213 significant sweep sites for *P. maniculatus* (Supplementary Table S13), with 103 sites located on chromosome 4. Despite general chromosomal synteny among *Peromyscus* species (Supplementary Figure S4; Tigano et al. 2020), the chromosomal distribution of sweep sites differed among species. For example, *P. eremicus* had at least one significant sweep detected on every chromosome, while sweeps were only detected on 8 and 13 chromosomes in *P. maniculatus* and *P. crinitus*, respectively. We found a number of sweep sites were concentrated on chromosome 9 for both desert specialist species, with additional significant sweep sites for *P. crinitus* localized on chromosome 16 (Figure 3, Supplementary Figure S12; Supplementary Tables S10 and S11). Sweeps in *P. eremicus* were widespread across the genome, with a large peak (56 significant sweep sites) on chromosome 1 (Supplementary Table S11; Supplementary Figure S12). *Peromyscus maniculatus* sweeps fell primarily on chromosomes 4 and 20 (Supplementary Table S13). The chromosomal distribution of significant sweep sites does not appear to be driven by differences in coverage (Supplementary Table S14). Average sequencing depth for 10 kbp windows surrounding each significant sweep site did not differ significantly from the global average sequencing depth for *P. crinitus* (*P* = 0.25) or *P. maniculatus* (*P* = 0.28). Local (10 kbp window) coverage surrounding significant sweeps for *P. eremicus* were less consistent (Supplementary Tables S10, S11, S13; Supplementary Results). Fourteen of 232 significant sweep sites (6%) in *P. eremicus* exhibited extreme local sequencing depths of 0 or >1000, leading to a significant difference between overall mean sequencing depth and sequencing depth surrounding sweep sites (*P* = 0.03), with sweep windows exhibiting higher sequencing depths on average (73 vs. 25). If the 14 anomalous values are excluded, sequencing coverage does not differ (*P* = 0.37) for *P. eremicus*.

**Figure 4.**
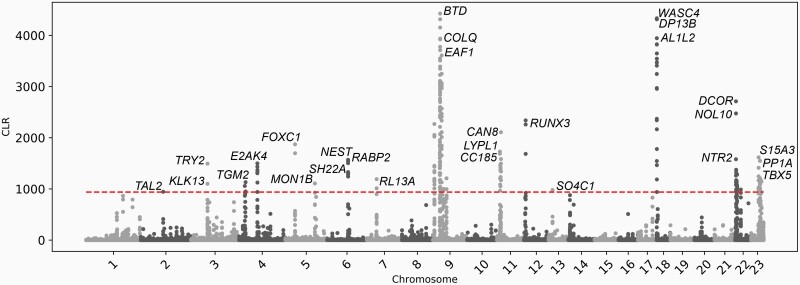
CLR scores for *Peromyscus crinitus* based on *Sweepfinder2* results. Values above the horizontal dashed line surpass the 99.9^th^ percentile. The top 5 or fewer unique genes are labeled for each chromosome.

The effect of a selective sweep extends beyond the specific site identified as the target of positive selection; hence, putative outliers (CLR > 99.9%) are indicative of a sweep in the 10 kbp window but the specific nucleotides under selection cannot be identified. Coding genes represent only a small proportion of the genome, therefore, if a sweep site does not fall within a coding region, we assume that the target of selection may be a regulatory element that affects gene expression of proximal coding genes. Under this assumption, we hierarchically examined protein-coding genes most proximal to each sweep site and chose not to set a distance threshold, as regulatory elements are known to affect genes up to hundreds of kb away (e.g., Wallbank et al. 2016). On average, the distance from a sweep site to the nearest coding gene was 45 kbp in *P. crinitus* (range: 31–439 122 bp, median = 5 kbp) and much greater for both *P. maniculatus* (average: 152 kbp; range: 190–513 562 bp, median = 111 kbp) and *P. eremicus* (average: 117 kbp; range: 38–1 423 027 bp; median: 35 kbp), despite high assembly qualities for all species and identical methods of gene annotation. For both *P. eremicus* and *P. maniculatus*, only 2 significant sweep sites were localized within protein-coding genes (*P. eremicus:* meiosis-specific with OB domain-containing protein, harmonin; *P. maniculatus:* dehydrogenase/reductase SDR family member 7B and zinc finger protein 217; Table 2). In contrast, for *P. crinitus* 12 significant sweep sites fell within 19 distinct candidate loci, many of which code for multiple alternatively spliced transcripts (Table 2). Among the significant sweep sites localized within *P. crinitus* coding sequences, we identified 19 enriched GO terms (3 biological process [BP], 9 molecular function [MF], 7 cellular component [CC]), with functionality ranging from “proteolysis” to “hydrolase” activity (Figure 4; Supplementary Table S15). Functional examination of candidate loci identified solute regulation as a key function, with genes pertaining to calcium (*Trypsin-2* [*PRSS2*]) and zinc (*Kallikrein-4* [*KLK4*]) binding and sodium regulation (*Prostasin* [*PRSS8*]) indicated as under selection.

**Table 2.** Significant sweep sites localized within protein-coding genes for each *Peromyscus* species

Spp.	Chr.	Pos.	Gene	Protein	General function	Dir.
*Peromyscus maniculatus*	4	145409180	*ZNF217*	Zinc finger protein 217	DNA-binding transcription factor, transcription regulation, zinc binding	−
	20	36260251	*DHRS7B*	Dehydrogenase/reductase SDR family member 7B	Oxidoreductase activity	−
*Peromyscus eremicus*	1	42451454	*Ush1c*	Harmonin	Mechanotransduction in cochlear hair cells	−
	8	67956	*MEIOB*	Meiosis-specific with OB domain-containing protein	Meiosis	+
*Peromyscus crinitus*	3	52113514	*PRSS2*	Trypsin-2	Calcium ion binding	+
			*KLK13*	Kallikrein-13	Protein processing, proteolysis, reg. of IGF	+
			*PRSS8*	Prostasin	Sodium balance	+
			*KLK4*	Kallikrein-4	Zinc ion binding, proteolysis	+
			*PRTN3*	Myeloblastin	Degrades collagen (I, III, IV), elastin, fibronectin, laminin, vitronectin; blood coagulation, immune response	+
			*KLK14*	Kallikrein-14	Varied (epidermis morphogenesis)	+
			TRYP_PIG^a^	Trypsin	Calcium ion binding, proteolysis	+
			*KLK6*	Kallikrein-6	Varied (collagen catabolism, tissue regen)	+
			*CELA2A*	Chymotrypsin-like elastase family member 2A	Cleavage and elastin hydrolase, proteolysis	+
	4	57673659	*EIF2AK4*	eIF-2-alpha kinase GCN2	Metabolic stress sensing protein kinase, role in ISR required for adaptation to amino acid starvation, protein synthesis repression	−
	6	66203934	*Nes*	Nestin	Brain, eye development (neg. reg. catalytic activity)	−
	9	23303147	*DENND64*	DENND64	Endocytic recycling pathway component	+
		23323150	*DENND64*	DENND64	Endocytic recycling pathway component	+
		43305800	*Nynrin*	NYNRIN	Nucleic acid binding	+
		22243007	*Parg*	Poly(ADP-ribose) glycohydrolase	Prevent detrimental accumulation of poly(ADP-ribose) upon prolonged replicative stress	−
		22283012	*NCOA4*	Nuclear receptor coactivator 4	Androgen receptor (iron ion homeostasis)	−
		22303015	*Oxnad1*	Oxidoreductase NAD-binding domain-containing protein 1	Oxidoreductase activity	−
	18	450151	*APPL2*	DCC-interacting protein 13-beta	Varied (cold acclimation, diet induced thermogen., glucose homeostasis, neg. reg. of insulin response/fatty acid oxidation/glucose import, pos. reg. of cold-induced thermogen.)	−
			*APPL1*	DCC-interacting protein 13-alpha	Varied. (insulin receptor signaling pathway, pos. reg. of glucose import)	−
		460153	*APPL2*	DCC-interacting protein 13-beta	Varied (cold acclimation, diet induced thermogen., glucose homeostasis, neg. reg. of insulin response/fatty acid oxidation/ glucose import, pos. reg. of cold-induced thermogen.)	−
			*APPL1*	DCC-interacting protein 13-alpha	Varied (insulin receptor signaling pathway, pos. reg. of glucose import)	−
	23	28065942	*Tctn1*	Tectonic-1	Neural development	+

Species (Spp.), chromosome (Chr.) sweep position (Pos.), gene name, protein, general function (based on UniProt database: uniprot.org), and direction (Dir.) of gene transcription. thermogen., thermogenesis; neg., negative; pos., positive; reg., regulation; IGF, insulin-like growth factor; ISR, integrated stress response.

^a^No gene name alternative available.

Here, we report the results for dataset II, as this dataset 1) ensures the inclusion of the most proximal gene under selection, by including the most proximal gene on each strand, and 2) reduces noise associated with dataset III, which includes 4 genes proximal to each sweep site. Results for datasets I and III are addressed in more detail in the Supplementary Material. Examination of dataset II in *P. crinitus* identified 121 unique genes and 26 enriched GO terms (8 BP, 10 MF, 8 CC), with functionality pertaining to metabolism (e.g., “protein metabolic process,” “organonitrogen compound metabolic process,”, “peptide metabolic process”) and ribosomes (Figure 4; Supplementary Tables S10 and S15). For *P. eremicus,* we identified 202 unique genes and 14 enriched GO terms (0 BP, 1 MF, 13 CC) associated with selective sweeps, with functionality centered around ribosomes (Supplementary Table S11 and S16). For *P. maniculatus,* we identified 215 unique genes and 8 enriched GO terms (0 BP, 1 MF, 7 CC) associated with selective sweeps (Supplementary Tables S13 and S17). Two genes and 7 enriched GO terms that were proximal to sweep sites were shared between the 2 desert specialists, but the number of shared genes was not significantly different from what is expected by chance alone. Functional enrichment of *P. eremicus* and *P. maniculatus* across all datasets was limited to ribosomes (e.g., “structural constituent of ribosome,” “cytosolic ribosome,” “ribosomal subunit”; Figure 4; Table 3, Supplementary Tables S15–S17). In contrast, functionality of enriched GO terms for *P. crinitus* centered on metabolic processes, including protein breakdown, hydrolysis, and cellular functionality (e.g., “organelle,” “intracellular,” “cytoplasm”; Figure 4; Supplementary Table S15), in addition to ribosomes.

**Table 3.** Functional annotation of proximal gene names and enriched GO terms associated with significant selective sweeps and shared between desert-adapted *Peromyscus crinitus* and *Peromyscus eremicus*

	Data set	Gene name / GO term	Function	Protein/class
Gene names	I	none	—	—
	II	*BTD*	Hydrolase, biotin transport/metabolism	Biotinidase
		*RL36*	Ribosomal protein, translation	60S ribosomal protein L36
	III	*BTD*	Hydrolase, biotin transport/metabolism	Biotinidase
		*ENV*	Zn binding, virion attachment	Envelope glycoprotein
		*H3X*	DNA binidng, protein heterodimerization	Putative histone H3.X
		*RL15*	RNA binding, ribosome constituent, translation	60S ribosomal protein L15
		*RL36*	Ribosomal protein, translation	60S ribosomal protein L36
		*RPC1*	Zn/Mg binding, immune response	DNA-directed RNA polymerase III subunit RPC1
		*RS2* ^a^	Ribosomal protein, enzyme binding	40S ribosomal protein S2
		*RS26*	mRNA binding, ribosome, translation	40S ribosomal protein S26
		*TM38B*	Rapid Ca^2+^ release, K^+^ channel, ossification	Trimeric intracellular cation channel type B
		*TRY2*	Ca^2+^ binding, collagen catabolism, proteolysis, cell growth	Trypsin-2
Enriched GO terms	I	none	—	—
	II	GO:0005622	Intracellular	Cellular component
		GO:0005840^a^	Ribosome	Cellular component
		GO:0022626^a^	Cystolic ribosome	Cellular component
		GO:0043226	Organelle	Cellular component
		GO:0043229	Intracellular organelle	Cellular component
		GO:0044391^a^	Ribosomal subunit	Cellular component
		GO:1990904^a^	Ribonucleoprotein complex	Cellular component
	III	GO:0003735^a^	Structural constituent of ribosome	Molecular function
		GO:0022626^a^	Cystolic ribosome	Cellular component
		GO:0022625^a^	Cystolic large ribosomal subunit	Cellular component
		GO:0044391^a^	Ribosomal subunit	Cellular component
		GO:0005840^a^	Ribosome	Cellular component
		GO:0015934	Large ribosomal subunit	Cellular component
		GO:1990904^a^	Ribonucleoprotein complex	Cellular component

^a^Gene names or GO terms also experiencing selective sweeps in *Peromyscus maniculatus*.

**Figure 5.**
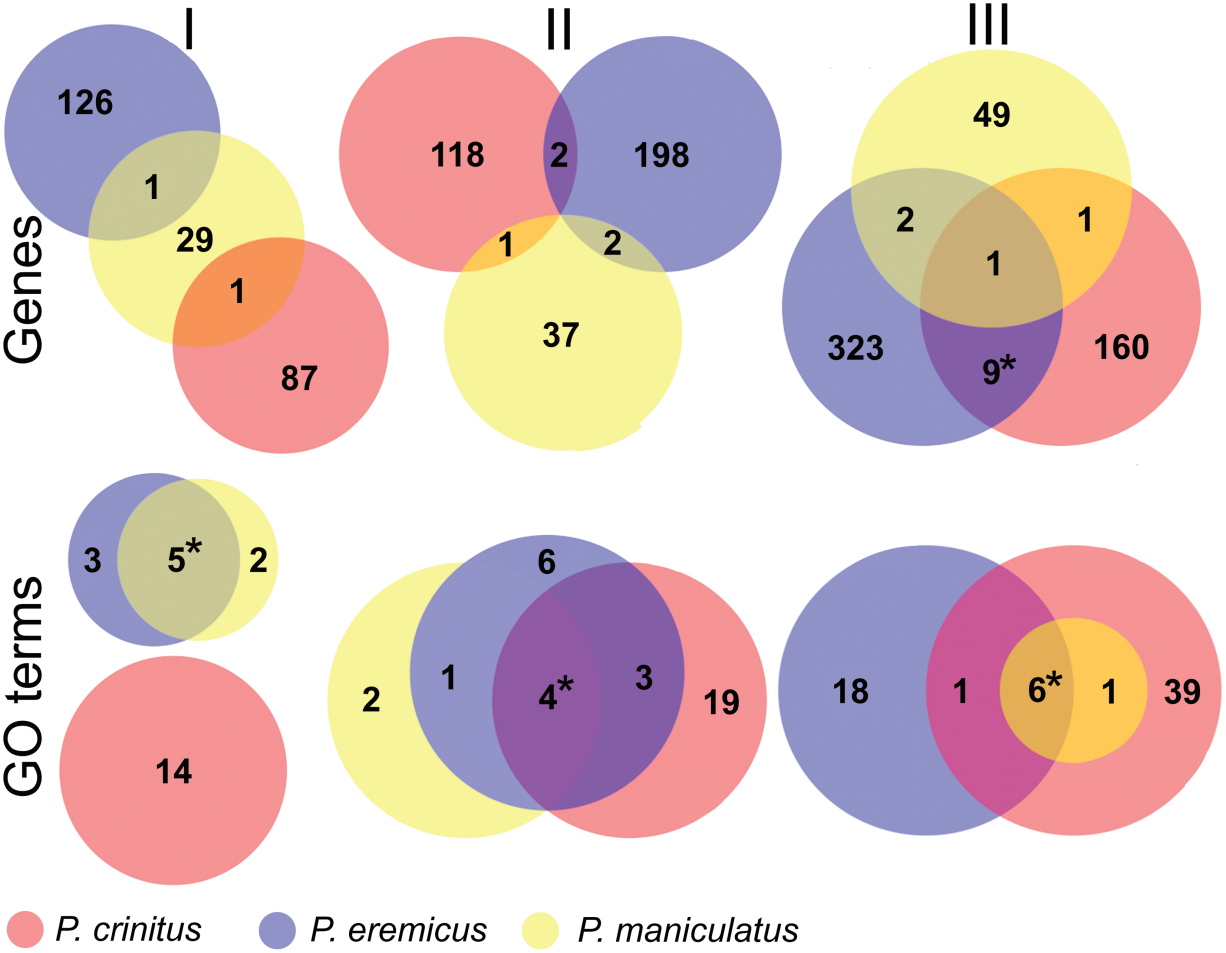
Overlap in proximal gene names (top row) and enriched GO terms (bottom row) for datasets I (left column), II (center), and III (right). * indicates significant overlap between species. See online version for full colors.

*Peromyscus eremicus* and *P. maniculatus* shared significant overlap (*P* < 0.05) in enriched GO terms across all hierarchical data subsets (I, II, III; Figure 5). Significant overlap of enriched GO terms was also detected between *P. crinitus* and both other *Peromyscus* species for datasets II and III only, with no overlap detected for dataset I (Figure 5). Significant overlap between desert specialists *P. eremicus* and *P. crinitus* was only detected in dataset III. Overall, GO terms and genes associated with ribosomal functionality were frequently shared among all species examined, but a unique pattern of selection was not shared among desert specialists.

Species tree estimates (Supplementary Figure S18) were consistent with previous phylogenetic investigations (Bradley et al. 2007). *Peromyscus crinitus* and *P. eremicus* are sister in our species tree, but note that a number of intermediate taxa remain unsampled (e.g., *P. merriami*, *P. californicus*). Among the species examined here, the 2 desert specialists are part of a larger clade of desert-adapted *Peromyscus* to the exclusion of *P. maniculatus*. The *maniculatus* clade is comprised of *P. leucopus, P. polionotus*, and *P. maniculatus*, and the *nasutus-attwateri* clade is most basal within *Peromyscus* (Supplementary Figure S18), consistent with Platt et al. (2015). For the *Peromyscus* genus, we found 19 925 gene families that had experienced contractions, 502 expansions, and 12 families that were evolving rapidly. However, we found no gene families experiencing significant expansions, contractions, or rapid evolution below the genus level.

Average Tajima’s *D* (1 kbp windows) was negative for all species and ranged from −0.69 to −1.61. *Peromyscus crinitus* had the lowest Tajima’s *D* value and *P. maniculatus* the highest (Supplementary Figures S19–S20). Global pairwise *F*_ST_ between species ranged from 0.20 to 0.27 (unweighted: 0.12–0.17). Mean global π (1 kbp windows) was 0.005 (±0.005) for *P. crinitus*, 0.007 (±0.007) for *P. eremicus*, and 0.012 (±0.010) for *P. maniculatus* (Supplementary Figure S21). Both Tajima’s *D* and π for 1 and 10 kbp flanking regions surrounding significant selective sweep sites were significantly higher than the global average for each species (Supplementary Table S20). We only detected a significant reduction in π surrounding significant sweep sites in *P. maniculatus*. Tajima’s *D* for flanking regions surrounding the a priori candidate loci identified by MacManes (2017) were also significantly more positive in all 3 species (Supplementary Table S20).

## Discussion

Continued and accelerating environmental change increases the exigency of accurately predicting species responses to anthropogenic climate change. Adaptive evolutionary responses vary among species and populations, even when subjected to similar environmental selective pressures (Bi et al. 2019; Garcia-Elfring et al. 2019). Evidence of parallel de novo molecular changes or selective retention of shared ancestral variation can highlight genes or genomic regions, including but not limited to functional variants, haplotypes, or structural features of the genome, that may be key to adaptation. Alternatively, the same adaptive phenotype can evolve through alternative evolutionary strategies. Thus, it is possible, even among related species, that adaptation to similar environmental conditions will not exhibit similar patterns of molecular evolution despite similar adaptive phenotypes. We analyzed genome-wide patterns of selective sweeps among 3 species of deer mice within the North American genus *Peromyscus* to identify candidate loci involved in heat and dehydration tolerance. We hypothesized that desert specialists, *P. crinitus* and *P. eremicus,* would share genes or pathways associated with selective sweeps that were not shared with phylogenetically ancestral *P. maniculatus.* These patterns would be indicative of parallel selection (and therefore, parallel evolution) on either de novo mutations or shared ancestral variation. Given the suite of desert adaptations shared by these species, shared signatures of selection may relate to survival in high-temperature, low-water environments. Additionally, we hypothesized that shared patterns of selective sweeps and enriched functional groups across all 3 species, if present, would highlight candidate loci underpinning local adaptation of *P. maniculatus* to arid conditions and potentially identify common loci involved in the repeated evolution of desert adaptation. Although the species examined here are monophyletic, the 2 desert specialists share a more recent ancestor (Supplementary Figure S18) and there are number of unsampled taxa that phylogenetically separate the desert specialists from *P. maniculatus* (e.g., *P. merriami*, *P. californicus*). For this reason, we cannot distinguish between parallel and convergent evolution, and without evidence of ancestral divergence followed by reconvergence, we will discuss shared signatures of selection as parallel evolution hereafter.

Overall, we did not find support for parallel evolution among desert specialist species, but we identified a number of candidate loci that may be important to desert adaptation in *P. crinitus*. Instead of a shared mechanism of heat and dehydration tolerance, we hypothesize that the 2 desert specialists examined here may have adapted to similar environments through divergent molecular mechanisms, with *P. crinitus* potentially responding through genomic changes to protein-coding genes and *P. eremicus* through transcriptional regulation of gene expression. This hypothesis is based on the lack of overlap in selective sweeps between desert specialists, the proximity of sweeps to protein-coding genes in *P. crinitus* relative *to P. eremicus*, and previous gene expression results for *P. eremicus* (Kordonowy and MacManes 2017; MacManes 2017). Molecular flexibility of thermoregulatory responses may have catalyzed the radiation of *Peromyscus* in North America by enabling rapid exploitation of novel thermal environments. Finally, the application of an evolutionary lens to the interpretation of genomic patterns of selection, particularly one that integrates historical demography and gene flow, can help parse varied evolutionary mechanisms (parallel vs. convergent, genomic vs. transcriptomic) of molecular adaptation.

### Limited Evidence of Parallel Evolution

Identification of similar genes or functional groups under selection in different species adapted to similar environments can provide evidence in support of parallel evolution. In contrast, we found limited evidence of parallel evolution among desert-adapted *Peromyscus*. Few to no enriched GO terms overlapped between desert specialists (Figure 5). Only GO terms relating to ribosomes (e.g., “ribosome,” “ribosomal subunit,” “cystolic ribosome,” etc.) overlapped between all 3 *Peromyscus* species examined, with the most significant overlap in GO terms occurring between *P. eremicus* and *P. maniculatus*. Although *P. maniculatus* are not generally xerocoles, the individuals sequenced here were collected in arid regions of southern California (subspecies *P. m. sonoriensis*). Therefore, the shared signature of selection on ribosomes across all examined *Peromyscus*, whether it reflects parallel evolution or the selective retention of shared ancestral polymorphisms, may be associated with adaptation to hot and dry conditions or more broadly relate to thermoregulatory plasticity among *Peromyscus* rodents. Few genes proximal to selective sweeps were shared among all species, with only one instance of significant overlap: 10 genes were shared between the 2 desert specialists under dataset III (Figure 5). Although dataset III may be confounded by excess noise through the inclusion of additional protein-coding genes, this signature is potentially consistent with a parallel evolution. Again, many of the genes shared between *P. crinitus* and *P. eremicus* are directly related to ribosomal functionality (e.g., *RL36, RS26, RL15*) and also shared with *P. maniculatus*. Determining whether these sweeps are the result of shared new mutations or ancestral variation and whether selection on ribosomal functionality is unique to desert-adapted taxa or more broadly relevant to the genus will require additional tests for selection and expanded taxonomic sampling across the genus *Peromyscus*.

Cellular damage accumulates quickly in desert environments as a consequence of increased thermal and osmotic stress (Lamitina et al. 2006; Burg et al. 2007). In response, expression changes modulate osmoregulation by removing and replacing damaged proteins to prevent cell death (Lamitina et al. 2006); hence, ribosomes, which play a critical role in protein synthesis and degradation, are central to thermoregulatory responses (Porcelli et al. 2015). Although we did not find significantly expanded or contracted gene families within the genus *Peromyscus*, previous investigations of the entire Myodonta clade within Rodentia identified multiple expanded or contracted gene families associated with ribosomes in *P. eremicus* (Tigano et al. 2020). Here, ribosomes appear to be a potential target of parallel evolution in desert-adapted *Peromyscus*, yet this genomic signature is not unique to this genus, nor to desert-adapted species. First, the relative abundance of ribosome-associated genes throughout the genome (>1000 GO annotations pertaining to ribosomes, Bult et al. 2019) may intrinsically increase the representation of this functional group, especially at coarse resolution (10 kbp windows). Second, selection on ribosomal functionality may be commonly experienced across many species adapted to distinct thermal environments (metazoans; Porcelli et al. 2015). Ribosomes are evolutionarily linked to the mitochondrial genomes of animals (Barreto and Burton 2013; Bar-Yaacov et al. 2012) and accelerated mitochondrial evolution in animals has led to compensatory, rapid evolution of ribosomal proteins (Bar-Yaacov et al. 2012; Osada and Akashi 2012; Barreto and Burton 2013). Rapid mitochondrial diversification within *Peromyscus* (Riddle et al. 2000; Bradley et al. 2007; Platt et al. 2015), coincident with the ecological radiation of this genus (Lindsey 2020), suggests that equivalent, recent selection on ribosomal proteins may be a key evolutionary innovation that enabled Peromyscine rodents to successfully and quickly adapt to varied thermal environments. Alternatively, broad selection on ribosomes across all species may also contribute to other, varied aspects of these species biology. Comparisons among additional *Peromyscus* species will be necessary to test these hypotheses in detail.

Evaporative cooling through sweating, panting, or salivating increases water loss and challenges osmoregulatory homeostasis in a hot and dry climate (McKinley et al. 2018). Thermal stress exacerbates dehydration by increasing evaporative water loss and if untreated, can lead to cognitive dysfunction, motor impairment, and eventually death. In consequence, osmoregulatory mechanisms are often under selection in extreme thermal environments (MacManes and Eisen 2014; Marra et al. 2014). Consistent with the importance of osmoregulation in desert species, 4 of the 10 protein-coding genes that experienced a significant selective sweep and were shared between desert specialist species (dataset III) are involved in ion balance (Table 3). Proteins trypsin-2 (*TRY2*) and trimeric intracellular cation channel type-B (*TM38B*) are associated with sweeps in both desert specialists and are involved in calcium ion (Ca^2+^) binding and release, respectively. DNA-directed RNA polymerase III (*RPC1*) has also experienced a significant sweep in both desert specialists and influences magnesium (Mg^2+^) binding. Calcium and magnesium cations are among those essential for osmoregulation (also, Na^+^, K^+^, Cl^−^, HCO_3_^−^; Stockham and Scott 2008) and parallel selection on these genes is consistent with the hypothesis that solute-carrier proteins are essential to maintaining homeostasis in desert-specialized rodents (Marra et al. 2014; Kordonowy and MacManes 2017). Additional genes involved in osmoregulation were identified as experiencing selective sweeps only in *P. crinitus* (Table 2; Supplementary Table S10). Prostatin (*PRSS8*), only found to be under selection in *P. crinitus,* is critically responsible for increasing the activity of epithelial sodium (Na^+^) channels, which mediate sodium reabsorption through the kidneys (Narikiyo et al. 2002). Two more genes associated with Ca^2+^ regulation (*PRSS2* and *TRYP*) and other genes regulating zinc (*KLK4*) and iron (*NCOA4*) were also identified as targets of selective sweeps exclusively in *P. crinitus.*

Genomic scans for selective sweeps based on the SFS are only one way to detect signatures of parallel evolution and these methods can be sensitive to missing data, including low-coverage and small sample sizes; thus, the putative roles of these candidate genes in desert adaptation remains to be explored using additional methods with increased sequencing depth (Booker et al. 2017; Weigand and Leese 2018) and other experimental approaches (e.g., MacManes 2017).

### Metabolic Tuning: Proteins-for-Water or Lipids-for-Torpor?

Hot deserts experience dramatic fluctuations in both food and water availability that challenge species survival (Noy-Meir 1973; Silanikove 1994). Mammals accommodate high temperatures by increasing body temperatures, to a point, and cold temperatures by aerobic thermogenesis or metabolic suppression via the initiation of torpor or hibernation (Levesque et al. 2016). When resources are scarce, metabolism relies exclusively on endogenous nutrients; carbohydrates (e.g., sugars, glucose) are consumed immediately, then lipids, and eventually, proteins. Protein oxidation has a low-energy return relative to lipid catabolism (Bar and Volkoff 2012), but yields 5 times more metabolic water (Jenni and Jenni-Eiermann 1998; Gerson and Guglielmo 2011a, 2011b; McCue et al. 2017). Therefore, in a low-water environment an early shift to protein catabolism during periods of resource limitation may represent an important water source for desert species (e.g., protein-for-water hypothesis; Mosin 1984; Jenni and Jenni-Eiermann 1998; Gerson and Guglielmo 2011a, 2011b). Consistent with this hypothesis, we identified numerous candidate genes that experienced selective sweeps in *P. crinitus* and that are involved in the detection of metabolic stress and shifts in metabolic fuel consumption. For example, the gene eIF-2-alpha kinase GCN2 (*E2AK4*), which is responsible for sensing metabolic stress in mammals and required for adaptation to amino acid starvation, experienced the strongest selective sweep on chromosome 4 in *P. crinitus* (Figure 3; Harding et al. 2003; Baker et al. 2012; Taniuchi et al. 2016). Numerous candidate genes involved in oxidation (Oxidoreductase NAD-binding domain-containing protein 1 [*Oxnad1*]), fat catabolism (Kallikrein-6 [*KLK6*]), protein processing (Kallikrein-13 [*KLK13*]), and proteolysis (Kallikrein [*KLK4*, *KLK13*], Trypsin [*PRSS2, TRYP, TRY2*], Chymotrypsin-like elastase family member 2A [*CELA2A*]) were associated with significantly enriched GO terms in *P. crinitus*. Proteolysis was the most enriched functional group in *P. crinitus* (Figure 4; Supplementary Table S15), potentially supporting the protein-for-water hypothesis.

In contrast to the protein-for-water hypothesis, efficient lipid acquisition and storage may be critical to enabling heat- and drought-induced torpor (Buck et al. 2002; Melvin and Andrews 2009), which allows long duration, low energy survival in desert-adapted species, including *Peromyscus*. Significant weight loss in experimentally dehydrated *P. eremicus* and enhanced thermogenic performance of high-altitude–adapted deer mice have been associated with enhanced lipid metabolism (Cheviron et al. 2012; Kordonowy et al. 2016). At high altitudes, increased lipid oxidation enables aerobic thermogenesis, but in hot deserts, lipids may represent a valuable energy source in a food-scarce environment (e.g., lipids-for-torpor hypothesis). Two additional candidate genes, DCC-interacting protein 13-alpha and -beta (*APPL1*, *APPL2*), experienced significant selective sweeps in *P. crinitus* and are important in glucose regulation, insulin response, and fatty acid oxidation, potentially supporting the lipids-for-torpor hypothesis. Laboratory manipulations of *APPL1* demonstrate protection against high-fat diet-induced cardiomyopathy in rodents (Park et al. 2013) and *APPL2* is responsible for dietary regulation, cold-induced thermogenesis, and cold acclimation (uniprot.org). Together, these genes play a role in both obesity and dietary regulation. Both *APPL* genes are associated with obesity and nonalcoholic fatty liver disease and their sweep signature in *P. crinitus* has relevant connections to biomedical research that remain to be explored (Jiang et al. 2012; Barbieri et al. 2013). Physiological tests will be essential to determine whether desert-adapted deer mice prioritize proteins or fats during periods of resource limitation (e.g., lipids-for-torpor) or extreme dehydration (e.g., protein-for-water hypothesis).

Molecular rewiring of metabolic processes in response to environmental conditions has been documented in a number of species (e.g., mammals, Velotta et al. 2020; birds, Xie et al. 2018; fruit flies, Mallard et al. 2018), but expression changes can also impact species metabolism (Cheviron et al. 2012; Storz and Cheviron 2016). The capacity for rapid molecular adaptation to distinct thermal environments through either transcriptomic regulation or changes to protein-coding genes, combined with thermoregulatory behavioral fine-tuning (e.g., nocturnality, aestivation, food caching, burrowing, dietary shifts), suggests there may be many evolutionary strategies available for small mammals to accommodate increasing temperatures. Anthropogenic change, however, is occurring at a rate that far outpaces the evolutionary timescales on which these adaptations have naturally evolves; Thus, while metabolism and metabolic plasticity represent fundamental phenotypes for anticipating species survival under altered climate scenarios natural selection, alone they may be insufficient for species survival.

### Different Evolutionary Strategies, Same Result

Diverse functional enrichment of the *P. crinitus* genome (Figure 4), spanning metabolic and osmoregulatory functions, in addition to the general functional enrichment of ribosomes, identifies a number of candidate loci worthy of detailed examination. Future comparisons across populations and environments will help determine the influence of these loci and others in thermoregulation, dehydration tolerance, and other adaptive traits. Significant selective sweeps that are not shared among desert specialists, including most of the loci detected here, may still be related to desert adaptation but could also be related to other aspects of this species biology.

There are multiple evolutionary routes to achieve environmental adaptation, most notably through genomic changes in protein-coding genes or transcriptional regulation of gene expression. Lack of evidence for parallel evolution between desert specialists, the proximity of significant selective sweeps to protein-coding genes, diverse functional enrichment of *P. crinitus* relative to *P. eremicus*, and previous gene expression results for *P. eremicus* (Kordonowy and MacManes 2017; MacManes 2017) lead us to hypothesize alternative evolutionary strategies for each desert specialist, each shaped by their independent demographic histories: *P. crinitus* primarily through changes in protein-coding genes and *P. eremicus* primarily through transcriptional regulation. Evidence of many significant sweep sites in the *P. eremicus* genome, located more distant from protein-coding genes, and with functional enrichment restricted to ribosomes, suggests that adaptation in this species may be driven more by selection on regulatory or noncoding regions of the genome that impact gene expression, a hypothesis that is consistent with transcriptomic investigations in this species (MacManes and Eisen 2014; Kordonowy and MacManes 2017) and other *Peromyscus* and rodents (Cheviron et al. 2012; Marra et al. 2014; Storz and Cheviron 2016). Without equivalent gene expression data for *P. crinitus*, we cannot eliminate a similarly important role for transcriptional regulation and look forward to testing this hypothesis in greater detail with RNAseq data. Transcriptional regulation is a particularly useful mechanism for environmental acclimation, as these changes are more transient relative to genomic changes and can enhance phenotypic flexibility (Garrett and Rosenthal 2012; Rieder et al. 2015; Liscovitch-Brauer et al. 2017). Reduced variation is expected near selective sweeps and can encompass tens to thousands of adjacent nucleotides depending on recombination and the strength of selection (Fay and Wu 2000; Carlson et al. 2005), yet counter to expectations, Tajima’s *D* and nucleotide diversity for regions flanking putative selective sweeps were significantly higher than the global average for most comparisons (Supplementary Table S20). The same observation, of elevated Tajima’s *D* and nucleotide diversity surrounding selective sweeps, was also made in *P. eremicus* (Tigano et al. 2020). This counterintuitive pattern also holds across different window sizes (1 kbp, 10 kbp) and warrants further investigation.

Placing the results of selective sweep analyses within an evolutionary framework is important to interpreting adaptive evolutionary responses, including the order and sequence of such events. The genus *Peromyscus* originated approximately 8 Mya, followed by a massive radiation around 5–6 Mya, which probably led to the divergence of a monophyletic desert-adapted clade. The ancestral state of this clade remains unknown, but species therein likely colonized arid environments through either a single or multiple invasions, followed by additional interspecific divergence (Platt et al. 2015). The expansion of North American deserts following the conclusion of the last glacial maximum (~11 Kya; Pavlik et al. 2008) constrains the adaptive timescales of contemporary desert-adapted species. The consistently low and stable effective population size of *P. eremicus* over deep time scales suggests that this species has historically harbored less standing genetic variation, despite equivalent contemporary levels diversity relative to *P. crinitus* (Figure 2). Evolution and adaptation are more impacted by genetic drift in species with small population sizes (Allendorf 1986; Masel 2011). *Peromyscus crinitus*, which historically has a much larger effective population size, is expected to have a broader pool of variation for selection to act upon over longer evolutionary timescales, consistent with the higher diversity of genes and enriched GO terms observed as experiencing selective sweeps in *P. crinitus*. From a demographic perspective, regulation of gene expression would be a more expedient means of environmental adaptation available to *P. eremicus* (Allendorf 1986; Barton 2010; Neme and Tautz 2016; Mallard et al. 2018), whereas the maintenance of higher effective population size of *P. crinitus* would enable more rapid evolution of protein-coding sequences due to the reduced impact of genetic drift, larger pool of standing genetic variation, and potentially, gene flow. *Peromyscus crinitus* experienced a historical demographic bottleneck prior to the formation of North American deserts; Nevertheless, the recovered effective population size of this species is much larger than *P. eremicus* and consistent with low levels of detected admixture in *P. crinitus* (Supplementary Figure S7, Supplementary Table S8). Consistently, negative Tajima’s *D* values can also indicate population expansion following a bottleneck. Evidence of a historical bottleneck is further reinforced by moderate to high levels of nucleotide diversity in *P. crinitus*. Repeated growth and contraction of rivers in the American Southwest during Pleistocene glacial-interglacial cycles (0.7–0.01 Mya; Muhs et al. 2003; Van Dam and Matzke 2016) would have provided iterative opportunities for connectivity between incompletely isolated *Peromyscus* species and historical hybridization between *P. crinitus* and one or more Peromyscine species, likely unsampled here, may have contributed to adaptation in *P. crinitus*. Low-coverage whole-genome resequencing is optimal for population genomics investigations (O’Rawe et al. 2015; da Fonseca et al. 2016), but limits detailed analyses of historical introgression and we look forward to testing this hypothesis with expanded population sampling and increased sequencing depth. Finally, linkage disequilibrium decays faster in larger populations, where recombination is higher, therefore the shorter distance between significant sweep sites and the nearest coding gene in *P. crinitus* could also be a consequence of the larger historical population sizes of this species relative to *P. eremicus.* However, the evolutionary scales of *PSMC* and *Sweepfinder2* do not overlap, as *PSMC* characterizes historical demography beyond 10 kya, whereas selective sweeps have occurred recently. Overall, incorporating an evolutionary perspective into the interpretation of selection patterns has important implications for understanding species responses to changing climate, as historical demography and gene flow, in addition to selection, shape genetic diversity over evolutionary timescales.

## Conclusion

Contrasting patterns of selective sweeps and evolutionary histories between different species experiencing similar environmental pressures can provide powerful insight into the adaptive potential of species. We used comparative and population genomic analyses of 3 *Peromyscus* species to identify candidate loci that may underlie adaptations to desert environments. Candidate loci identified in *P. crinitus* serve to inform future investigations focused on predicting potential for adaptation and identifying the causes of warming-related population declines (Cahill et al. 2013). The identification of numerous targets of selection within *P. crinitus* highlights multiple molecular mechanisms (metabolic switching, osmoregulatory tuning) associated with physiological responses to deserts that warrant further investigation. Our approach demonstrates the importance of placing genomic selection analyses into an evolutionary framework to anticipate evolutionary responses to change.

## Supplementary Material

esab009_suppl_Supplementary_Materials

## Data Availability

The draft assembly data are housed on the European Nucleotide Archive (ENA) under project ID PRJEB33592. The Hi-C data are available on SRA (SRX7041777, SRX7041776, SRX7041773) under the DNA Zoo project accession PRJNA512907. The *P. crinitus* genome assembly is available at https://www.dnazoo.org/assemblies/Peromyscus_crinitus. Whole-genome resequencing data for *P. crinitus* are available on ENA under project ID PRJEB35488. Custom python scripts and other bash scripts used in analysis are available at https://github.com/jpcolella/Peromyscus_crinitus.
