# Supplementary material for: Limited Evidence for Parallel Evolution Among Desert-Adapted Peromyscus Deer Mice
Source: J Hered. 2021 Mar 4;112(3):286–302. doi: 10.1093/jhered/esab009 (PMC8141686; doi:10.1093/jhered/esab009)
Supplement: esab009_suppl_Supplementary_Materials [file esab009_suppl_supplementary_materials.docx]

**Limited evidence for parallel evolution among desert-adapted *Peromyscus* deer mice**

**Supporting Information**

To ensure selection analyses were conducted across unrelated individuals, we ran *ngsRelate* (<https://github.com/ANGSD/NgsRelate>) within each *Peromyscus* species and for each sampled *P. eremicus* population: Philip L. Boyd Deep Canyon Research Center and Motte Rimrock Reserve (Tigano et al. 2020). To examine population structure, we ran a Principle Component Analysis (PCA; *ngsTools,* Fumagalli et al. 2014) on the genotype posterior probabilities called across all species. PCA’s are sensitive to missing data (Anderson et al. 2010), and therefore not necessarily appropriate for low-coverage genomic data and the PCA was run both with and without missing data. As a more accurate method for low-coverage data, we used multidimensional scaling (MDS) in *ANGSD* as a means of dimensionality reduction to visualize population differentiation.

**Supporting Results**

No *P. crinitus* or *P. maniculatus* individuals were closely related. Two pairs of *P. eremicus* individuals were identified as at least first-cousins (> 0.75) and one member of each pair was removed from downstream selection analyses. Including missing data, the first two PCA axes explained 24.8% and 15.6% of the variation, respectively, with multiple lower coverage *P. crinitus* individuals displaced towards the center of the PCA. Excluding missing data, the three species were well sorted with PC1 accounting for 19% of the total variation and PC2, 11.3% (Fig. S6).

For *P. crinitus* eight unique genes were identified within the 20 most significant sweeps, in order of most to least significance: *Biotinidase, Washc4* (WASH complex subunit 4)*, APPL2/DP13B* (DCC-interacting protein 13-beta)*, Aldh1L2* (mitochondrial 10-formyltetrahydrofolate dehydrogenase)*, COLQ* (acetylcholinesterase collagenic tail peptide)*, EAF1* (ELL-associated factor 1)*, ANKRD28* (serine/threonine-protein phosphatase 6 regulator ankyrin repeat subunit A)*, and Aldh1A1* (retinal dehydrogenase 1)*.* Seven unique genes were identified among the 20 most significant sweeps in *P. eremicus*, including: *GDF10* (growth/differentiation factor 10)*, ZNF331* (zinc finger protein 331)*, ZN304* (zinc finger protein 304)*, Ptnp20* (Tyrosine-protein phosphatase non-receptor type 20)*, LRC18* (Leucine-rich repeat-containing protein 18)*, Aldh1I1* (Cystolic 10-formyltetrahydrofolate dehydrogenase)*,* and *Vmn2r26* (vomeronasal type-2 receptor 26), none of which exactly overlap with the other desert specialist. However, the two species do share strong signatures of selective sweeps surrounding *Aldh* genes, which are involved in detoxification. Finally, for *P. maniculatus* only three unique genes were identified proximal to the 20 most significant sweep sites, including: *FCF1* (rRNA-processing protein FCF1 homolog)*, Crb2* (protein crumbs homolog 2)*,* and *DENND1A* (DENN domain-containing protein 1A), also not shared with the other *Peromyscus* species.

Examination of the single closest gene (dataset I) to each significant sweep site identified 88 unique genes and 14 enriched GO (gene ontology0 terms (1 Biological Processes [BP], 7 Molecular Function [MF], 6 Cellular Component[CC]) for *P. crinitus,* 127 unique genes and eight enriched GO terms (1 BP, 1 MF, 6 CC) for *P. eremicus*, and 31 unique genes and eight enriched GO terms for *P. maniculatus*. Enriched functionality in *P. crinitus* pertained to metabolism (e.g., ‘protein metabolic process’, ‘organonitrogen compound metabolic process’, ‘peptide metabolic process’) and ribosomes. For dataset I, one gene and zero enriched GO terms were shared between the two desert adapted species, demonstrating no support for parallel evolution.

Examination of the four nearest genes to each sweep site (dataset III: one upstream, one downstream gene on each strand) identified 171 unique genes and 47 enriched GO terms (15 BP, 13 MF, 19 CC) for *P. crinitus* and 335 unique genes and 25 enriched GO terms (15 BP, 4 MF, 6 CC) for *P. eremicus*. Functionality of *P. crinitus* enriched GO terms ranged from metabolic processes, hydrolase and peptidase activity, to ribosomal functions. For *P. eremicus*, dataset III again centered on ribosomal functionality with the addition of multiple GO terms pertaining to ‘RNA binding’ and ‘translation’. For dataset III, 10 genes and 7 enriched GO terms were shared between *P. crinitus* and *P. eremicus*. Datasets II and III show substantial GO term overlap between the two desert-adapted species with 40-50% of *P. eremicus* enriched GO terms shared with *P. crinitus.* Fisher’s exact test was significant (p = 0.001) indicating there is a relationship between genes identified under selection between the two species only for dataset III.

We identified 21 anomalous coverage values (0 or >1000) surrounding sweep sites in *P. eremicus.* We hypothesize that these values be explained by population structure; sequence data from multiple populations were included in the *Sweepfinder2* analysis, but only the high-quality reference assembly was used to calculate local coverage surrounding sweep sites. Alternatively, this pattern could be explained by base compositional bias, misassembly, or differences in the mapping algorithms used by *ANGSD/Sweepfinder2* versus *Supernova/Juicebox.* While the underlying cause of these outliers is unknown, they do not significantly impact our results. Only one gene (*RS2*) with anomalous coverage results in *P. eremicus* overlapped with genes identified as experiencing a selective sweep in the other two species. RS2 is one of many genes identified as under selection that also pertains to ribosomal functionality.

**Supporting References**

Anderson, C., Pettersson, F., Clarke, G., Cardon, L., Morris, A., & Zondervan, K. (2010). Data quality control in genetic case-control association studies. *Nat. Protoc., 5*(9), 1564–1573.

DeGiorgio, M., Huber, C., Hubisz, M., Hellmann, I., & Nielsen, R. (2016). SweepFinder2: increased sensitivity, robustness and flexibility. *Bioinformatics, 32*(12), 1895–1897.

Fumagalli, M., Vieira, F., Linderoth, T., & Nielsen, R. (2014). ngsTools: methods for population genetics analyses from next-generation sequencing data. *Bioinformatics, 30*(10), 1486–1487.

Tigano, A., Colella, J., & MacManes, M. (2020). Comparative and population genomics approaches reveal the basis of adaptation to deserts in a small rodent. *Molecular Ecology, 29*, 1300–1314.

**Supporting Materials**

**Table S1.** Individuals identifiers (ID), species, collection locality (Coll. Loc.), coverage (Gb), and collection year for population-level sampling. Deep Canyon Desert Research Center (DCDRC), Motte Rimrock Reserve (Motte), and Elliot-Chaparral Reserve (Elliot). All reserve sites belong to the University of California natural Reserve System, located in southern California.

| **Species** | **Sample ID** | **Coll. Loc.** | **Coverage** | **Sample Year** |
| --- | --- | --- | --- | --- |
| *crinitus* | pecr303 | DCDRC | 14.8 | 2009 |
|  | pecr304 | DCDRC | 12.4 | 2009 |
|  | pecr312 | DCDRC | 9.6 | 2009 |
|  | pecr315 | DCDRC | 8.8 | 2009 |
|  | pecr306 | DCDRC | 8.1 | 2009 |
|  | pecr313 | DCDRC | 8.1 | 2009 |
|  | pecr320 | DCDRC | 8.0 | 2009 |
|  | pecr307B | DCDRC | 5.3 | 2009 |
|  | pecr318 | DCDRC | 1.9 | 2009 |
| *maniculatus* | pema343W | Elliott | 16.2 | 2009 |
|  | pema331 | Motte | 15.3 | 2009 |
|  | pema342 | Elliott | 11.4 | 2009 |
|  | pema328 | Motte | 9.9 | 2009 |
|  | pema337 | Motte | 8.4 | 2009 |
| *eremicus* | peer12 | DCDRC | 3.9 | 2009 |
|  | peer339 | Motte | 17.1 | 2009 |
|  | peer338 | Motte | 14.8 | 2009 |
|  | peer138 | DCDRC | 13.6 | 2018 |
|  | peer326 | Motte | 13.3 | 2009 |
|  | peer136 | DCDRC | 12.7 | 2018 |
|  | peer15 | DCDRC | 12.2 | 2009 |
|  | peer336 | Motte | 11.9 | 2009 |
|  | peer335 | Motte | 11.8 | 2009 |
|  | peer139 | DCDRC | 10.4 | 2018 |
|  | peer134 | DCDRC | 10.2 | 2018 |
|  | peer305 | DCDRC | 10.0 | 2018 |
|  | peer92 | DCDRC | 9.6 | 2009 |
|  | peer334 | Motte | 9.2 | 2009 |
|  | peer98 | DCDRC | 9.0 | 2009 |
|  | peer91 | DCDRC | 8.5 | 2009 |
|  | peer75 | DCDRC | 8.1 | 2018 |
|  | peer95 | DCDRC | 8.0 | 2009 |
|  | peer135 | DCDRC | 7.6 | 2018 |
|  | peer16 | DCDRC | 7.4 | 2009 |
|  | peer333 | Motte | 7.2 | 2009 |
|  | peer99 | DCDRC | 5.6 | 2009 |
|  | peer93 | DCDRC | 5.1 | 2009 |
|  | peer330 | Motte | 3.8 | 2009 |
|  | peer17 | DCDRC | 3.0 | 2009 |
|  | peer144 | DCDRC | 1.9 | 2018 |

**Table S2.** *A priori* candidate loci including all annotated aquaporins, cytochrome P540 genes, and *SLC8a1* and their location in the *P. crinitus* reference genomes (start and stop positions in basepairs [bp]) and chromosome (Chr).

| Candidate | Protein name | Start | Stop | Chr |
| --- | --- | --- | --- | --- |
| *Aqp1* | Aquaporin-1 | 70132971 | 70141922 | 3 |
| *Aqp2* | Aquaporin-2 | 3478544 | 3482779 | 20 |
| *Aqp3* | Aquaporin-3 | 60047430 | 60052839 | 2 |
| *Aqp4* | Aquaporin-4 | 6061783 | 6073875 | 19 |
| *Aqp7* | Aquaporin-7 | 59974693 | 59988302 | 2 |
| *Aqp8* | Aquaporin-8 | 74407923 | 74414209 | 1 |
| *Aqp9* | Aquaporin-9 | 49118583 | 49160335 | 7 |
| *Aqp10* | Aquaporin-10 | 64351601 | 64353917 | 6 |
| *Aqp11* | Aquaporin-11 | 97812149 | 97826943 | 1 |
| *Aqp12* | Aquaporin-12 | 8704857 | 8709981 | 13 |
| *SLC8a1* | *Sodium/calcium exchanger 1* | 30182624 | 30240551 | 22 |
|  |  | 30462882 | 30464651 | 22 |
| *Cyp46a1* | Cytochrome P450 family 46 subfamily A member 1 | 72801879 | 72830617 | 14 |
| *Cyp4a10* | Cytochrome P450 family 4 subfamily A member 10 | 124674282 | 124689006 | 2 |
| *Cyp4a12a* | Cytochrome P450 family 4 subfamily A member 12a | 124567023 | 124584342 | 2 |
| *Cyp4a14* | Cytochrome P450 family 4 subfamily A member 14 | 124636158 | 124647064 | 2 |
| *Cyp4b1* | Cytochrome P450 family 4 subfamily B member 1 | 124690725 | 124708680 | 2 |
| *Cyp4f1* | Cytochrome P450 family 4 subfamily F member 1 | 45261362 | 45299884 | 22 |
| *Cyp4f14* | Cytochrome P450 family 4 subfamily F member 14 | 44948658 | 44962571 | 22 |
| *Cyp4f3* | Cytochrome P450 family 4 subfamily F member 3 | 56917804 | 56939832 | 17 |
| *Cyp4f4* | Cytochrome P450 family 4 subfamily F member 4 | 45190547 | 45207847 | 22 |
|  |  | 45232034 | 45243762 | 22 |
| *Cyp4f5* | Cytochrome P450 family 4 subfamily F member 5 | 45324459 | 45334720 | 22 |
| *Cyp4f6* | Cytochrome P450 family 4 subfamily F member 6 | 44915197 | 44939533 | 22 |
| *Cyp4v2* | Cytochrome P450 family 46 subfamily V member 2 | 34947722 | 34973724 | 17 |
| *Cyp4x1* | Cytochrome P450 family 4 subfamily X member 1 | 124475444 | 124502513 | 2 |

**Table S3.** Percent complete BUSCOs (%), source, and GenBank ID for all species used in *CAFE* and species tree analysis.

| Species | % | Source | ID |
| --- | --- | --- | --- |
| *Peromyscus crinitus* | 87.5 | This study | This study |
| *Peromyscus polionotus* | 95.4 | Howard Hughes Medical Institute (HHMI) | GCA_003704135.2 |
| *Peromyscus maniculatus* | 95.3 | HHMI | GCA_000500345.1 |
| *Mus musculus* | 95.2 | Genome Reference Consortium | GCA_000001635.8 |
| *Microtus ochrogaster* | 95.2 | Broad Institute | GCA_000317375.1 |
| *Peromyscus leucopus* | 94.5 | Long et al. 2019 | GCA_004664715.1 |
| *Peromyscus eremicus* | 92.9 | Tigano et al. 2020 | GCA_902702925.1 |
| *Peromyscus nasutus* | 89.3 | DNAzoo | Peromyscus_nasutus_10x_v1_HiC |
| *Sigmodon hispidus* | 86.6 | Broad Institute | GCA_004025045.1 |
| *Neotoma lepida* | 78 | Campbell et al. 2016 | LZPO01000000 |
| *Peromyscus attwateri* | 66.4 | Colella et al. 2020 | ERZ1029326 |
| *Peromyscus nudipes* | 62.2 | Colella et al. 2020 | ERZ1029275 |
| *Peromyscus aztecus* | 61.7 | Colella et al. 2020 | ERZ1029324 |
| *Peromyscus melanophrys* | 55.1 | Colella et al. 2020 | ERZ1029325 |
|  |  |  |  |

**Figure S4.** Visualization of chromosome-level synteny between *P. crinitus* (X axis) and *P. maniculatus* (Y axis) genomes based on *MashMap.*

**
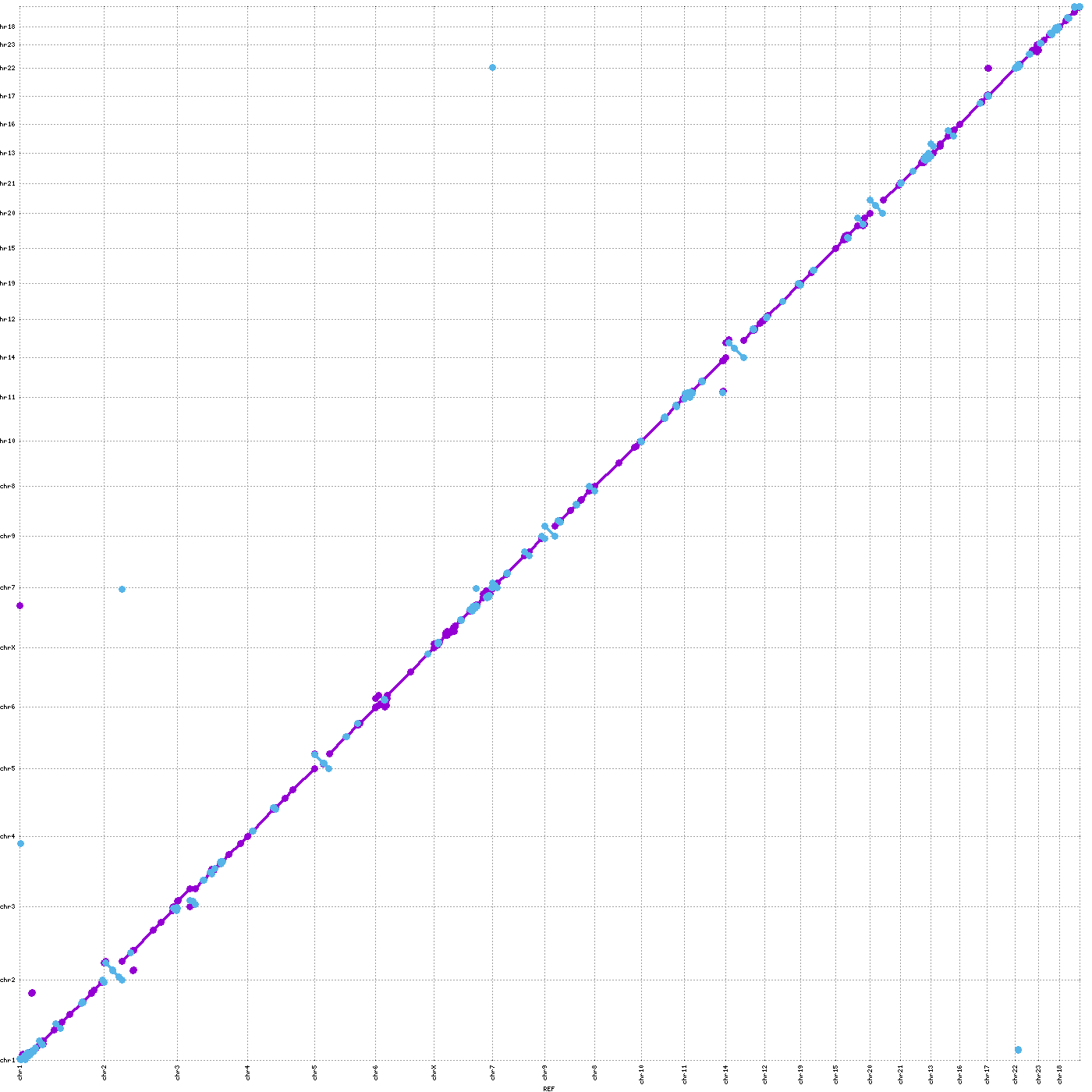
**

**Table S5.** Results from *RepeatMasker.* Values for additional *Peromyscus* species are available in Tigano et al. (2020, Supplementary Table 2).

| Species | SINEs | LINEs | LTRs | DNA Elements | Unclass. | Satellites | Simple repeats | Low. Compl. | Total Masked |
| --- | --- | --- | --- | --- | --- | --- | --- | --- | --- |
| *P. crinitus* | 9.6 | 11.83 | 11.59 | 1.52 | 0.49 | 0.09 | 2.03 | 0.29 | 37.46 |
| *P. eremicus* | 9.22 | 10.54 | 10.51 | 1.45 | 0.46 | 0.08 | 2.07 | 0.28 | 34.68 |
| *P. maniculatus* | 9.04 | 10.44 | 10.33 | 1.38 | 0.47 | 0.07 | 2.11 | 0.28 | 34.19 |

**Figure S6.** (Left) Multidimensional scaling plot (left) of all three *Peromyscus* species; (center) PCA plot excluding missing data; (right) PCA plot including missing data. *P. crinitus* in black, *P. eremicus* in light gray, and *P. maniculatus* in dark grey.

**
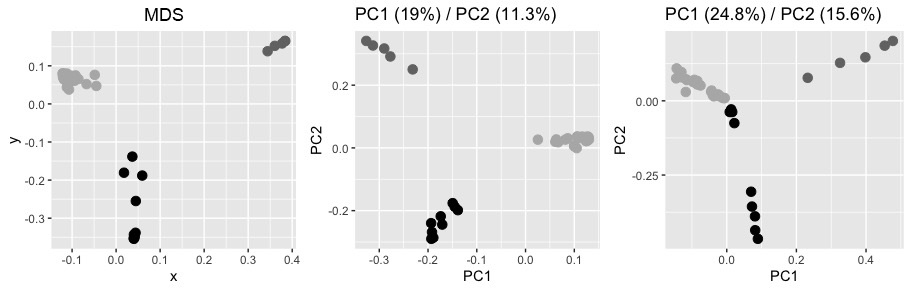
**

**Figure S7.** Two, three, and four population (K) models fit to 40 *Peromyscus* individuals, each represented by a vertical bar, with ancestry proportions assigned to groups of different colors. K of one (not shown) had the highest maximum likelihood score, but K of three accurately identifies the three different species: *P. eremicus* (blue), *P. crinitus* (red), and *P. maniculatus* (yellow). Collection localities within *P. eremicus*: MOT = Motte Rimrock Reserve (all collected in 2009); DC = Deep Canyon Desert Research Center Reserve, collected in 2018 (18) or 2009 (09).


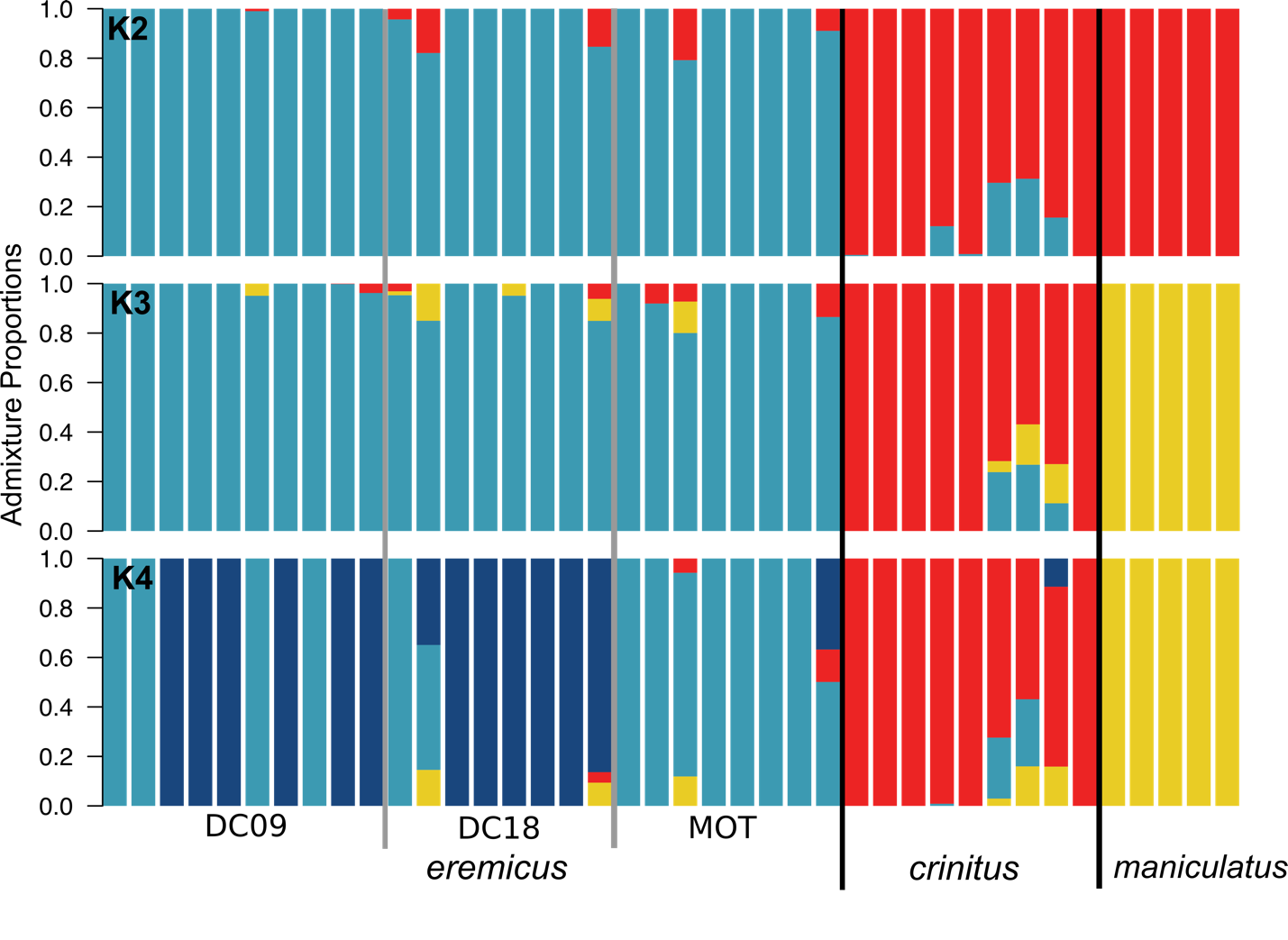


**Table S8** Maximum likelihood estimates for K=1 through K=15 for all species (Global) combined and each species separately. Highest likelihood denoted in ***bold italics***.

| K | Global | *P. crinitus* | *P. eremicus* | *P. maniculatus* |
| --- | --- | --- | --- | --- |
| 1 | ***-5909871506*** | ***-148221048.6*** | ***-185879999.2*** | ***-88731308.39*** |
| 2 | -4655817366 | -136248657.3 | -179173995.1 | -72155709.39 |
| 3 | -3902013433 | -125362815.3 | -174376420.6 | -59870525.96 |
| 4 | -3848194753 | -114546875.7 | -168936527.5 | -47582293.11 |
| 5 | -3804562469 | -104599522.8 | -164921362.5 | -35468773.4 |
| 6 | -3751439723 | -98822916.35 | -160819394.9 | - |
| 7 | -3729130719 | -94900084.22 | -157049112.4 | - |
| 8 | -3682543099 | -90452152.43 | -153351474.5 | - |
| 9 | -3652484684 | -85201348.65 | -149583609.6 | - |
| 10 | -3630337583 | - | -145500236.6 | - |
| 11 | -3611896800 | - | -141421674.2 | - |
| 12 | -3563534421 | - | -138584857.6 | - |
| 13 | -3547932860 | - | -136557781.2 | - |
| 14 | -3524022116 | - | -134241460.4 | - |
| 15 | -3487398904 | - | -129294932.7 | - |

**Figure S9.** Histogram of raw coverage grouped by admixed and non-admixed samples, as identified under a K=3 model in *ngsAdmix.*


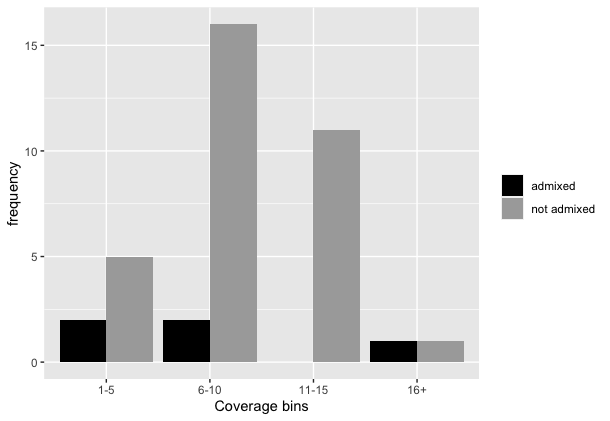


**Table S10.** Positions (Sweep site) identified as having experienced a recent selective sweep in *P. crinitus* and the nearest gene (Gene id) and distance (in bp) to that gene. Relative location (loc.) indicates whether the site is located inside of a gene or if the nearest gene is upstream (U) or downstream (D) on the sense (F) or antisense (R) strand. Gene direction (dir.) is indicated by a + (forward), - (reverse). The boundaries of the 10 kbp window (5 kbp on either side of the sweep site) are indicated by the start and stop columns, and local coverage (cov.) is reported for each 10 kbp window.

| Chr | Sweep site | Relative loc. | Gene dir | Gene id | Start | Stop | Distance | Local cov. |
| --- | --- | --- | --- | --- | --- | --- | --- | --- |
| 6 | 66203934 | gene | - | NEST_MOUSE | 66203312 | 66204100 | 0 | 30 |
|  |  |  |  |  | 66203315 | 66204100 | 0 |  |
| 9 | 23303147 | gene | + | DEN6A_HUMAN | 23303022 | 23303252 | 0 | 30 |
|  |  |  |  |  | 23303022 | 23303249 | 0 |  |
| 9 | 23323150 | gene | + | DEN6A_HUMAN | 23323150 | 23323287 | 0 | 38 |
| 9 | 43305800 | gene | + | NYNRI_MOUSE | 43304238 | 43307081 | 0 | 42 |
|  |  |  |  |  | 43304243 | 43307081 | 0 |  |
| 9 | 22243007 | gene | - | PARG_BOVIN | 22242615 | 22243577 | 0 | 28 |
|  |  | gene | - | PARG_RAT | 22242616 | 22243575 | 0 |  |
|  |  | gene | - | PARG_BOVIN | 22242616 | 22243584 | 0 |  |
| 9 | 22283012 | gene | - | NCOA4_HUMAN | 22282962 | 22283072 | 0 | 37 |
|  |  |  |  |  | 22282962 | 22283070 | 0 |  |
| 9 | 22303015 | gene | - | OXND1_MOUSE | 22302824 | 22303021 | 0 | 56 |
| 23 | 28065942 | gene | + | TECT1_HUMAN | 28065831 | 28065995 | 0 | 65 |
|  |  | gene | + | TECT1_MOUSE | 28065852 | 28065995 | 0 |  |
|  |  |  |  |  | 28065855 | 28065995 | 0 |  |
| 3 | 52113514 | gene | + | TRY2_HUMAN | 52113385 | 52113531 | 0 | 29 |
|  |  |  |  |  | 52113382 | 52113531 | 0 |  |
|  |  |  | + | KLK13_HUMAN | 52113382 | 52113528 | 0 |  |
|  |  |  | + | PRSS8_HUMAN | 52113382 | 52113567 | 0 |  |
|  |  |  | + | KLK4_HUMAN | 52113385 | 52113522 | 0 |  |
|  |  |  |  |  | 52113380 | 52113522 | 0 |  |
|  |  |  | + | PRTN3_HUMAN | 52113331 | 52113522 | 0 |  |
|  |  |  | + | KLK14_HUMAN | 52113385 | 52113528 | 0 |  |
|  |  |  | + | TRYP_PIG | 52113355 | 52113531 | 0 |  |
|  |  |  | + | KLK6_HUMAN | 52113382 | 52113522 | 0 |  |
|  |  |  | + | CEL2A_HUMAN | 52113370 | 52113531 | 0 |  |
| 4 | 57673659 | gene | - | E2AK4_HUMAN | 57673646 | 57673816 | 0 | 32 |
|  |  |  |  |  | 57673646 | 57673814 | 0 |  |
| 18 | 450151 | gene | - | DP13B_HUMAN | 450084 | 450191 | 0 | 41 |
|  |  |  |  |  | 450103 | 450191 | 0 |  |
|  |  |  | - | DP13A_HUMAN | 450114 | 450191 | 0 |  |
| 18 | 460153 | gene | - | DP13B_HUMAN | 460136 | 460291 | 0 | 36 |
|  |  |  |  |  | 460142 | 460288 | 0 |  |
|  |  |  | - | DP13A_HUMAN | 460115 | 460291 | 0 |  |
|  |  |  |  |  | 460142 | 460389 | 0 |  |
| 2 | 70823185 | F.U. | + | TAL2_HUMAN | 70821972 | 70822280 | 905 | 38 |
| 3 | 52123514 | R.U. | - | TRY2_CANLF | 52129452 | 52129601 | 5938 | 32 |
| 4 | 17961209 | R.D. | - | DHX35_PONAB | 17892273 | 17892371 | 68838 | 24 |
| 4 | 20351357 | F.D. | + | TGM2_HUMAN | 20355519 | 20355708 | 4162 | 26 |
| 4 | 20361357 | F.U. | + | TGM5_MOUSE | 20359994 | 20360299 | 1058 | 28 |
| 4 | 57653657 | R.U. | - | E2AK4_HUMAN | 57654063 | 57654134 | 406 | 30 |
| 4 | 57663658 | R.U. | - | E2AK4_HUMAN | 57664472 | 57664636 | 814 | 33 |
| 4 | 57683659 | R.U. | - | E2AK4_HUMAN | 57683915 | 57683968 | 256 | 31 |
| 4 | 57693660 | R.U. | - | E2AK4_HUMAN | 57694478 | 57694567 | 818 | 32 |
| 4 | 57813667 | R.U. | - | E2AK4_HUMAN | 57854804 | 57854932 | 41137 | 26 |
| 4 | 57833669 | R.U. | - | E2AK4_HUMAN | 57854804 | 57854932 | 21135 | 30 |
| 4 | 57843669 | R.U. | - | E2AK4_HUMAN | 57854804 | 57854932 | 11135 | 28 |
| 5 | 30763569 | R.U. | - | FOXC1_HUMAN | 30817627 | 30817794 | 54058 | 27 |
| 5 | 30773570 | R.U. | - | FOXC1_HUMAN | 30817627 | 30817794 | 44057 | 27 |
| 5 | 93600635 | R.U. | - | MON1B_MOUSE | 94039757 | 94039960 | 439122 | 40 |
| 6 | 66213935 | R.U. | - | NEST_MOUSE | 66220840 | 66220887 | 6905 | 36 |
| 6 | 66223936 | R.U. | - | RABP2_RAT | 66224985 | 66225056 | 1049 | 37 |
| 6 | 66263938 | R.U. | - | RM24_MOUSE | 66268874 | 66269107 | 4936 | 32 |
| 6 | 66273939 | R.U. | - | HDGF_MOUSE | 66276050 | 66276145 | 2111 | 28 |
| 6 | 66283939 | R.D. | - | SH22A_HUMAN | 66276050 | 66276145 | 7794 | 34 |
| 6 | 66293940 | F.D. | + | PRCC_HUMAN | 66297519 | 66297980 | 3579 | 32 |
| 6 | 66303940 | F.U. | + | PRCC_HUMAN | 66297519 | 66297980 | 5960 | 35 |
| 7 | 30952967 | F.D. | + | RL13A_BOVIN | 30998679 | 30998915 | 45712 | 34 |
| 7 | 30962967 | F.D. | + | RL13A_BOVIN | 30998679 | 30998915 | 35712 | 34 |
| 9 | 4850700 | R.U. | - | CAPS1_RAT | 4851908 | 4852075 | 1208 | 27 |
| 9 | 4860701 | R.U. | - | CAPS1_MOUSE | 4863531 | 4863680 | 2830 | 32 |
| 9 | 4870703 | R.U. | - | CAPS1_MOUSE | 4870954 | 4871064 | 251 | 28 |
| 9 | 4880704 | R.U. | - | CAPS1_MOUSE | 4884580 | 4884597 | 3876 | 28 |
| 9 | 4890705 | R.D. | - | CAPS1_RAT | 4890104 | 4890208 | 497 | 31 |
| 9 | 4900706 | R.U. | - | CAPS1_RAT | 4902823 | 4903011 | 2117 | 26 |
| 9 | 4910708 | R.D. | - | CAPS1_RAT | 4906184 | 4906338 | 4370 | 33 |
| 9 | 4920709 | R.D. | - | CAPS1_RAT | 4919527 | 4919739 | 970 | 34 |
| 9 | 4930710 | R.U. | - | CAPS2_MOUSE | 4933662 | 4933733 | 2952 | 34 |
| 9 | 4940712 | R.D. | - | CAPS2_MOUSE | 4933668 | 4933734 | 6978 | 389 |
| 9 | 4950713 | R.U. | - | CAPS1_RAT | 4951210 | 4951362 | 497 | 27 |
| 9 | 4960714 | R.D. | - | DDX52_HUMAN | 4956817 | 4956939 | 3775 | 27 |
| 9 | 4970716 | R.D. | - | DDX52_HUMAN | 4956817 | 4956939 | 13777 | 30 |
| 9 | 5230750 | R.D. | - | DDX52_HUMAN | 4956817 | 4956939 | 273811 | 30 |
| 9 | 22172997 | R.U. | - | PARG_BOVIN | 22180265 | 22180414 | 7268 | 58 |
| 9 | 22203001 | R.U. | - | PARG_BOVIN | 22203375 | 22203536 | 374 | 32 |
| 9 | 22213003 | R.U. | - | PARG_BOVIN | 22214073 | 22214357 | 1070 | 32 |
| 9 | 22223004 | R.D. | - | PARG_BOVIN | 22214452 | 22214514 | 8490 | 28 |
| 9 | 22233005 | R.U. | - | GSTA4_BOVIN | 22233444 | 22233521 | 439 | 31 |
| 9 | 22253008 | R.D. | - | NCOA4_HUMAN | 22252097 | 22252315 | 693 | 30 |
| 9 | 22263009 | F.U. | + | TIM23_HUMAN | 22262747 | 22262805 | 204 | 28 |
| 9 | 22273011 | F.D. | + | TIM23_HUMAN | 22275083 | 22275196 | 2072 | 32 |
| 9 | 22293013 | R.D. | - | OXND1_MOUSE | 22284985 | 22285128 | 7885 | 49 |
| 9 | 22313016 | R.U. | - | OXND1_MOUSE | 22314923 | 22315064 | 1907 | 51 |
| 9 | 22683065 | R.U. | - | OST4_HUMAN | 22713491 | 22713805 | 30426 | 45 |
| 9 | 22693066 | R.U. | - | OST4_HUMAN | 22713491 | 22713805 | 20425 | 36 |
| 9 | 22703068 | R.U. | - | OST4_HUMAN | 22713491 | 22713805 | 10423 | 58 |
| 9 | 22713069 | R.U. | - | OST4_HUMAN | 22713491 | 22713805 | 422 | 35 |
| 9 | 22723070 | R.D. | - | VNN3_HUMAN | 22713822 | 22713878 | 9192 | 32 |
| 9 | 22733072 | F.D. | + | ANR28_HUMAN | 22733756 | 22733839 | 684 | 27 |
| 9 | 22743073 | F.D. | + | ANR44_HUMAN | 22748544 | 22748702 | 5471 | 29 |
| 9 | 22753074 | F.U. | + | ANR28_HUMAN | 22748619 | 22748702 | 4372 | 30 |
| 9 | 22763076 | F.U. | + | ANR28_HUMAN | 22759491 | 22759577 | 3499 | 32 |
| 9 | 22773077 | F.U. | + | ANR52_HUMAN | 22772758 | 22772952 | 125 | 30 |
| 9 | 22783078 | F.D. | + | ANR52_HUMAN | 22783508 | 22783609 | 430 | 30 |
| 9 | 22793080 | F.U. | + | ANR28_HUMAN | 22792394 | 22792549 | 531 | 28 |
| 9 | 22803081 | F.U. | + | ANR28_HUMAN | 22802683 | 22802884 | 197 | 30 |
| 9 | 22813082 | F.D. | + | ANR28_HUMAN | 22813277 | 22813368 | 195 | 32 |
| 9 | 22823084 | F.U. | + | ANR28_HUMAN | 22819579 | 22819863 | 3221 | 30 |
| 9 | 22833085 | R.U. | - | RS10L_HUMAN | 22839504 | 22840700 | 6419 | 33 |
| 9 | 22843086 | R.D. | - | BTD_BOVIN | 22841512 | 22841913 | 1173 | 32 |
| 9 | 22853088 | R.D. | - | KLF17_HUMAN | 22845184 | 22845444 | 7644 | 40 |
| 9 | 22863089 | F.D. | + | HACL1_MOUSE | 22873646 | 22873843 | 10557 | 33 |
| 9 | 22873090 | F.D. | + | HACL1_MOUSE | 22873646 | 22873843 | 556 | 28 |
| 9 | 22883092 | F.D. | + | HACL1_HUMAN | 22885037 | 22885114 | 1945 | 31 |
| 9 | 22893093 | F.U. | + | HACL1_HUMAN | 22892841 | 22892954 | 139 | 44 |
| 9 | 22903094 | F.D. | + | HACL1_MOUSE | 22903276 | 22903467 | 182 | 34 |
| 9 | 22913095 | F.U. | + | HACL1_HUMAN | 22905175 | 22905204 | 7891 | 32 |
| 9 | 22923097 | F.U. | + | HACL1_HUMAN | 22905175 | 22905204 | 17893 | 43 |
| 9 | 22933098 | F.U. | + | HACL1_HUMAN | 22905175 | 22905204 | 27894 | 36 |
| 9 | 22943099 | F.D. | + | COLQ_HUMAN | 22972662 | 22972760 | 29563 | 33 |
| 9 | 22953101 | F.D. | + | COLQ_HUMAN | 22972662 | 22972760 | 19561 | 31 |
| 9 | 22983105 | F.D. | + | COLQ_RAT | 22985503 | 22985736 | 2398 | 34 |
| 9 | 22993106 | F.U. | + | COLQ_HUMAN | 22990401 | 22990466 | 2640 | 32 |
| 9 | 23013109 | F.U. | + | COLQ_HUMAN | 22990401 | 22990466 | 22643 | 22 |
| 9 | 23053114 | F.U. | + | COLQ_HUMAN | 22990401 | 22990466 | 62648 | 684 |
| 9 | 23113122 | F.U. | + | COLQ_HUMAN | 22990401 | 22990466 | 122656 | 26 |
| 9 | 23123123 | F.U. | + | COLQ_HUMAN | 22990401 | 22990466 | 132657 | 28 |
| 9 | 23133125 | F.U. | + | COLQ_HUMAN | 22990401 | 22990466 | 142659 | 33 |
| 9 | 23143126 | R.U. | - | BTD_HUMAN | 23279399 | 23279470 | 136273 | 32 |
| 9 | 23153127 | R.U. | - | BTD_HUMAN | 23279399 | 23279470 | 126272 | 33 |
| 9 | 23163129 | R.U. | - | BTD_HUMAN | 23279399 | 23279470 | 116270 | 30 |
| 9 | 23173130 | R.U. | - | BTD_HUMAN | 23279399 | 23279470 | 106269 | 52 |
| 9 | 23183131 | R.U. | - | BTD_HUMAN | 23279399 | 23279470 | 96268 | 28 |
| 9 | 23193133 | R.U. | - | BTD_HUMAN | 23279399 | 23279470 | 86266 | 32 |
| 9 | 23203134 | R.U. | - | BTD_HUMAN | 23279399 | 23279470 | 76265 | 35 |
| 9 | 23213135 | R.U. | - | BTD_HUMAN | 23279399 | 23279470 | 66264 | 40 |
| 9 | 23223137 | R.U. | - | BTD_HUMAN | 23279399 | 23279470 | 56262 | 37 |
| 9 | 23233138 | R.U. | - | BTD_HUMAN | 23279399 | 23279470 | 46261 | 35 |
| 9 | 23243139 | R.U. | - | BTD_HUMAN | 23279399 | 23279470 | 36260 | 36 |
| 9 | 23253141 | R.U. | - | BTD_HUMAN | 23279399 | 23279470 | 26258 | 45 |
| 9 | 23263142 | R.U. | - | BTD_HUMAN | 23279399 | 23279470 | 16257 | 36 |
| 9 | 23273143 | R.U. | - | BTD_HUMAN | 23279399 | 23279470 | 6256 | 40 |
| 9 | 23313149 | F.D. | + | DEN6A_HUMAN | 23313783 | 23313821 | 634 | 38 |
| 9 | 27973767 | F.U. | + | CAN7_PIG | 27972560 | 27972646 | 1121 | 41 |
| 9 | 27983768 | F.D. | + | CAN7_PIG | 27983861 | 27983965 | 93 | 39 |
| 9 | 27993769 | R.D. | - | 3BP5_MOUSE | 27993213 | 27993255 | 514 | 45 |
| 9 | 28003771 | R.D. | - | METL6_RAT | 27995180 | 27995314 | 8457 | 66 |
| 9 | 28013772 | R.D. | - | METL6_RAT | 27995180 | 27995314 | 18458 | 35 |
| 9 | 28023773 | R.D. | - | METL6_RAT | 27995180 | 27995314 | 28459 | 33 |
| 9 | 28033775 | R.D. | - | METL6_RAT | 27995180 | 27995314 | 38461 | 40 |
| 9 | 28043776 | R.U. | - | 3BP5_MOUSE | 28076503 | 28076691 | 32727 | 37 |
| 9 | 28053777 | R.U. | - | 3BP5_MOUSE | 28076503 | 28076691 | 22726 | 31 |
| 9 | 28063779 | R.U. | - | 3BP5_MOUSE | 28076503 | 28076691 | 12724 | 33 |
| 9 | 28073780 | R.U. | - | 3BP5_MOUSE | 28076503 | 28076691 | 2723 | 38 |
| 9 | 28083781 | R.U. | - | METL6_HUMAN | 28087426 | 28087596 | 3645 | 39 |
| 9 | 28093783 | F.D. | + | EAF1_PONAB | 28093853 | 28093960 | 70 | 30 |
| 9 | 28103784 | F.D. | + | EAF1_PONAB | 28103984 | 28104229 | 200 | 30 |
| 9 | 28113785 | F.U. | + | EAF1_PONAB | 28106371 | 28106414 | 7371 | 32 |
| 9 | 34264601 | R.D. | - | TXD16_MOUSE | 34238730 | 34238914 | 25687 | 33 |
| 9 | 34294605 | R.D. | - | TXD16_MOUSE | 34238730 | 34238914 | 55691 | 18 |
| 9 | 43365808 | R.U. | - | D39U1_HUMAN | 43381126 | 43381383 | 15318 | 55 |
| 9 | 43515828 | R.U. | - | MCPTX_MOUSE | 43521148 | 43521228 | 5320 | 31 |
| 11 | 9110720 | F.D. | + | LYPL1_MOUSE | 9330406 | 9330501 | 219686 | 0 |
| 11 | 9120720 | F.D. | + | LYPL1_MOUSE | 9330406 | 9330501 | 209686 | 1 |
| 11 | 9800774 | F.U. | + | RS11_CANLF | 9670774 | 9671254 | 129520 | 28 |
| 11 | 9810775 | F.U. | + | RS11_CANLF | 9670774 | 9671254 | 139521 | 20 |
| 11 | 9980788 | R.D. | - | ZNT10_HUMAN | 9977745 | 9977990 | 2798 | 28 |
| 11 | 10070795 | R.U. | - | ZNT10_MOUSE | 10087665 | 10087838 | 16870 | 9 |
| 11 | 10790852 | F.D. | + | BEX4_MOUSE | 10849548 | 10849862 | 58696 | 19 |
| 11 | 10800853 | F.D. | + | BEX4_MOUSE | 10849548 | 10849862 | 48695 | 36 |
| 11 | 11210885 | R.U. | - | CF136_HUMAN | 11407355 | 11407558 | 196470 | 16 |
| 11 | 11250889 | R.U. | - | CF136_HUMAN | 11407355 | 11407558 | 156466 | 9 |
| 11 | 11420902 | R.D. | - | DUS10_MOUSE | 11411998 | 11412507 | 8395 | 0 |
| 11 | 12520989 | R.D. | - | CAN8_MOUSE | 12433523 | 12436096 | 84893 | 26 |
| 11 | 12530990 | R.D. | - | CAN8_MOUSE | 12433523 | 12436096 | 94894 | 21 |
| 11 | 12540990 | R.D. | - | CAN8_MOUSE | 12433523 | 12436096 | 104894 | 30 |
| 11 | 12550991 | F.D. | + | CC185_HUMAN | 12662517 | 12664382 | 111526 | 26 |
| 11 | 12560992 | F.D. | + | CC185_HUMAN | 12662517 | 12664382 | 101525 | 17 |
| 12 | 5090658 | F.D. | + | RUNX3_HUMAN | 5420888 | 5421175 | 330230 | 32 |
| 12 | 5100659 | F.D. | + | RUNX3_HUMAN | 5420888 | 5421175 | 320229 | 44 |
| 12 | 5110660 | F.D. | + | RUNX3_HUMAN | 5420888 | 5421175 | 310228 | 31 |
| 13 | 12381705 | R.U. | - | SO4C1_RAT | 12429261 | 12429794 | 47556 | 27 |
| 18 | 250119 | R.D. | - | NUD4B_HUMAN | 206669 | 207358 | 42761 | 96 |
| 18 | 260120 | R.U. | - | SHCAF_HUMAN | 309357 | 309605 | 49237 | 72 |
| 18 | 310129 | R.D. | - | AL1L2_HUMAN | 309602 | 309706 | 423 | 30 |
| 18 | 320130 | R.U. | - | NUDT3_BOVIN | 329664 | 329716 | 9534 | 37 |
| 18 | 330132 | R.D. | - | AL1A1_HUMAN | 329664 | 329716 | 416 | 30 |
| 18 | 340133 | R.U. | - | AL1A1_HUMAN | 341219 | 341359 | 1086 | 75 |
| 18 | 350135 | R.U. | - | AL1L2_HUMAN | 350255 | 350413 | 120 | 37 |
| 18 | 360137 | R.U. | - | AL1L2_HUMAN | 360710 | 360946 | 573 | 36 |
| 18 | 370138 | R.D. | - | DP13B_HUMAN | 363004 | 363163 | 6975 | 34 |
| 18 | 380140 | F.D. | + | WASC4_MOUSE | 381461 | 381613 | 1321 | 30 |
| 18 | 390141 | F.U. | + | WASC4_MOUSE | 389224 | 389352 | 789 | 39 |
| 18 | 400143 | F.U. | + | WASC4_MOUSE | 398939 | 399187 | 956 | 42 |
| 18 | 410145 | F.D. | + | WASC4_MOUSE | 410176 | 410331 | 31 | 34 |
| 18 | 420146 | F.U. | + | WASC4_MOUSE | 419829 | 419963 | 183 | 33 |
| 18 | 430148 | F.U. | + | WASC4_MOUSE | 429607 | 429804 | 344 | 32 |
| 18 | 440150 | R.U. | - | AL1L2_HUMAN | 441397 | 441531 | 1247 | 31 |
| 18 | 470154 | R.D. | - | DP13A_HUMAN | 469669 | 469728 | 426 | 57 |
| 18 | 480156 | R.D. | - | LA_HUMAN | 479562 | 479663 | 493 | 37 |
| 18 | 490158 | F.D. | + | HNRPK_BOVIN | 496119 | 496667 | 5961 | 33 |
| 22 | 2531126 | R.D. | - | NOL10_HUMAN | 2490811 | 2490912 | 40214 | 382 |
| 22 | 2541128 | R.D. | - | NOL10_HUMAN | 2490811 | 2490912 | 50216 | 36 |
| 22 | 2551131 | R.U. | - | DCOR_RAT | 2600918 | 2601037 | 49787 | 38 |
| 22 | 2561133 | R.U. | - | DCOR_RAT | 2600918 | 2601037 | 39785 | 37 |
| 22 | 2711164 | F.D. | + | VATC1_MOUSE | 2713358 | 2713504 | 2194 | 30 |
| 22 | 2721166 | F.U. | + | VATC2_MOUSE | 2715306 | 2715373 | 5793 | 31 |
| 22 | 3141255 | R.U. | - | ROCK1_RAT | 3144795 | 3144947 | 3540 | 47 |
| 22 | 3151257 | R.D. | - | E2F6_BOVIN | 3150535 | 3150834 | 423 | 81 |
| 22 | 3161259 | R.D. | - | NTR2_RAT | 3158913 | 3159026 | 2233 | 203 |
| 22 | 3171261 | R.D. | - | NTR2_RAT | 3158913 | 3159026 | 12235 | 83 |
| 22 | 3181263 | R.D. | - | NTR2_RAT | 3158913 | 3159026 | 22237 | 91 |
| 22 | 3191265 | R.D. | - | NTR2_RAT | 3158913 | 3159026 | 32239 | 56 |
| 22 | 3201267 | R.D. | - | NTR2_RAT | 3158913 | 3159026 | 42241 | 159 |
| 22 | 4731590 | R.D. | - | HDAC1_HUMAN | 4453010 | 4453411 | 278179 | 64 |
| 22 | 4741592 | R.D. | - | HDAC1_HUMAN | 4453010 | 4453411 | 288181 | 85 |
| 22 | 6081874 | F.U. | + | FIS1_MOUSE | 6070237 | 6070692 | 11182 | 46 |
| 22 | 6091876 | F.U. | + | FIS1_MOUSE | 6070237 | 6070692 | 21184 | 37 |
| 22 | 6161891 | F.U. | + | PIN4_BOVIN | 6160326 | 6160529 | 1362 | 30 |
| 22 | 6171893 | F.U. | + | PIN4_BOVIN | 6160326 | 6160529 | 11364 | 28 |
| 22 | 6281916 | F.U. | + | PIN4_BOVIN | 6160326 | 6160529 | 121387 | 6 |
| 22 | 17954374 | R.D. | - | ADCY3_MOUSE | 17942863 | 17943297 | 11077 | 41 |
| 23 | 23595005 | F.U. | + | S15A3_RAT | 23461323 | 23461472 | 133533 | 32 |
| 23 | 23605007 | F.U. | + | S15A3_RAT | 23461323 | 23461472 | 143535 | 45 |
| 23 | 23615009 | F.U. | + | S15A3_RAT | 23461323 | 23461472 | 153537 | 176 |
| 23 | 23625011 | F.U. | + | S15A3_RAT | 23461323 | 23461472 | 163539 | 45 |
| 23 | 23635013 | F.U. | + | S15A3_RAT | 23461323 | 23461472 | 173541 | 44 |
| 23 | 23645015 | F.U. | + | S15A3_RAT | 23461323 | 23461472 | 183543 | 16 |
| 23 | 23705028 | F.U. | + | S15A3_RAT | 23461323 | 23461472 | 243556 | 40 |
| 23 | 23715030 | F.U. | + | S15A3_RAT | 23461323 | 23461472 | 253558 | 34 |
| 23 | 28045938 | F.D. | + | TECT1_HUMAN | 28048351 | 28048533 | 2413 | 52 |
| 23 | 28055940 | F.U. | + | TECT1_MOUSE | 28055512 | 28055642 | 298 | 62 |
| 23 | 28075945 | R.D. | - | PP1A_RABIT | 28075268 | 28075555 | 390 | 66 |
| 23 | 28085947 | R.D. | - | PP1A_RABIT | 28075268 | 28075555 | 10392 | 35 |
| 23 | 28095949 | R.U. | - | HVCN1_MOUSE | 28110939 | 28110965 | 14990 | 60 |
| 23 | 28265984 | R.D. | - | ATX2_MOUSE | 28204421 | 28204591 | 61393 | 20 |
| 23 | 30096368 | R.D. | - | TBX5_HUMAN | 30039131 | 30039349 | 57019 | 32 |
| 23 | 30106370 | R.D. | - | TBX5_HUMAN | 30039131 | 30039349 | 67021 | 34 |
| 23 | 32746924 | F.U. | + | PEBP1_PONAB | 32687418 | 32687717 | 59207 | 35 |
| 23 | 32756926 | F.U. | + | PEBP1_PONAB | 32687418 | 32687717 | 69209 | 16 |

**Table S11.** Positions (Sweep site) identified as having experienced a recent selective sweep in *P. eremicus* and the nearest gene (Gene id) and distance (in bp) to that gene. Relative location (loc.) indicates whether the site is located inside of a gene or if the nearest gene is upstream (U) or downstream (D) on the sense (F) or antisense (R) strand. Gene direction (dir.) is indicated by a + (forward), - (reverse). The boundaries of the 10 kbp window (5 kbp on either side of the sweep site) are indicated by the start and stop columns, and local coverage (cov.) is reported for each 10 kbp window.

| chr | Sweep site | Relative loc. | Gene dir | Gene id | Start | Stop | Distance | Local cov. |
| --- | --- | --- | --- | --- | --- | --- | --- | --- |
| 1 | 42451454 | gene | - | USH1C_MOUSE | 42450986 | 42451522 | 0 | 17 |
| 8 | 67956 | gene | + | MEIOB_MACFA | 67854 | 68078 | 0 | 10 |
| 1 | 5360184 | A.D. | - | V2116_MOUSE | 5346336 | 5346686 | 13498 | 36 |
| 1 | 5370184 | A.D. | - | V2116_MOUSE | 5346336 | 5346686 | 23498 | 27 |
| 1 | 18770643 | A.D. | - | ZN813_HUMAN | 18766861 | 18767466 | 3177 | 15 |
| 1 | 19020652 | A.D. | - | VN1R1_HUMAN | 19015513 | 19016187 | 4465 | 34 |
| 1 | 54871879 | A.D. | - | MKRN3_HUMAN | 54742206 | 54743414 | 128465 | 13 |
| 1 | 165655672 | A.D. | - | CHM1A_HUMAN | 165381761 | 165382405 | 273267 | 9 |
| 1 | 165685673 | A.D. | - | CHM1A_HUMAN | 165381761 | 165382405 | 303268 | 72 |
| 1 | 165755675 | A.D. | - | CHM1A_HUMAN | 165381761 | 165382405 | 373270 | 22 |
| 1 | 174385971 | A.D. | - | MOT12_RAT | 174361937 | 174363328 | 22643 | 12 |
| 2 | 166155058 | A.D. | - | PRAME_HUMAN | 166153558 | 166153583 | 1475 | 40 |
| 2 | 166975083 | A.D. | - | CD11B_HUMAN | 166974821 | 166974961 | 122 | 14 |
| 3 | 85763515 | A.D. | - | RS2_MOUSE | 85411538 | 85411627 | 351888 | 1 |
| 4 | 93066493 | A.D. | - | RS6_RAT | 92749379 | 92750479 | 316014 | 10 |
| 4 | 93076494 | A.D. | - | RS6_RAT | 92749379 | 92750479 | 326015 | 103 |
| 4 | 93106496 | A.D. | - | RS6_RAT | 92749379 | 92750479 | 356017 | 71 |
| 5 | 79026821 | A.D. | - | RUXG_HUMAN | 78999644 | 79000114 | 26707 | 17 |
| 6 | 43822522 | A.D. | - | ZN431_MOUSE | 43818849 | 43818920 | 3602 | 11 |
| 6 | 55423182 | A.D. | - | PCD18_HUMAN | 54514666 | 54514917 | 908265 | 9 |
| 6 | 66223796 | A.D. | - | ADCL2_HUMAN | 66216663 | 66216881 | 6915 | 22 |
| 7 | 15581336 | A.D. | - | OR5F1_HUMAN | 15577602 | 15578627 | 2709 | 12 |
| 7 | 87877535 | A.D. | - | TRI49_HUMAN | 87867289 | 87867690 | 9845 | 14 |
| 8 | 37610438 | A.D. | - | N4BP3_HUMAN | 37608097 | 37608225 | 2213 | 22 |
| 8 | 37650441 | A.D. | - | RS26_BOVIN | 37631555 | 37631881 | 18560 | 12 |
| 9 | 21751312 | A.D. | - | PTN20_MOUSE | 21750876 | 21751210 | 102 | 17 |
| 9 | 21771313 | A.D. | - | LRC18_HUMAN | 21764814 | 21764972 | 6341 | 23 |
| 9 | 21781314 | A.D. | - | LRC18_HUMAN | 21764814 | 21764972 | 16342 | 25 |
| 9 | 21801315 | A.D. | - | LRC18_HUMAN | 21764814 | 21764972 | 36343 | 19 |
| 9 | 21811315 | A.D. | - | LRC18_HUMAN | 21764814 | 21764972 | 46343 | 23 |
| 9 | 29611786 | A.D. | - | MSMO1_MOUSE | 29591892 | 29592032 | 19754 | 28 |
| 9 | 29921804 | A.D. | - | PPIF_BOVIN | 29895958 | 29896092 | 25712 | 20 |
| 9 | 45652753 | A.D. | - | TV381_HUMAN | 45652349 | 45652672 | 81 | 15 |
| 10 | 48533511 | A.D. | - | AT8A2_HUMAN | 48415818 | 48416384 | 117127 | 0 |
| 10 | 48543511 | A.D. | - | AT8A2_HUMAN | 48415818 | 48416384 | 127127 | 0 |
| 10 | 48553512 | A.D. | - | AT8A2_HUMAN | 48415818 | 48416384 | 137128 | 0 |
| 10 | 48563513 | A.D. | - | AT8A2_HUMAN | 48415818 | 48416384 | 147129 | 0 |
| 10 | 48573513 | A.D. | - | AT8A2_HUMAN | 48415818 | 48416384 | 157129 | 0 |
| 10 | 48583514 | A.D. | - | AT8A2_HUMAN | 48415818 | 48416384 | 167130 | 0 |
| 10 | 48593514 | A.D. | - | AT8A2_HUMAN | 48415818 | 48416384 | 177130 | 0 |
| 11 | 11353441 | A.D. | - | H3X_HUMAN | 10482811 | 10483095 | 870346 | 32 |
| 11 | 11363442 | A.D. | - | H3X_HUMAN | 10482811 | 10483095 | 880347 | 17 |
| 11 | 11413447 | A.D. | - | H3X_HUMAN | 10482811 | 10483095 | 930352 | 30 |
| 11 | 13543664 | A.D. | - | HMGN1_MOUSE | 13386563 | 13386778 | 156886 | 16 |
| 11 | 13573667 | A.D. | - | HMGN1_MOUSE | 13386563 | 13386778 | 186889 | 14 |
| 11 | 15413854 | A.D. | - | HMGN1_BOVIN | 15287950 | 15288192 | 125662 | 20 |
| 11 | 17334049 | A.D. | - | RL7A_MOUSE | 17288310 | 17288948 | 45101 | 17 |
| 11 | 17344050 | A.D. | - | RL7A_MOUSE | 17288310 | 17288948 | 55102 | 32 |
| 11 | 17534070 | A.D. | - | RL38_MOUSE | 17512009 | 17512152 | 21918 | 12 |
| 11 | 18344152 | A.D. | - | MK67I_HUMAN | 18245039 | 18245173 | 98979 | 15 |
| 13 | 5260964 | A.D. | - | GBX2_HUMAN | 5047308 | 5047856 | 213108 | 21 |
| 15 | 11931045 | A.D. | - | IRX2_MOUSE | 11604006 | 11604050 | 326995 | 11 |
| 15 | 25552233 | A.D. | - | RL21_CHILA | 25359646 | 25359771 | 192462 | 1493 |
| 17 | 62652416 | A.D. | - | AP3M1_PONAB | 62624793 | 62624948 | 27468 | 7 |
| 17 | 62662418 | A.D. | - | AP3M1_PONAB | 62624793 | 62624948 | 37470 | 9 |
| 19 | 3230392 | A.D. | - | FRIH_CRIGR | 3195477 | 3195632 | 34760 | 0 |
| 22 | 54190641 | A.D. | - | TM38B_MOUSE | 54164658 | 54165098 | 25543 | 151 |
| 1 | 16870578 | A.U. | - | ZN331_PONAB | 16894202 | 16894280 | 23624 | 7 |
| 1 | 16880578 | A.U. | - | ZN331_PONAB | 16894202 | 16894280 | 13624 | 6 |
| 1 | 16890579 | A.U. | - | ZN331_PONAB | 16894202 | 16894280 | 3623 | 8 |
| 1 | 16900579 | A.U. | - | ZN772_HUMAN | 16902945 | 16903925 | 2366 | 12 |
| 1 | 20950718 | A.U. | - | ST2A1_MOUSE | 20971676 | 20971786 | 20958 | 17 |
| 2 | 110080 | A.U. | - | PAFA_CAVPO | 133861 | 135171 | 23781 | 42 |
| 2 | 45761449 | A.U. | - | C1GLT_BOVIN | 45949974 | 45950255 | 188525 | 1 |
| 2 | 45771449 | A.U. | - | C1GLT_BOVIN | 45949974 | 45950255 | 178525 | 5 |
| 2 | 90862801 | A.U. | - | RS25_BOVIN | 91078575 | 91079711 | 215774 | 10 |
| 2 | 128773938 | A.U. | - | TMM61_HUMAN | 128862093 | 128862173 | 88155 | 16 |
| 4 | 420030 | A.U. | - | ANO3_HUMAN | 467940 | 468907 | 47910 | 30 |
| 4 | 82295742 | A.U. | - | PRRX2_HUMAN | 82330217 | 82330294 | 34475 | 10 |
| 6 | 59643422 | A.U. | - | FXO3B_HUMAN | 59668190 | 59668411 | 24768 | 42 |
| 6 | 82994751 | A.U. | - | MARE1_MOUSE | 83018326 | 83018424 | 23575 | 7 |
| 6 | 125487168 | A.U. | - | O52R1_HUMAN | 125494898 | 125495206 | 7730 | 13 |
| 8 | 3638192 | A.U. | - | MRP3_MOUSE | 3640178 | 3640492 | 1986 | 22 |
| 8 | 37600437 | A.U. | - | RMD5B_HUMAN | 37600475 | 37600630 | 38 | 47 |
| 8 | 37620439 | A.U. | - | RMD5A_MOUSE | 37629249 | 37629776 | 8810 | 10 |
| 8 | 53651498 | A.U. | - | DYH9_HUMAN | 53652515 | 53652877 | 1017 | 8 |
| 9 | 21571301 | A.U. | - | GDF10_RAT | 21745996 | 21746241 | 174695 | 18 |
| 9 | 21581302 | A.U. | - | GDF10_RAT | 21745996 | 21746241 | 164694 | 32 |
| 9 | 21591302 | A.U. | - | GDF10_RAT | 21745996 | 21746241 | 154694 | NA |
| 9 | 21601303 | A.U. | - | GDF10_RAT | 21745996 | 21746241 | 144693 | NA |
| 9 | 21611303 | A.U. | - | GDF10_RAT | 21745996 | 21746241 | 134693 | 23 |
| 9 | 21621304 | A.U. | - | GDF10_RAT | 21745996 | 21746241 | 124692 | 21 |
| 9 | 21631305 | A.U. | - | GDF10_RAT | 21745996 | 21746241 | 114691 | 27 |
| 9 | 21701309 | A.U. | - | GDF10_RAT | 21745996 | 21746241 | 44687 | NA |
| 9 | 21711309 | A.U. | - | GDF10_RAT | 21745996 | 21746241 | 34687 | 41 |
| 9 | 21721310 | A.U. | - | GDF10_RAT | 21745996 | 21746241 | 24686 | 27 |
| 9 | 21731311 | A.U. | - | GDF10_RAT | 21745996 | 21746241 | 14685 | 22 |
| 9 | 21741311 | A.U. | - | GDF10_RAT | 21745996 | 21746241 | 4685 | 17 |
| 9 | 21761312 | A.U. | - | PTN20_MOUSE | 21761828 | 21761918 | 516 | NA |
| 9 | 29591785 | A.U. | - | RPC1_BOVIN | 29591892 | 29592032 | 107 | 69 |
| 9 | 29811798 | A.U. | - | RPC1_BOVIN | 29868985 | 29869179 | 57187 | 30 |
| 9 | 29891803 | A.U. | - | MSMO1_MACFA | 29895412 | 29895666 | 3609 | 18 |
| 11 | 11963503 | A.U. | - | RL15_MOUSE | 13386530 | 13386772 | 1423027 | 26 |
| 11 | 11983505 | A.U. | - | RL15_MOUSE | 13386530 | 13386772 | 1403025 | 30 |
| 11 | 12013508 | A.U. | - | RL15_MOUSE | 13386530 | 13386772 | 1373022 | 15 |
| 11 | 12593567 | A.U. | - | RL15_MOUSE | 13386530 | 13386772 | 792963 | 15 |
| 11 | 12603568 | A.U. | - | RL15_MOUSE | 13386530 | 13386772 | 782962 | 15 |
| 11 | 13253635 | A.U. | - | RL15_MOUSE | 13386530 | 13386772 | 132895 | 19 |
| 11 | 16833998 | A.U. | - | HMGN1_BOVIN | 17287078 | 17287548 | 453080 | 30 |
| 11 | 17254041 | A.U. | - | HMGN1_BOVIN | 17287078 | 17287548 | 33037 | 17 |
| 11 | 66409038 | A.U. | - | XCL1_RAT | 66410553 | 66410843 | 1515 | 17 |
| 11 | 66419039 | A.U. | - | TBX19_HUMAN | 66421651 | 66421760 | 2612 | 20 |
| 12 | 14131409 | A.U. | - | YJU2_HUMAN | 14154248 | 14154706 | 22839 | 4 |
| 12 | 76787656 | A.U. | - | FGD4_RAT | 76843804 | 76844043 | 56148 | 18 |
| 17 | 62672420 | A.U. | - | IKKB_RAT | 62703493 | 62703591 | 31073 | 6 |
| 22 | 48859689 | A.U. | - | V2116_MOUSE | 48872506 | 48873063 | 12817 | 23 |
| 22 | 58621431 | A.U. | - | MADCA_MOUSE | 58669362 | 58669655 | 47931 | 19 |
| 1 | 5440187 | S.D. | + | V2116_MOUSE | 5449087 | 5449431 | 8900 | 43 |
| 1 | 10360355 | S.D. | + | V2116_MOUSE | 10437321 | 10437566 | 76966 | 40 |
| 1 | 17000583 | S.D. | + | ZN426_HUMAN | 17004893 | 17005090 | 4310 | 10 |
| 1 | 17020583 | S.D. | + | VN1R1_HUMAN | 17024039 | 17024839 | 3456 | 16 |
| 1 | 21030720 | S.D. | + | ST2A1_RAT | 21034561 | 21034695 | 3841 | 11 |
| 1 | 29691017 | S.D. | + | PHF8_HUMAN | 29738248 | 29740893 | 47231 | 69 |
| 1 | 42541457 | S.D. | + | OTOG_HUMAN | 42541568 | 42542217 | 111 | 12 |
| 1 | 42551457 | S.D. | + | OTOG_HUMAN | 42552966 | 42553031 | 1509 | 15 |
| 1 | 43111476 | S.D. | + | MRGB5_RAT | 43138353 | 43138544 | 26877 | 9 |
| 1 | 43211480 | S.D. | + | MRGB5_RAT | 43233908 | 43234915 | 22428 | 8 |
| 1 | 43221480 | S.D. | + | MRGB5_RAT | 43233908 | 43234915 | 12428 | 8 |
| 1 | 48081647 | S.D. | + | RL36_PONAB | 48135178 | 48135339 | 53531 | 31 |
| 1 | 48111648 | S.D. | + | RL36_PONAB | 48135178 | 48135339 | 23530 | 22 |
| 1 | 107073666 | S.D. | + | PARVB_MOUSE | 107080328 | 107080471 | 6662 | 137 |
| 1 | 152625226 | S.D. | + | OR2T4_HUMAN | 152626478 | 152627488 | 1252 | 14 |
| 1 | 174395971 | S.D. | + | IFT1B_HUMAN | 174411370 | 174411921 | 15399 | 32 |
| 3 | 81853367 | S.D. | + | MIC60_RAT | 81891465 | 81891538 | 38098 | 14 |
| 3 | 81883368 | S.D. | + | MIC60_RAT | 81891465 | 81891538 | 8097 | 18 |
| 4 | 94736610 | S.D. | + | KYNU_HUMAN | 94876473 | 94876595 | 139863 | 152 |
| 4 | 94756611 | S.D. | + | KYNU_HUMAN | 94876473 | 94876595 | 119862 | 1170 |
| 4 | 94776612 | S.D. | + | KYNU_HUMAN | 94876473 | 94876595 | 99861 | 1271 |
| 4 | 94786613 | S.D. | + | KYNU_HUMAN | 94876473 | 94876595 | 89860 | 226 |
| 4 | 94806614 | S.D. | + | KYNU_HUMAN | 94876473 | 94876595 | 69859 | 21 |
| 4 | 94816615 | S.D. | + | KYNU_HUMAN | 94876473 | 94876595 | 59858 | 6 |
| 4 | 94836617 | S.D. | + | KYNU_HUMAN | 94876473 | 94876595 | 39856 | 27 |
| 4 | 134529386 | S.D. | + | FSIP2_MOUSE | 134566770 | 134566892 | 37384 | 10 |
| 6 | 77924462 | S.D. | + | RL36_PONAB | 77931107 | 77931352 | 6645 | 17 |
| 6 | 82304711 | S.D. | + | PLD3B_CRIGR | 82316183 | 82316522 | 11472 | 20 |
| 7 | 87797528 | S.D. | + | TR43B_HUMAN | 87798962 | 87799354 | 1434 | 5 |
| 7 | 125560766 | S.D. | + | ZN529_HUMAN | 125561802 | 125562353 | 1036 | 10 |
| 8 | 37100404 | S.D. | + | CLK1_MOUSE | 37135633 | 37135773 | 35229 | 117 |
| 8 | 37120406 | S.D. | + | CLK1_MOUSE | 37135633 | 37135773 | 15227 | 71 |
| 8 | 37570435 | S.D. | + | ZMAT2_HUMAN | 37570757 | 37571353 | 322 | 87 |
| 9 | 21941323 | S.D. | + | TPT1L_HUMAN | 22049000 | 22049290 | 107677 | NA |
| 9 | 29501779 | S.D. | + | RPC1_BOVIN | 29502439 | 29502543 | 660 | 23 |
| 9 | 29731793 | S.D. | + | DPPA3_MOUSE | 29750411 | 29750866 | 18618 | 160 |
| 10 | 48603515 | S.D. | + | SHSA3_HUMAN | 48779230 | 48779506 | 175715 | 0 |
| 11 | 10103314 | S.D. | + | RL15_MOUSE | 10252681 | 10252965 | 149367 | 22 |
| 11 | 10123316 | S.D. | + | RL15_MOUSE | 10252681 | 10252965 | 129365 | 13 |
| 11 | 14523764 | S.D. | + | PAI2B_MOUSE | 14666771 | 14667106 | 143007 | 9 |
| 11 | 14533765 | S.D. | + | PAI2B_MOUSE | 14666771 | 14667106 | 133006 | 7 |
| 11 | 15683882 | S.D. | + | ROA2_RAT | 15725772 | 15725982 | 41890 | 15 |
| 11 | 17544071 | S.D. | + | SNPC1_MOUSE | 17575392 | 17575574 | 31321 | 73 |
| 11 | 66349032 | S.D. | + | GP161_BOVIN | 66361669 | 66362055 | 12637 | 12 |
| 11 | 66359033 | S.D. | + | GP161_BOVIN | 66361669 | 66362055 | 2636 | 24 |
| 12 | 79207897 | S.D. | + | IMPG2_MOUSE | 79231056 | 79231277 | 23159 | 5 |
| 13 | 5330977 | S.D. | + | SH3B4_HUMAN | 5545804 | 5545920 | 214827 | 2719 |
| 13 | 5340978 | S.D. | + | SH3B4_HUMAN | 5545804 | 5545920 | 204826 | 20 |
| 13 | 5350980 | S.D. | + | SH3B4_HUMAN | 5545804 | 5545920 | 194824 | 16 |
| 13 | 5370984 | S.D. | + | SH3B4_HUMAN | 5545804 | 5545920 | 174820 | 15 |
| 13 | 5410991 | S.D. | + | SH3B4_HUMAN | 5545804 | 5545920 | 134813 | 27 |
| 13 | 5420992 | S.D. | + | SH3B4_HUMAN | 5545804 | 5545920 | 124812 | 24 |
| 15 | 10230896 | S.D. | + | DPCA2_HUMAN | 10314944 | 10315318 | 84048 | 9 |
| 17 | 27845563 | S.D. | + | JHY_BOVIN | 27861441 | 27862091 | 15878 | 7 |
| 20 | 17873229 | S.D. | + | ADCK5_HUMAN | 17874752 | 17874955 | 1523 | 4 |
| 21 | 18212928 | S.D. | + | HA10_MOUSE | 18215372 | 18215614 | 2444 | 5 |
| 21 | 63059316 | S.D. | + | GSTM5_RAT | 63136709 | 63136738 | 77393 | 41 |
| 21 | 63099321 | S.D. | + | GSTM5_RAT | 63136709 | 63136738 | 37388 | 26 |
| 21 | 63109323 | S.D. | + | GSTM5_RAT | 63136709 | 63136738 | 27386 | 29 |
| 22 | 19284410 | S.D. | + | NTR1_RAT | 19744741 | 19745331 | 460331 | 9 |
| 23 | 36755600 | S.D. | + | V2R26_MOUSE | 36827765 | 36828118 | 72165 | 11 |
| 23 | 36765602 | S.D. | + | V2R26_MOUSE | 36827765 | 36828118 | 62163 | 5 |
| 23 | 36775603 | S.D. | + | V2R26_MOUSE | 36827765 | 36828118 | 52162 | 15 |
| 23 | 36785605 | S.D. | + | V2R26_MOUSE | 36827765 | 36828118 | 42160 | 4 |
| 1 | 5430186 | S.U. | + | V2R26_MOUSE | 5427277 | 5427876 | 2310 | 41 |
| 1 | 16530566 | S.U. | + | UBP29_HUMAN | 16518402 | 16519076 | 11490 | 85 |
| 1 | 16860578 | S.U. | + | ZN304_HUMAN | 16836346 | 16838171 | 22407 | 10 |
| 1 | 16910579 | S.U. | + | ZN773_HUMAN | 16910277 | 16910402 | 177 | 9 |
| 1 | 17010583 | S.U. | + | CCNH_MOUSE | 17009028 | 17009258 | 1325 | 13 |
| 1 | 18280626 | S.U. | + | VN1R4_HUMAN | 18271989 | 18272300 | 8326 | 8 |
| 1 | 18290627 | S.U. | + | VN1R4_HUMAN | 18271989 | 18272300 | 18327 | 14 |
| 1 | 18860646 | S.U. | + | VN1R3_HUMAN | 18840461 | 18841084 | 19562 | 17 |
| 1 | 18880647 | S.U. | + | VN1R3_HUMAN | 18840461 | 18841084 | 39563 | 15 |
| 1 | 21170725 | S.U. | + | SELW_MOUSE | 21141982 | 21142230 | 28495 | 5 |
| 1 | 47641632 | S.U. | + | GAS2_MOUSE | 47409827 | 47409937 | 231695 | 1479 |
| 1 | 47651632 | S.U. | + | GAS2_MOUSE | 47409827 | 47409937 | 241695 | 2877 |
| 1 | 47671633 | S.U. | + | GAS2_MOUSE | 47409827 | 47409937 | 261696 | 80 |
| 1 | 47681633 | S.U. | + | GAS2_MOUSE | 47409827 | 47409937 | 271696 | 28 |
| 1 | 48181650 | S.U. | + | RL36_PONAB | 48135178 | 48135339 | 46311 | 15 |
| 1 | 48251652 | S.U. | + | RL36_PONAB | 48135178 | 48135339 | 116313 | 5 |
| 1 | 48501661 | S.U. | + | RL36_PONAB | 48135178 | 48135339 | 366322 | 362 |
| 1 | 101633480 | S.U. | + | CDC5L_BOVIN | 101571536 | 101571601 | 61879 | 19 |
| 1 | 101643480 | S.U. | + | CDC5L_BOVIN | 101571536 | 101571601 | 71879 | 29 |
| 1 | 107093667 | S.U. | + | PARVA_MOUSE | 107089326 | 107089454 | 4213 | 122 |
| 1 | 107103667 | S.U. | + | PARVA_MOUSE | 107102222 | 107102302 | 1365 | 125 |
| 1 | 107113668 | S.U. | + | PARVA_MOUSE | 107109717 | 107109800 | 3868 | 127 |
| 1 | 152635226 | S.U. | + | OR5P2_HUMAN | 152627444 | 152628415 | 6811 | 30 |
| 1 | 163005581 | S.U. | + | APBA1_MOUSE | 162937523 | 162937594 | 67987 | 1 |
| 1 | 174345969 | S.U. | + | IFIT3_PANTR | 174340082 | 174341545 | 4424 | 14 |
| 2 | 21660726 | S.U. | + | PCBP1_MOUSE | 21594138 | 21594428 | 66298 | 15 |
| 3 | 71502975 | S.U. | + | VOPP1_BOVIN | 71500692 | 71500829 | 2146 | 4 |
| 3 | 71512976 | S.U. | + | VOPP1_BOVIN | 71507132 | 71507320 | 5656 | 12 |
| 3 | 152966057 | S.U. | + | TR107_MOUSE | 152965095 | 152966012 | 45 | 18 |
| 4 | 91486383 | S.U. | + | PPIH_HUMAN | 91376488 | 91376643 | 109740 | 124 |
| 4 | 91526386 | S.U. | + | PPIH_HUMAN | 91376488 | 91376643 | 149743 | 20 |
| 4 | 91536386 | S.U. | + | PPIH_HUMAN | 91376488 | 91376643 | 159743 | 7 |
| 4 | 91556388 | S.U. | + | PPIH_HUMAN | 91376488 | 91376643 | 179745 | 169 |
| 4 | 91616392 | S.U. | + | PPIH_HUMAN | 91376488 | 91376643 | 239749 | 7 |
| 4 | 91626393 | S.U. | + | PPIH_HUMAN | 91376488 | 91376643 | 249750 | 8 |
| 4 | 91636393 | S.U. | + | PPIH_HUMAN | 91376488 | 91376643 | 259750 | 7 |
| 4 | 91956416 | S.U. | + | PPIH_HUMAN | 91376488 | 91376643 | 579773 | 14 |
| 5 | 55094808 | S.U. | + | DRC4_BOVIN | 55072638 | 55072703 | 22105 | 7 |
| 5 | 55104809 | S.U. | + | DRC4_BOVIN | 55072638 | 55072703 | 32106 | 17 |
| 5 | 55154813 | S.U. | + | DRC4_BOVIN | 55072638 | 55072703 | 82110 | 51 |
| 6 | 44482559 | S.U. | + | PDCD7_HUMAN | 44424583 | 44424927 | 57632 | 16 |
| 6 | 59603420 | S.U. | + | FKB1A_RABIT | 59599057 | 59599152 | 4268 | 37 |
| 6 | 77934463 | S.U. | + | RL36_PONAB | 77931107 | 77931392 | 3071 | 9 |
| 7 | 15241307 | S.U. | + | OR4A5_HUMAN | 15233138 | 15233377 | 7930 | 12 |
| 7 | 87917538 | S.U. | + | ZN454_HUMAN | 87900492 | 87900761 | 16777 | 73 |
| 8 | 37580436 | S.U. | + | ZMAT2_HUMAN | 37570757 | 37571353 | 9083 | 109 |
| 8 | 45930988 | S.U. | + | WNT2B_HUMAN | 45910464 | 45910958 | 20030 | 16 |
| 9 | 32151939 | S.U. | + | RL9_MACFA | 32142108 | 32142581 | 9358 | NA |
| 10 | 104027020 | S.U. | + | F110C_RAT | 104009168 | 104010151 | 16869 | 981 |
| 11 | 9143217 | S.U. | + | HS90A_RABIT | 9112325 | 9112990 | 30227 | 15 |
| 11 | 9433246 | S.U. | + | HS90A_RABIT | 9112325 | 9112990 | 320256 | 4 |
| 11 | 66369034 | S.U. | + | GP161_BOVIN | 66366694 | 66367470 | 1564 | 105 |
| 13 | 5861070 | S.U. | + | SH3B4_HUMAN | 5566487 | 5566714 | 294356 | 11 |
| 14 | 38193359 | S.U. | + | HVM04_MOUSE | 38192779 | 38192979 | 380 | 10 |
| 16 | 32035674 | S.U. | + | S18B1_MOUSE | 32035532 | 32035624 | 50 | 19 |
| 18 | 3600934 | S.U. | + | AL1L1_MOUSE | 3447653 | 3447705 | 153229 | 20 |
| 18 | 3610936 | S.U. | + | AL1L1_MOUSE | 3447653 | 3447705 | 163231 | 19 |
| 18 | 3620938 | S.U. | + | AL1L1_MOUSE | 3447653 | 3447705 | 173233 | 26 |
| 18 | 3660947 | S.U. | + | AL1L1_MOUSE | 3447653 | 3447705 | 213242 | 135 |
| 18 | 3700956 | S.U. | + | AL1L1_MOUSE | 3447653 | 3447705 | 253251 | 15 |
| 18 | 3730963 | S.U. | + | AL1L1_MOUSE | 3447653 | 3447705 | 283258 | 12 |
| 20 | 17883231 | S.U. | + | ADCK5_MOUSE | 17880619 | 17880723 | 2508 | 11 |
| 21 | 63139327 | S.U. | + | GSTMU_RABIT | 63138699 | 63138944 | 383 | 21 |
| 21 | 63149328 | S.U. | + | GSTMU_RABIT | 63138699 | 63138944 | 10384 | 16 |
| 22 | 53080442 | S.U. | + | ZN621_HUMAN | 53035428 | 53036189 | 44253 | 5 |
| 22 | 58551419 | S.U. | + | PLPP3_RAT | 58549528 | 58550250 | 1169 | 16 |
| 22 | 58561421 | S.U. | + | PLPP2_MOUSE | 58555461 | 58555574 | 5847 | 19 |
| 22 | 58611430 | S.U. | + | PLPP2_MOUSE | 58555461 | 58555574 | 55856 | 789 |

**Figure S12.** Composite likelihood ratio (CLR) scores for *P. eremicus* based on *Sweepfinder2* results. Values above the horizontal red line surpass the 99.9^th^ percentile. The top five or fewer unique genes are labeled for each chromosome.

**
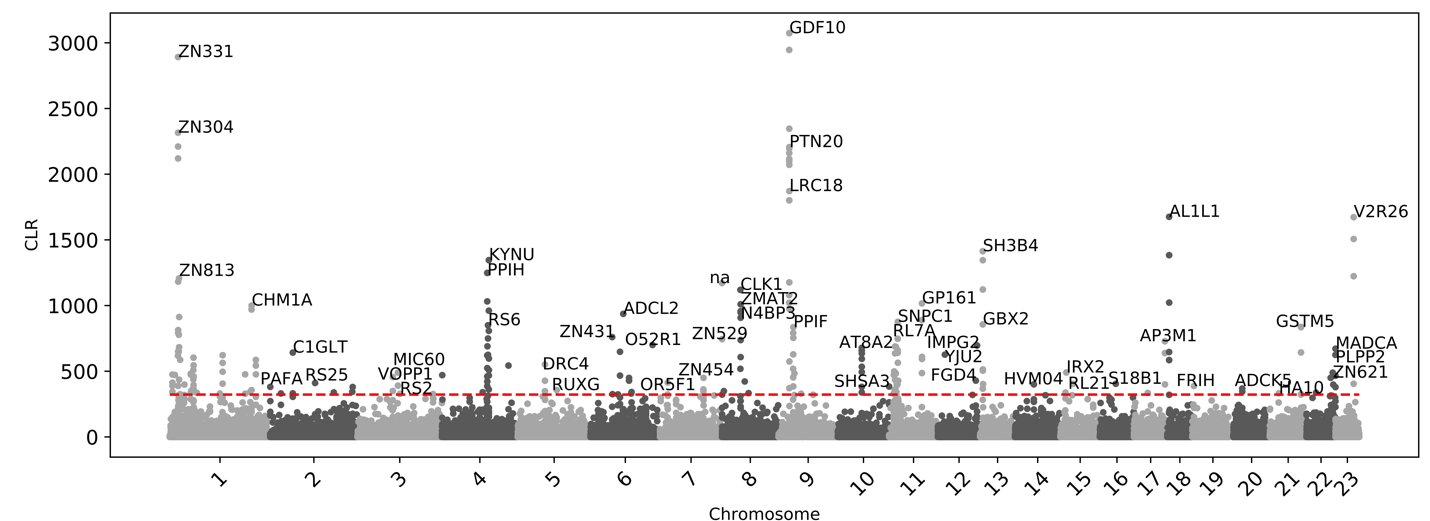
**

**Table S13.** Positions (Sweep site) identified as having experienced a recent selective sweep in *P. maniculatus* and the nearest gene (Gene id) and distance (in bp) to that gene. Relative location (loc.) indicates whether the site is located inside of a gene or if the nearest gene is upstream (U) or downstream (D) on the sense (F) or antisense (R) strand. Gene direction (dir.) is indicated by a + (forward), - (reverse). The boundaries of the 10 kbp window (5 kbp on either side of the sweep site) are indicated by the start and stop columns, and local coverage (cov.) is reported for each 10 kbp window.

| Chr | Sweep site | Relative loc. | Gene dir. | Gene id | Start | Stop | Distance | Local cov. |
| --- | --- | --- | --- | --- | --- | --- | --- | --- |
| 4 | 145409180 | gene | - | ZN217_HUMAN | 145408135 | 145409703 | 0 | 30 |
|  |  |  | - | ZN217_HUMAN | 145409110 | 145409672 | 0 |  |
| 20 | 36260251 | gene | - | DRS7B_HUMAN | 36260227 | 36260883 | 0 | 12 |
|  |  |  | - | DRS7B_RAT | 36260227 | 36260892 | 0 |  |
| 1 | 172106267 | R.U. | - | TM9S3_HUMAN | 172115723 | 172115860 | 9456 | 24 |
| 1 | 172116267 | R.D. | - | BCAP_HUMAN | 172115723 | 172115860 | 407 | 26 |
| 1 | 172126267 | R.D. | - | BCAP_HUMAN | 172123166 | 172123297 | 2970 | 27 |
| 1 | 172136268 | R.D. | - | BCAP_HUMAN | 172135934 | 172135999 | 269 | 29 |
| 1 | 172156268 | R.U. | - | BCAP_HUMAN | 172159291 | 172159480 | 3023 | 31 |
| 1 | 172166269 | R.U. | - | BCAP_MOUSE | 172166585 | 172166743 | 316 | 29 |
| 1 | 172176269 | R.D. | - | CEBOS_MOUSE | 172170983 | 172171129 | 5140 | 25 |
| 1 | 172196270 | R.D. | - | CEBOS_MOUSE | 172170983 | 172171129 | 25141 | 20 |
| 1 | 172206270 | R.D. | - | CEBOS_MOUSE | 172170983 | 172171129 | 35141 | 24 |
| 1 | 172576284 | F.D. | + | RL34_PIG | 172620248 | 172620596 | 43964 | 28 |
| 2 | 158467049 | F.U. | + | LRC38_HUMAN | 158464243 | 158464815 | 2234 | 27 |
| 2 | 158737061 | R.U. | - | TAF10_MOUSE | 158872175 | 158873305 | 135114 | 31 |
| 2 | 158747061 | R.U. | - | TAF10_MOUSE | 158872175 | 158873305 | 125114 | 30 |
| 2 | 158757062 | R.U. | - | TAF10_MOUSE | 158872175 | 158873305 | 115113 | 28 |
| 3 | 40072886 | F.D. | + | RS13_CRIGR | 40124153 | 40124542 | 51267 | 17 |
| 4 | 991016 | R.U. | - | CH10_HUMAN | 998622 | 998738 | 7606 | 24 |
| 4 | 1001017 | R.U. | - | NEK7_RAT | 1001353 | 1001481 | 336 | 30 |
| 4 | 1101023 | R.D. | - | RLA1_RAT | 1089575 | 1089784 | 11239 | 25 |
| 4 | 1111023 | R.D. | - | RLA1_RAT | 1089575 | 1089784 | 21239 | 28 |
| 4 | 1121024 | R.D. | - | RLA1_RAT | 1089575 | 1089784 | 31240 | 24 |
| 4 | 1131024 | R.D. | - | RLA1_RAT | 1089575 | 1089784 | 41240 | 22 |
| 4 | 1141025 | R.D. | - | RLA1_RAT | 1089575 | 1089784 | 51241 | 25 |
| 4 | 1151026 | R.U. | - | PIN4_BOVIN | 1202369 | 1202563 | 51343 | 25 |
| 4 | 1161026 | R.U. | - | PIN4_BOVIN | 1202369 | 1202563 | 41343 | 27 |
| 4 | 1171027 | R.U. | - | PIN4_BOVIN | 1202369 | 1202563 | 31342 | 25 |
| 4 | 1181027 | R.U. | - | PIN4_BOVIN | 1202369 | 1202563 | 21342 | 23 |
| 4 | 1191028 | R.U. | - | PIN4_BOVIN | 1202369 | 1202563 | 11341 | 30 |
| 4 | 1201028 | R.U. | - | PIN4_BOVIN | 1202369 | 1202563 | 1341 | 22 |
| 4 | 1211029 | R.D. | - | LHX2_RAT | 1202381 | 1202563 | 8466 | 26 |
| 4 | 1221029 | R.U. | - | RLA1_RAT | 1234026 | 1234379 | 12997 | 27 |
| 4 | 1231030 | R.U. | - | RLA1_RAT | 1234026 | 1234379 | 2996 | 26 |
| 4 | 1241031 | R.U. | - | LHX2_RAT | 1241522 | 1241737 | 491 | 26 |
| 4 | 1251031 | R.D. | - | CRUM2_MOUSE | 1249846 | 1249974 | 1057 | 24 |
| 4 | 1261032 | R.D. | - | CRUM2_MOUSE | 1249846 | 1249974 | 11058 | 22 |
| 4 | 1271032 | R.D. | - | CRUM2_MOUSE | 1249846 | 1249974 | 21058 | 22 |
| 4 | 1281033 | R.D. | - | CRUM2_MOUSE | 1249846 | 1249974 | 31059 | 27 |
| 4 | 1291033 | R.D. | - | CRUM2_MOUSE | 1249846 | 1249974 | 41059 | 23 |
| 4 | 1301034 | R.D. | - | CRUM2_MOUSE | 1249846 | 1249974 | 51060 | 9 |
| 4 | 1311035 | R.D. | - | CRUM2_MOUSE | 1249846 | 1249974 | 61061 | 15 |
| 4 | 1321035 | R.D. | - | CRUM2_MOUSE | 1249846 | 1249974 | 71061 | 22 |
| 4 | 1331036 | R.D. | - | CRUM2_MOUSE | 1249846 | 1249974 | 81062 | 18 |
| 4 | 1341036 | R.D. | - | CRUM2_MOUSE | 1249846 | 1249974 | 91062 | 18 |
| 4 | 1351037 | R.D. | - | CRUM2_MOUSE | 1249846 | 1249974 | 101063 | 15 |
| 4 | 1361037 | R.D. | - | CRUM2_MOUSE | 1249846 | 1249974 | 111063 | 14 |
| 4 | 1371038 | R.D. | - | CRUM2_MOUSE | 1249846 | 1249974 | 121064 | 16 |
| 4 | 1381039 | R.D. | - | CRUM2_MOUSE | 1249846 | 1249974 | 131065 | 17 |
| 4 | 1391039 | R.D. | - | CRUM2_MOUSE | 1249846 | 1249974 | 141065 | 19 |
| 4 | 1401040 | R.D. | - | CRUM2_MOUSE | 1249846 | 1249974 | 151066 | 18 |
| 4 | 1411040 | R.D. | - | CRUM2_MOUSE | 1249846 | 1249974 | 161066 | 19 |
| 4 | 1421041 | R.D. | - | CRUM2_MOUSE | 1249846 | 1249974 | 171067 | 19 |
| 4 | 1431041 | R.D. | - | CRUM2_MOUSE | 1249846 | 1249974 | 181067 | 18 |
| 4 | 1441042 | R.D. | - | CRUM2_MOUSE | 1249846 | 1249974 | 191068 | 18 |
| 4 | 1451042 | R.D. | - | CRUM2_MOUSE | 1249846 | 1249974 | 201068 | 19 |
| 4 | 1461043 | R.D. | - | CRUM2_MOUSE | 1249846 | 1249974 | 211069 | 16 |
| 4 | 1471044 | R.D. | - | CRUM2_MOUSE | 1249846 | 1249974 | 221070 | 16 |
| 4 | 1481044 | R.D. | - | CRUM2_MOUSE | 1249846 | 1249974 | 231070 | 18 |
| 4 | 1491045 | R.D. | - | CRUM2_MOUSE | 1249846 | 1249974 | 241071 | 17 |
| 4 | 1501045 | R.D. | - | CRUM2_MOUSE | 1249846 | 1249974 | 251071 | 18 |
| 4 | 1511046 | R.D. | - | CRUM2_MOUSE | 1249846 | 1249974 | 261072 | 19 |
| 4 | 1521046 | F.D. | + | DEN1A_HUMAN | 1777255 | 1777377 | 256209 | 19 |
| 4 | 1531047 | F.D. | + | DEN1A_HUMAN | 1777255 | 1777377 | 246208 | 18 |
| 4 | 1541048 | F.D. | + | DEN1A_HUMAN | 1777255 | 1777377 | 236207 | 17 |
| 4 | 1551048 | F.D. | + | DEN1A_HUMAN | 1777255 | 1777377 | 226207 | 20 |
| 4 | 1561049 | F.D. | + | DEN1A_HUMAN | 1777255 | 1777377 | 216206 | 18 |
| 4 | 1571049 | F.D. | + | DEN1A_HUMAN | 1777255 | 1777377 | 206206 | 18 |
| 4 | 1581050 | F.D. | + | DEN1A_HUMAN | 1777255 | 1777377 | 196205 | 20 |
| 4 | 1591050 | F.D. | + | DEN1A_HUMAN | 1777255 | 1777377 | 186205 | 23 |
| 4 | 1601051 | F.D. | + | DEN1A_HUMAN | 1777255 | 1777377 | 176204 | 20 |
| 4 | 1611052 | F.D. | + | DEN1A_HUMAN | 1777255 | 1777377 | 166203 | 23 |
| 4 | 1621052 | F.D. | + | DEN1A_HUMAN | 1777255 | 1777377 | 156203 | 20 |
| 4 | 1631053 | F.D. | + | DEN1A_HUMAN | 1777255 | 1777377 | 146202 | 28 |
| 4 | 1641053 | F.D. | + | DEN1A_HUMAN | 1777255 | 1777377 | 136202 | 23 |
| 4 | 1651054 | F.D. | + | DEN1A_HUMAN | 1777255 | 1777377 | 126201 | 23 |
| 4 | 1661054 | F.D. | + | DEN1A_HUMAN | 1777255 | 1777377 | 116201 | 24 |
| 4 | 1671055 | F.D. | + | DEN1A_HUMAN | 1777255 | 1777377 | 106200 | 23 |
| 4 | 1681055 | F.D. | + | DEN1A_HUMAN | 1777255 | 1777377 | 96200 | 21 |
| 4 | 1691056 | F.D. | + | DEN1A_HUMAN | 1777255 | 1777377 | 86199 | 23 |
| 4 | 1701057 | F.D. | + | DEN1A_HUMAN | 1777255 | 1777377 | 76198 | 25 |
| 4 | 145199168 | F.U. | + | TSH2_PIG | 145160572 | 145163625 | 35543 | 23 |
| 4 | 145289173 | R.U. | - | RS15A_BOVIN | 145406324 | 145406431 | 117151 | 24 |
| 4 | 145299174 | R.U. | - | RS15A_BOVIN | 145406324 | 145406431 | 107150 | 27 |
| 4 | 145309174 | R.U. | - | RS15A_BOVIN | 145406324 | 145406431 | 97150 | 25 |
| 4 | 145319175 | R.U. | - | RS15A_BOVIN | 145406324 | 145406431 | 87149 | 22 |
| 4 | 145329175 | R.U. | - | RS15A_BOVIN | 145406324 | 145406431 | 77149 | 27 |
| 4 | 145339176 | R.U. | - | RS15A_BOVIN | 145406324 | 145406431 | 67148 | 21 |
| 4 | 145349176 | R.U. | - | RS15A_BOVIN | 145406324 | 145406431 | 57148 | 20 |
| 4 | 145359177 | R.U. | - | RS15A_BOVIN | 145406324 | 145406431 | 47147 | 23 |
| 4 | 145369178 | R.U. | - | RS15A_BOVIN | 145406324 | 145406431 | 37146 | 23 |
| 4 | 145379178 | R.U. | - | RS15A_BOVIN | 145406324 | 145406431 | 27146 | 19 |
| 4 | 145389179 | R.U. | - | RS15A_BOVIN | 145406324 | 145406431 | 17145 | 26 |
| 4 | 145399179 | R.U. | - | RS15A_BOVIN | 145406324 | 145406431 | 7145 | 18 |
| 4 | 145419180 | R.D. | - | RS2_MOUSE | 145413031 | 145414434 | 4746 | 24 |
| 4 | 145429181 | R.D. | - | RS2_MOUSE | 145413031 | 145414434 | 14747 | 20 |
| 4 | 145439181 | R.D. | - | RS2_MOUSE | 145413031 | 145414434 | 24747 | 22 |
| 4 | 145449182 | R.D. | - | RS2_MOUSE | 145413031 | 145414434 | 34748 | 23 |
| 4 | 145459183 | R.D. | - | RS2_MOUSE | 145413031 | 145414434 | 44749 | 21 |
| 4 | 145469183 | R.D. | - | RS2_MOUSE | 145413031 | 145414434 | 54749 | 19 |
| 4 | 145479184 | R.D. | - | RS2_MOUSE | 145413031 | 145414434 | 64750 | 19 |
| 4 | 145809202 | R.U. | - | BCAS1_RAT | 145820438 | 145820590 | 11236 | 25 |
| 4 | 145819203 | R.U. | - | BCAS1_RAT | 145820438 | 145820590 | 1235 | 26 |
| 4 | 145829204 | R.U. | - | CP24A_MOUSE | 145829394 | 145829495 | 190 | 26 |
| 4 | 145839204 | R.D. | - | RL24_RAT | 145832693 | 145832983 | 6221 | 22 |
| 4 | 145849205 | R.D. | - | RL24_RAT | 145832693 | 145832983 | 16222 | 26 |
| 4 | 145859205 | R.D. | - | RL24_RAT | 145832693 | 145832983 | 26222 | 27 |
| 4 | 145869206 | R.D. | - | RL24_RAT | 145832693 | 145832983 | 36223 | 21 |
| 4 | 145889207 | R.D. | - | RL24_RAT | 145832693 | 145832983 | 56224 | 25 |
| 4 | 145899207 | R.D. | - | RL24_RAT | 145832693 | 145832983 | 66224 | 24 |
| 4 | 145909208 | R.D. | - | RL24_RAT | 145832693 | 145832983 | 76225 | 22 |
| 4 | 145979212 | R.D. | - | RL24_RAT | 145832693 | 145832983 | 146229 | 24 |
| 4 | 145989213 | R.D. | - | RL24_RAT | 145832693 | 145832983 | 156230 | 21 |
| 4 | 145999213 | R.D. | - | RL24_RAT | 145832693 | 145832983 | 166230 | 22 |
| 4 | 146009214 | F.D. | + | DOK6_HUMAN | 146172874 | 146173197 | 163660 | 19 |
| 4 | 146019214 | F.D. | + | DOK6_HUMAN | 146172874 | 146173197 | 153660 | 18 |
| 4 | 146029215 | F.D. | + | DOK6_HUMAN | 146172874 | 146173197 | 143659 | 26 |
| 4 | 146119220 | F.D. | + | DOK6_HUMAN | 146172874 | 146173197 | 53654 | 24 |
| 4 | 146129221 | F.D. | + | DOK6_HUMAN | 146172874 | 146173197 | 43653 | 21 |
| 9 | 108354070 | R.U. | - | MBNL2_PONAB | 108355740 | 108356486 | 1670 | 21 |
| 9 | 108364071 | R.D. | - | H6ST3_HUMAN | 108355740 | 108356486 | 7585 | 18 |
| 11 | 84936634 | F.U. | + | GPTC2_HUMAN | 84824566 | 84824649 | 111985 | 23 |
| 11 | 84986638 | F.U. | + | GPTC2_HUMAN | 84824566 | 84824649 | 161989 | 24 |
| 11 | 85046642 | F.U. | + | GPTC2_HUMAN | 84824566 | 84824649 | 221993 | 21 |
| 11 | 85056643 | F.U. | + | GPTC2_HUMAN | 84824566 | 84824649 | 231994 | 20 |
| 11 | 85066644 | F.U. | + | GPTC2_HUMAN | 84824566 | 84824649 | 241995 | 16 |
| 11 | 85076645 | F.U. | + | GPTC2_HUMAN | 84824566 | 84824649 | 251996 | 19 |
| 11 | 85086646 | F.U. | + | GPTC2_HUMAN | 84824566 | 84824649 | 261997 | 20 |
| 11 | 85126649 | F.U. | + | GPTC2_HUMAN | 84824566 | 84824649 | 302000 | 17 |
| 11 | 85136649 | F.U. | + | GPTC2_HUMAN | 84824566 | 84824649 | 312000 | 17 |
| 11 | 85146650 | F.U. | + | GPTC2_HUMAN | 84824566 | 84824649 | 322001 | 21 |
| 11 | 85156651 | F.U. | + | GPTC2_HUMAN | 84824566 | 84824649 | 332002 | 23 |
| 11 | 85166652 | F.U. | + | GPTC2_HUMAN | 84824566 | 84824649 | 342003 | 21 |
| 20 | 35660146 | R.D. | - | BOLA2_MOUSE | 35416073 | 35416294 | 243852 | 17 |
| 20 | 35780167 | R.U. | - | RS27_PONAB | 36122955 | 36123134 | 342788 | 17 |
| 20 | 35810172 | R.U. | - | RS27_PONAB | 36122955 | 36123134 | 312783 | 15 |
| 20 | 36050214 | R.U. | - | RS27_PONAB | 36122955 | 36123134 | 72741 | 16 |
| 20 | 36060216 | R.U. | - | RS27_PONAB | 36122955 | 36123134 | 62739 | 17 |
| 20 | 36070217 | R.U. | - | RS27_PONAB | 36122955 | 36123134 | 52738 | 16 |
| 20 | 36080219 | R.U. | - | RS27_PONAB | 36122955 | 36123134 | 42736 | 17 |
| 20 | 36100223 | R.U. | - | RS27_PONAB | 36122955 | 36123134 | 22732 | 11 |
| 20 | 36110224 | R.U. | - | RS27_PONAB | 36122955 | 36123134 | 12731 | 15 |
| 20 | 36130228 | R.D. | - | DRS7B_HUMAN | 36122955 | 36123134 | 7094 | 15 |
| 20 | 36160233 | F.U. | + | PRAL7_MOUSE | 36148466 | 36149380 | 10853 | 16 |
| 20 | 36170235 | F.U. | + | PRAL7_MOUSE | 36148466 | 36149380 | 20855 | 16 |
| 20 | 36180237 | F.U. | + | PRAL7_MOUSE | 36148466 | 36149380 | 30857 | 15 |
| 20 | 36190238 | F.U. | + | PRAL7_MOUSE | 36148466 | 36149380 | 40858 | 13 |
| 20 | 36200240 | F.U. | + | PRAL7_MOUSE | 36148466 | 36149380 | 50860 | 16 |
| 20 | 36210242 | R.U. | - | BOLA2_MOUSE | 36260227 | 36260883 | 49985 | 17 |
| 20 | 36220244 | R.U. | - | BOLA2_MOUSE | 36260227 | 36260883 | 39983 | 18 |
| 20 | 36230245 | R.U. | - | BOLA2_MOUSE | 36260227 | 36260883 | 29982 | 16 |
| 20 | 36240247 | R.U. | - | BOLA2_MOUSE | 36260227 | 36260883 | 19980 | 16 |
| 20 | 36250249 | R.U. | - | BOLA2_MOUSE | 36260227 | 36260883 | 9978 | 18 |
| 20 | 36270252 | R.D. | - | FCF1_MOUSE | 36260888 | 36261070 | 9182 | 15 |
| 20 | 36280254 | R.D. | - | FCF1_MOUSE | 36260888 | 36261070 | 19184 | 21 |
| 20 | 36290256 | R.D. | - | FCF1_MOUSE | 36260888 | 36261070 | 29186 | 16 |
| 20 | 36300258 | R.D. | - | FCF1_MOUSE | 36260888 | 36261070 | 39188 | 17 |
| 20 | 36310259 | R.D. | - | FCF1_MOUSE | 36260888 | 36261070 | 49189 | 17 |
| 20 | 36320261 | R.D. | - | FCF1_MOUSE | 36260888 | 36261070 | 59191 | 15 |
| 20 | 36330263 | R.D. | - | FCF1_MOUSE | 36260888 | 36261070 | 69193 | 18 |
| 20 | 36340265 | R.D. | - | FCF1_MOUSE | 36260888 | 36261070 | 79195 | 16 |
| 20 | 36350266 | R.D. | - | FCF1_MOUSE | 36260888 | 36261070 | 89196 | 14 |
| 20 | 36360268 | R.D. | - | FCF1_MOUSE | 36260888 | 36261070 | 99198 | 17 |
| 20 | 36370270 | R.D. | - | FCF1_MOUSE | 36260888 | 36261070 | 109200 | 15 |
| 20 | 36380272 | R.D. | - | FCF1_MOUSE | 36260888 | 36261070 | 119202 | 17 |
| 20 | 36390273 | R.D. | - | FCF1_MOUSE | 36260888 | 36261070 | 129203 | 17 |
| 20 | 36400275 | R.D. | - | FCF1_MOUSE | 36260888 | 36261070 | 139205 | 17 |
| 20 | 36410277 | R.D. | - | FCF1_MOUSE | 36260888 | 36261070 | 149207 | 16 |
| 20 | 36420279 | R.D. | - | FCF1_MOUSE | 36260888 | 36261070 | 159209 | 16 |
| 20 | 36430280 | R.D. | - | FCF1_MOUSE | 36260888 | 36261070 | 169210 | 16 |
| 20 | 36440282 | R.D. | - | FCF1_MOUSE | 36260888 | 36261070 | 179212 | 18 |
| 20 | 36450284 | R.D. | - | FCF1_MOUSE | 36260888 | 36261070 | 189214 | 16 |
| 20 | 36460286 | R.D. | - | FCF1_MOUSE | 36260888 | 36261070 | 199216 | 18 |
| 20 | 36470287 | R.D. | - | FCF1_MOUSE | 36260888 | 36261070 | 209217 | 16 |
| 20 | 36480289 | R.D. | - | FCF1_MOUSE | 36260888 | 36261070 | 219219 | 14 |
| 20 | 36490291 | R.D. | - | FCF1_MOUSE | 36260888 | 36261070 | 229221 | 16 |
| 20 | 36500293 | R.D. | - | FCF1_MOUSE | 36260888 | 36261070 | 239223 | 16 |
| 20 | 36510294 | R.D. | - | FCF1_MOUSE | 36260888 | 36261070 | 249224 | 17 |
| 20 | 36520296 | R.D. | - | FCF1_MOUSE | 36260888 | 36261070 | 259226 | 18 |
| 20 | 36530298 | R.D. | - | FCF1_MOUSE | 36260888 | 36261070 | 269228 | 16 |
| 20 | 36540300 | R.D. | - | FCF1_MOUSE | 36260888 | 36261070 | 279230 | 15 |
| 20 | 36550301 | R.D. | - | FCF1_MOUSE | 36260888 | 36261070 | 289231 | 17 |
| 20 | 36560303 | R.D. | - | FCF1_MOUSE | 36260888 | 36261070 | 299233 | 14 |
| 20 | 36570305 | R.D. | - | FCF1_MOUSE | 36260888 | 36261070 | 309235 | 17 |
| 20 | 36580307 | R.D. | - | FCF1_MOUSE | 36260888 | 36261070 | 319237 | 17 |
| 20 | 36590308 | R.D. | - | FCF1_MOUSE | 36260888 | 36261070 | 329238 | 13 |
| 20 | 36600310 | R.D. | - | FCF1_MOUSE | 36260888 | 36261070 | 339240 | 4 |
| 20 | 36610312 | R.D. | - | FCF1_MOUSE | 36260888 | 36261070 | 349242 | 17 |
| 20 | 36620314 | R.D. | - | FCF1_MOUSE | 36260888 | 36261070 | 359244 | 17 |
| 20 | 36630315 | R.D. | - | FCF1_MOUSE | 36260888 | 36261070 | 369245 | 15 |
| 20 | 36640317 | R.D. | - | FCF1_MOUSE | 36260888 | 36261070 | 379247 | 17 |
| 20 | 36650319 | R.D. | - | FCF1_MOUSE | 36260888 | 36261070 | 389249 | 17 |
| 20 | 36660321 | R.D. | - | FCF1_MOUSE | 36260888 | 36261070 | 399251 | 14 |
| 20 | 36670322 | R.D. | - | FCF1_MOUSE | 36260888 | 36261070 | 409252 | 13 |
| 20 | 36680324 | R.D. | - | FCF1_MOUSE | 36260888 | 36261070 | 419254 | 17 |
| 20 | 36690326 | R.D. | - | FCF1_MOUSE | 36260888 | 36261070 | 429256 | 16 |
| 20 | 36700328 | R.D. | - | FCF1_MOUSE | 36260888 | 36261070 | 439258 | 18 |
| 20 | 36710329 | R.D. | - | FCF1_MOUSE | 36260888 | 36261070 | 449259 | 14 |
| 20 | 36720331 | R.D. | - | FCF1_MOUSE | 36260888 | 36261070 | 459261 | 17 |
| 20 | 36730333 | R.U. | - | DRS7B_RAT | 37194208 | 37194786 | 463875 | 18 |
| 20 | 36740335 | R.U. | - | DRS7B_RAT | 37194208 | 37194786 | 453873 | 15 |
| 20 | 36750336 | R.U. | - | DRS7B_RAT | 37194208 | 37194786 | 443872 | 15 |
| 20 | 36760338 | R.U. | - | DRS7B_RAT | 37194208 | 37194786 | 433870 | 14 |
| 20 | 36770340 | R.U. | - | DRS7B_RAT | 37194208 | 37194786 | 423868 | 13 |
| 20 | 36780342 | R.U. | - | DRS7B_RAT | 37194208 | 37194786 | 413866 | 17 |
| 20 | 36790343 | R.U. | - | DRS7B_RAT | 37194208 | 37194786 | 403865 | 17 |
| 22 | 31346965 | R.D. | - | CLIP4_MOUSE | 30903241 | 30903417 | 443548 | 30 |
| 22 | 31356967 | R.D. | - | CLIP4_MOUSE | 30903241 | 30903417 | 453550 | 23 |
| 22 | 31366969 | R.D. | - | CLIP4_MOUSE | 30903241 | 30903417 | 463552 | 19 |
| 22 | 31376971 | R.D. | - | CLIP4_MOUSE | 30903241 | 30903417 | 473554 | 27 |
| 22 | 31386973 | R.D. | - | CLIP4_MOUSE | 30903241 | 30903417 | 483556 | 26 |
| 22 | 31396975 | R.D. | - | CLIP4_MOUSE | 30903241 | 30903417 | 493558 | 23 |
| 22 | 31406977 | R.D. | - | CLIP4_MOUSE | 30903241 | 30903417 | 503560 | 22 |
| 22 | 31416979 | R.D. | - | CLIP4_MOUSE | 30903241 | 30903417 | 513562 | 23 |

**Table S14.** Mean sequencing depth by chromosome (chr) for *P. crinitus, P. eremicus*, and *P. maniculatus.* Local depth for 10 kbp windows surrounding significant sweep sites on each chromosome and the number of significant sweeps identified on that chromosome (#sweep). A few *P. eremicus* chromosomes had anomalous coverage values (0 or >1000), values in brackets indicate the average coverage excluding those values (local depth) and the number of sweep sites (#sweeps) remaining after the removal of anomalous values. See footnotes for details on anomalous coverage values for *P. eremicus.*

|  | *P. crinitus* | | | *P. eremicus* | | | *P. maniculatus* | | |
| --- | --- | --- | --- | --- | --- | --- | --- | --- | --- |
| chr | mean depth | local depth | #sweep | mean depth | local depth | #sweep | mean depth | local  depth | #sweep |
| chr1 | 37 | - | - | 27 | 113 | 56 | 22 | 26 | 10 |
| chr2 | 35 | 38 | 1 | 24 | 18 | 8 | 21 | 29 | 4 |
| chr3 | 34 | 31 | 2 | 24 | 11 | 6 | 20 | 17 | 1 |
| chr4 | 35 | 29 | 11 | 27 | 165 [54]^a^ | 21 [19] | 21 | 22 | 102 |
| chr5 | 36 | 31 | 3 | 25 | 23 | 4 | 21 | - | - |
| chr6 | 33 | 33 | 8 | 25 | 18 | 11 | 20 | - | - |
| chr7 | 36 | 34 | 2 | 28 | 21 | 6 | 22 | - | - |
| chr8 | 38 | - | - | 26 | 44 | 12 | 23 | - | - |
| chr9 | 34 | 45 | 99 | 25 | 32 | 22 | 21 | 20 | 2 |
| chr10 | 33 | - | - | 24 | 109 [0]^b^ | 9 [1] | 21 | - | - |
| chr11 | 35 | 18 | 16 | 24 | 23 | 31 | 20 | 20 | 12 |
| chr12 | 38 | 36 | 3 | 24 | 9 | 3 | 20 | - | - |
| chr13 | 35 | 27 | 1 | 38 | 357 [19]^c^ | 8 [7] | 21 | - | - |
| chr14 | 35 | - | - | 24 | 10 | 1 | 21 | - | - |
| chr15 | 33 | - | - | 25 | 504 [10]^d^ | 3 [2] | 20 | - | - |
| chr16 | 33 | - | - | 22 | 19 | 1 | 21 | - | - |
| chr17 | 34 | - | - | 25 | 7 | 4 | 21 | - | - |
| chr18 | 35 | 42 | 21 | 23 | 38 | 6 | 21 | - | - |
| chr19 | 36 | - | - | 23 | 0^e^ | 1 | 21 | - | - |
| chr20 | 36 | - | - | 24 | 8 | 2 | 21 | 16 | 74 |
| chr21 | 36 | - | - | 24 | 23 | 6 | 20 | - | - |
| chr22 | 38 | 77 | 21 | 26 | 128 | 8 | 23 | 24 | 8 |
| chr23 | 43 | 48 | 19 | 27 | 9 | 4 | 25 | - | - |
| mean | 36 | 38 |  | 25 | 73 [28] |  | 21 | 22 |  |

^a^Two sites on chr4 had local coverage values > 1,000. Average local coverage across the remaining 19 sites was 54.

^b^One site on chr10 had a local coverage of 981, while the remaining 8 sites had a local coverage of 0.

^c^One site on chr13 had a local coverage of 2,719. Average local coverage across the remaining 7 sites was 19.

^d^One site on chr15 had a local coverage of 1,493. Average local coverage across the remaining 2 sites was 10.

^e^The only sweep site identified on chr19 had a local coverage of 0.

**Table S15.** Enriched GO functional groups (Biological processes, Molecular functions, Cellular components) for all *P. crinitus* significant sweep, based on the four (III), two (II), or single (I) most proximal protein-coding gene(s) (CDS) relative to each sweep. GO ID = go term identifier, Exp. = expectation, over/under = enrichment direction, Fold enrichment = observed enrichment, Bonferroni corrected p-value (< 0.05).

|  |  |  |  |  | Bonferroni adjusted p-value | | |
| --- | --- | --- | --- | --- | --- | --- | --- |
| Biological process | GO ID | Exp. | over/under | Fold enrichment | III | II | I |
| translation | GO:0006412 | 2.26 | + | 7.97 | 2.84E-07 | - | - |
| peptide biosynthetic process | GO:0043043 | 2.41 | + | 7.47 | 7.66E-07 | - | - |
| peptide metabolic process | GO:0006518 | 3.32/2.36 | + | 6.33/5.09 | 3.61E-07 | 4.87E-02 | - |
| amide biosynthetic process | GO:0043604 | 3.11 | + | 5.79 | 3.74E-05 | - | - |
| cellular amide metabolic process | GO:0043603 | 4.91 | + | 4.88 | 2.23E-06 | - | - |
| cellular amide metabolic process | GO:0043603 | 3.49 | + | 4.29 | - | 2.37E-02 | - |
| gene expression | GO:0010467 | 11.99 | + | 3.25 | 5.00E-07 | - | - |
| cellular nitrogen compound biosynthetic process | GO:0044271 | 8.28 | + | 2.9 | 2.99E-02 | - | - |
| protein metabolic process | GO:0019538 | 25.49/19.12 | + | 2.31/2.15 | 1.66E-06 | 1.87E-02 | - |
| cellular nitrogen compound metabolic process | GO:0034641 | 20.74 | + | 2.17 | 3.74E-03 | - | - |
| organonitrogen compound metabolic process | GO:1901564 | 31.83/22.62 | + | 2.17/2.08 | 3.34E-07 | 2.03E-03 | - |
| macromolecule metabolic process | GO:0043170 | 37.66 | + | 1.94 | 1.82E-05 | - | - |
| nitrogen compound metabolic process | GO:0006807 | 42.6/30.28 | + | 1.92/1.88 | 7.54E-07 | 1.19E-03 | - |
| macromolecule metabolic process | GO:0043170 | 26.77 | + | 1.87 | - | 2.10E-02 | - |
| organic substance metabolic process | GO:0071704 | 49.9/35.47 | + | 1.761.69 | 1.16E-05 | 2.37E-02 | - |
| primary metabolic process | GO:0044238 | 46.68 | + | 1.76 | 8.75E-05 | - | - |
| metabolic process | GO:0008152 | 53.75/38.21/28.17 | + | 1.73/1.70/1.74 | 5.01E-06 | 4.51E-03 | 4.50E-02 |
| Molecular function |  |  |  |  | III | II | I |
| hydrolase activity, acting on carbon-nitrogen (but not peptide) bonds, in linear amides | GO:0016811 | 0.60/0.43 | + | 13.25/13.98 | 8.89E-04 | 1.86E-02 | - |
| structural constituent of ribosome | GO:0003735 | 1.2 | + | 12.5 | 1.13E-08 | - | - |
| serine-type endopeptidase activity | GO:0004252 | 1.38/0.98/0.72 | + | 9.43/9.12/13.83 | 8.12E-06 | 2.38E-04 | 1.25E-05 |
| serine-type peptidase activity | GO:0008236 | 1.54/1.12/0.81 | + | 8.42/9.12/12.36 | 2.89E-05 | 6.44E-04 | 3.48E-05 |
| hydrolase activity, acting on carbon-nitrogen (but not peptide) bonds | GO:0016810 | 0.96 | + | 8.32 | 2.33E-02 | - | - |
| hydrolase activity, acting on acid phosphorus-nitrogen bonds | GO:0016825 | 1.58/1.120.83 | + | 8.22/8.9/12.07 | 3.78E-05 | 7.95E-04 | 4.32E-05 |
| serine hydrolase activity | GO:0017171 | 1.58/1.12/0.83 | + | 8.22/8.9/12.07 | 3.78E-05 | 7.95E-04 | 4.32E-05 |
| endopeptidase activity | GO:0004175 | 3.33/2.37/1.75 | + | 5.1/5.91/7.44 | 1.88E-04 | 4.15E-04 | 7.10E-05 |
| structural molecule activity | GO:0005198 | 4.23 | + | 3.78 | 2.05E-02 | - | - |
| peptidase activity | GO:0008233 | 4.84/3.44/2.54 | + | 3.72/4.36/5.13 | 6.62E-03 | 6.12E-03 | 4.52E-03 |
| peptidase activity, acting on L-amino acid peptides | GO:0070011 | 4.64/3.30/2.43 | + | 3.67/4.25/5.35 | 1.55E-02 | 1.84E-02 | 2.85E-03 |
| catalytic activity, acting on a protein | GO:0140096 | 16.29/11.58 | + | 2.33/2.5 | 1.98E-03 | 8.29E-03 | - |
| hydrolase activity | GO:0016787 | 18.36/13.05 | + | 2.07/2.30 | 4.52E-02 | 3.18E-02 | - |
| Cellular Component |  |  |  |  | III | II | I |
| Sin3 complex | GO:0016580 | 0.1 | + | 41.27 | 9.23E-03 | - | - |
| Sin3-type complex | GO:0070822 | 0.12 | + | 33.53 | 1.85E-02 | - | - |
| cytosolic small ribosomal subunit | GO:0022627 | 0.34 | + | 20.41 | 1.79E-04 | - | - |
| cytosolic ribosome | GO:0022626 | 0.870.62 | + | 18.34/12.90 | 4.76E-12 | 4.77E-04 | - |
| cytosolic large ribosomal subunit | GO:0022625 | 0.51 | + | 15.78 | 1.35E-04 | - | - |
| cytoplasmic stress granule | GO:0010494 | 0.49 | + | 12.19 | 2.26E-02 | - | - |
| small ribosomal subunit | GO:0015935 | 0.59 | + | 11.88 | 5.09E-03 | - | - |
| ribosomal subunit | GO:0044391 | 1.51/1.08 | + | 10.57/7.44 | 1.18E-08 | 2.38E-02 | - |
| ribosome | GO:0005840 | 1.75/1.25 | + | 9.70/7.23 | 9.13E-09 | 8.63E-03 | - |
| large ribosomal subunit | GO:0015934 | 0.96 | + | 9.36 | 1.31E-03 | - | - |
| ribonucleoprotein complex | GO:1990904 | 5.25/3.73 | + | 4.95/4.29 | 4.44E-08 | 1.77E-03 | - |
| secretory granule | GO:0030141 | 2.88//1.51 | + | 4.52/5.97 | 1.31E-02 | - | 3.37E-02 |
| cytosol | GO:0005829 | 26.24 | + | 1.98 | 9.08E-04 | - | - |
| protein-containing complex | GO:0032991 | 39.45 | + | 1.62 | 3.24E-02 | - | - |
| cytoplasm | GO:0005737 | 81.47/57.91/42.70 | + | 1.46/1.47/1.48 | 6.82E-06 | 9.37E-04 | 1.85E-02 |
| intracellular organelle | GO:0043229 | 89.06/63.31/46.67 | + | 1.44/1.45/1.48 | 8.09E-07 | 1.05E-04 | 1.17E-03 |
| organelle | GO:0043226 | 91.64/65.14/48.03 | + | 1.40/1.41/1.44 | 9.12E-06 | 5.10E-04 | 5.50E-03 |
| membrane-bounded organelle | GO:0043227 | 82.25//43.11 | + | 1.39//1.48 | 1.48E-03 | - | 1.06E-02 |
| intracellular | GO:0005622 | 101.99/72.5/53.45 | + | 1.35/1.35/1.40 | 2.46E-06 | 7.54E-04 | 9.35E-04 |

**Table S16.** Enriched GO functional groups (Biological processes, Molecular functions, Cellular components) for all *P. eremicus* significant sweep sites, based on the four (III), two (II), or single (I) most proximal protein-coding gene(s) (CDS) relative to each sweep. GO ID = go term identifier, Exp. = expectation, over/under = enrichment direction, Fold enrichment = observed enrichment, Bonferroni corrected p-value (< 0.05).

|  |  |  |  |  | Bonferroni adjusted p-value | | |
| --- | --- | --- | --- | --- | --- | --- | --- |
| Biological process | GO ID | Exp. | over/under | Fold enrichment | III | II | I |
| cytoplasmic translation | GO:0002181 | 0.3 | + | 20.27 | - | - | 7.55E-03 |
| SRP-dependent cotranslational protein targeting to membrane | GO:0006614 | 1.45 | + | 8.99 | 7.73E-05 | - | - |
| cotranslational protein targeting to membrane | GO:0006613 | 1.52 | + | 8.55 | 1.34E-04 | - | - |
| protein targeting to ER | GO:0045047 | 1.66 | + | 7.85 | 3.37E-04 | - | - |
| establishment of protein localization to endoplasmic reticulum | GO:0072599 | 1.72 | + | 7.57 | 4.95E-04 | - | - |
| viral transcription | GO:0019083 | 1.73 | + | 7.51 | 5.44E-04 | - | - |
| nuclear-transcribed mRNA catabolic process, nonsense-mediated decay | GO:0000184 | 1.81 | + | 7.19 | 8.59E-04 | - | - |
| viral gene expression | GO:0019080 | 1.99 | + | 7.04 | 3.50E-04 | - | - |
| translational initiation | GO:0006413 | 2.15 | + | 6.5 | 8.77E-04 | - | - |
| protein localization to endoplasmic reticulum | GO:0070972 | 2.08 | + | 6.26 | 3.79E-03 | - | - |
| protein targeting to membrane | GO:0006612 | 2.67 | + | 5.25 | 9.68E-03 | - | - |
| nuclear-transcribed mRNA catabolic process | GO:0000956 | 2.97 | + | 4.72 | 3.14E-02 | - | - |
| mRNA splicing, via spliceosome | GO:0000398 | 4.5 | + | 3.78 | 4.99E-02 | - | - |
| RNA splicing, via transesterification reactions with bulged adenosine as nucleophile | GO:0000377 | 4.5 | + | 3.78 | 4.99E-02 | - | - |
| mRNA metabolic process | GO:0016071 | 10.48 | + | 3.15 | 1.26E-04 | - | - |
| regulation of gene expression | GO:0010468 | 67.51 | + | 1.54 | 2.07E-02 | - | - |
| Molecular function |  |  |  |  |  |  |  |
| structural constituent of ribosome | GO:0003735 | 2.55/1.17/0.74 | + | 5.5/8.5412.08 | 1.80E-03 | 1.34E-03 | 2.57E-04 |
| RNA binding | GO:0003723 | 25.27 | + | 2.1 | 1.09E-03 | - | - |
| nucleic acid binding | GO:0003676 | 61.91 | + | 1.7 | 3.73E-05 | - | - |
| organic cyclic compound binding | GO:0097159 | 92.6 | + | 1.39 | 4.41E-02 | - | - |
| Cellular component |  |  |  |  |  |  |  |
| polysome | GO:0005844 | 0.34 | + | 14.81 | - | - | 4.39E-02 |
| cytosolic large ribosomal subunit | GO:0022625 | 0.87/0.49/0.31 | + | 10.3/16.7/19.07 | 9.12E-04 | 1.12E-04 | 1.71E-03 |
| cytosolic ribosome | GO:0022626 | 1.61/0.850.54 | + | 8.69/14.1/18.48 | 4.99E-06 | 2.44E-07 | 5.31E-07 |
| large ribosomal subunit | GO:0015934 | 1.79/0.94 | + | 5.58/8.52 | 3.39E-02 | 1.02E-02 | - |
| ribosomal subunit | GO:0044391 | 2.89/1.48/0.94 | + | 5.19/8.12/10.65 | 7.51E-04 | 8.16E-05 | 7.83E-05 |
| ribosome | GO:0005840 | 3.64/1.71/1.09 | + | 4.39/7.02/9.2 | 2.49E-03 | 3.68E-04 | 2.89E-04 |
| ribonucleoprotein complex | GO:1990904 | 10.53/5.12/3.26 | + | 3.04/4.69/5.22 | 7.51E-05 | 7.84E-07 | 4.54E-05 |
| intracellular non-membrane-bounded organelle | GO:0043232 | 30.26 | + | 1.82 | - | 5.87E-03 | - |
| non-membrane-bounded organelle | GO:0043228 | 30.4 | + | 1.81 | - | 6.41E-03 | - |
| intracellular membrane-bounded organelle | GO:0043231 | 74.83 | + | 1.4 | - | 3.55E-03 | - |
| intracellular organelle | GO:0043229 | 86.91 | + | 1.38 | - | 1.81E-04 | - |
| membrane-bounded organelle | GO:0043227 | 80.27 | + | 1.36 | - | 8.68E-03 | - |
| organelle | GO:0043226 | 89.43 | + | 1.35 | - | 6.15E-04 | - |
| intracellular | GO:0005622 | 99.54 | + | 1.31 | - | 5.31E-04 | - |

**Table S17.** Enriched GO functional groups (Biological processes, Molecular functions, Cellular components) for all *P. maniculatus* significant sweep sites, based on the four (III), two (II), or single (I) most proximal protein-coding gene(s) (CDS) relative to each sweep. GO ID = go term identifier, Exp. = expectation, over/under = enrichment direction, Fold enrichment = observed enrichment, Bonferroni corrected p-value (< 0.05).

|  |  |  |  |  | Bonferroni adjusted p-value | | | |
| --- | --- | --- | --- | --- | --- | --- | --- | --- |
| Molecular function | GO ID | Exp. | over/under | Fold enrichment | III | II | I |  |
| structural constituent of ribosome | GO:0003735 | 0.22/0.29/0.38 | + | 26.77/20.74/18.62 | 3.35E-04 | 1.38E-03 | 2.80E-04 |  |
| Cellular component |  |  |  |  |  |  |  |  |
| cytosolic small ribosomal subunit | GO:0022627 | 0.06/0.08/0.11 | + | 62.45/48.4 | 8.09E-03 | 2.79E-03 | 9.78E-04 |  |
| cytosolic ribosome | GO:0022626 | 0.16/0.21/0.27 | + | 42.97/33.3/37.23 | 6.87E-07 | 3.22E-06 | 4.74E-07 |  |
| small ribosomal subunit | GO:0015935 | 0.11/0.14 | + | 36.83/28.54/29.28 | - | 2.04E-02 | 7.20E-03 |  |
| cytosolic large ribosomal subunit | GO:0022625 | 0.16 | + | 25.19 | 3.48E-02 | - | - |  |
| ribosomal subunit | GO:0044391 | 0.28/0.36/0.47 | + | 24.89/19.29/16.96 | 4.12E-05 | 1.19E-04 | 1.81E-05 |  |
| ribosome | GO:0005840 | 0.33/0.42/0.55 | + | 21.49/19.65/14.64 | 1.23E-04 | 3.15E-04 | 4.84E-05 |  |
| ribonucleoprotein complex | GO:1990904 | 0.98/1.26/1.64 | + | 9.19/7.13/6.09 | 7.10E-03 | 5.35E-03 | 5.12E-04 |  |

**Figure S18.** Species tree estimated in *CAFE* with the total number of rapidly evolving genes in black (red = contractions, blue = expansions, absence of either color indicates 0 or none). Note that although *P. crinitus* and *P. eremicus* are depicted as near relatives, there are a number of intermediate, unsampled taxa including *P. californicus* and *P. merriami*.

**
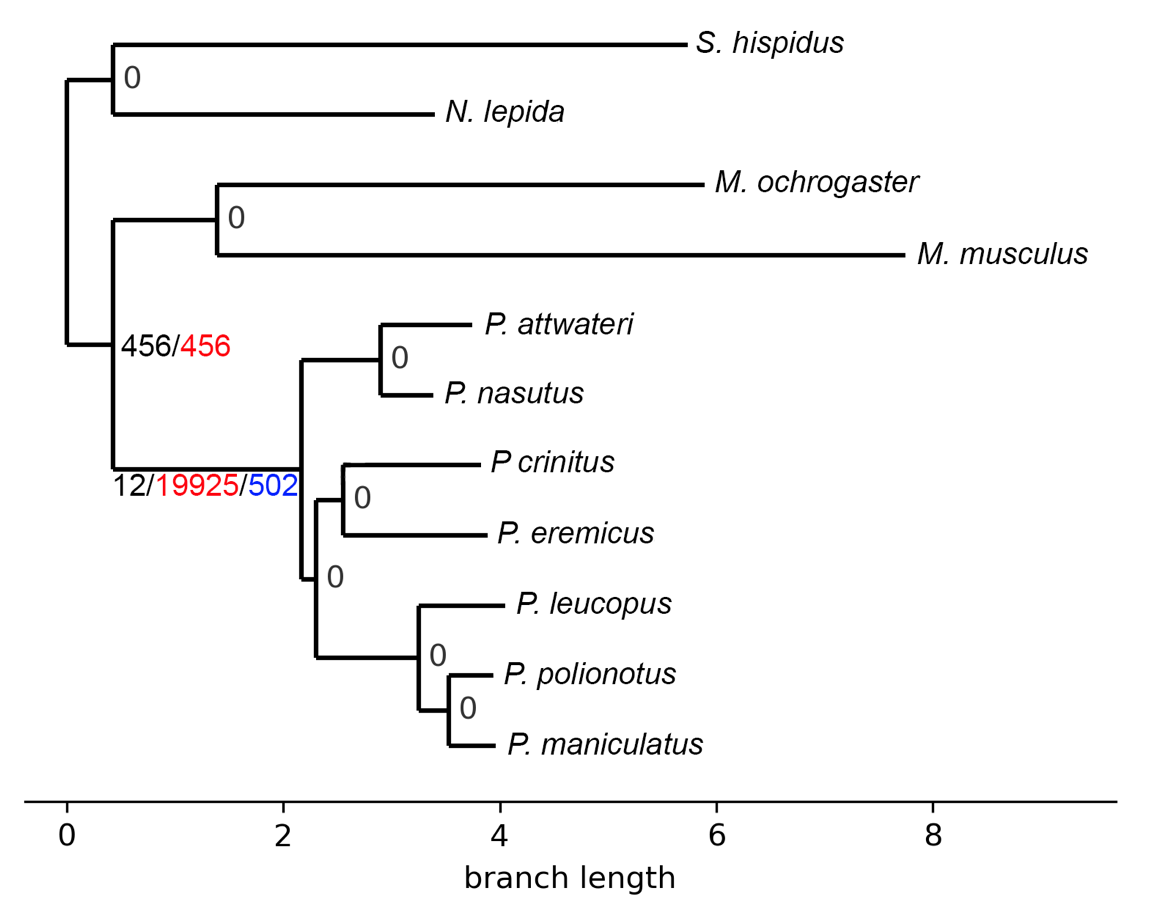
**

**Figure S19.** Non-overlapping 1kbp sliding window estimates of Tajima’s D for each *Peromyscus* species. The mean (solid blue) and three standard deviations above and below the mean (dashed red) are shown as horizontal lines. For visual clarity data points have been thinned by 25%.

**
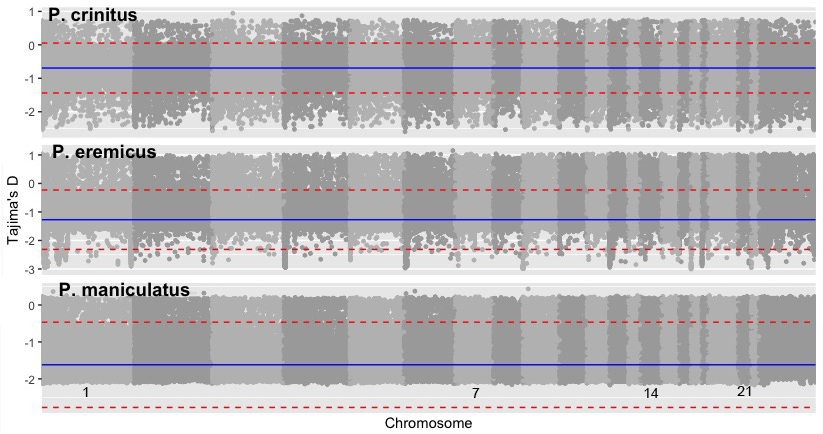
**

**Table S20.** Average estimates of π and Tajima’s D for each species globally (1 kb windows) and for 1 kb and 10 kb flanking regions surrounding significant sweep sites and *a priori* candidate loci. **Bold values** indicate a significant difference (p < 0.05) from the global mean.

| π | | | | | |
| --- | --- | --- | --- | --- | --- |
| spp. | 1k global | 10k candidate | 1k candidate | 10k sweep | 1k sweep |
| *crinitus* | 0.005 (± 0.005) | 0.005 (± 0.002) | 0.005 (± 0.004) | **0.005 (± 0.007)** | **0.006 (± 0.019)** |
| *eremicus* | 0.007 (± 0.007) | **0.008 (± 0.004)** | 0.007 (± 0.004) | **0.011 (± 0.011)** | **0.011 (± 0.011)** |
| *maniculatus* | 0.012 (± 0.010) | **0.013 (± 0.008)** | **0.014 (± 0.007)** | **0.002 (± 0.002)** | **0.003 (± 0.005)** |
| Tajima's D | | | | | |
|  | 1k global | 10k candidate | 1k candidate | 10k sweep | 1k sweep |
| *crinitus* | -0.693 (± 0.249) | **0.005 (± 0.090)** | **0.004 (± 0.079)** | **0.004 (±0.086)** | **0.002 (± 0.062)** |
| *eremicus* | -1.271 (± 0.347) | **0.013 (± 0.160)** | **0.010 (± 0.139)** | **0.018 (± 0.185)** | **0.016 (± 0.179)** |
| *maniculatus* | -1.619 (± 0.384) | **0.003 (± 0.170)** | **0.002 (± 0.166)** | **0.000 (± 0.065)** | **0.012 (± 0.220)** |

**Figure S21.** Non-overlapping 1 kbp sliding window estimates of nucleotide diversity (π) for each *Peromyscus* species, corrected by the number of variable sites per window. The mean for each species is represented by a solid blue line and three standard deviations above the mean is shown by a red hashed line. For visual clarity data points have been thinned by 50%.

**
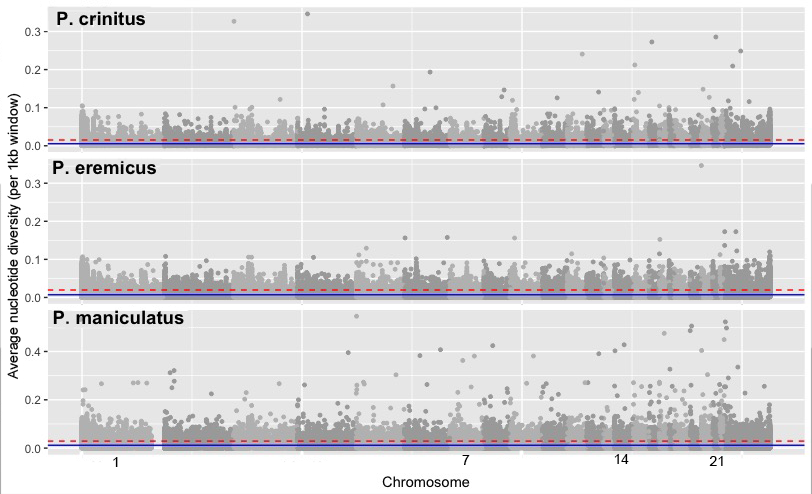
**
